# Next of kin involvement in mental health care services – a systematic participatory overview of evidence syntheses

**DOI:** 10.1186/s12888-026-08313-5

**Published:** 2026-06-30

**Authors:** Peggy Prien, Sebastian Bayer, Susanne Kappesser, Nora Dietrich, Sven Speerforck, Johanna Kummetat, Sarah Schernau, Silvia Bahl, Franziska Vosseberg, Kolja Heumann, Sebastian von Peter

**Affiliations:** 1 Department of Psychiatry, University of Leipzig Medical Center, Leipzig, Germany; 2 Brandenburg Medical School, Immanuel Center of Mental Health, Ruedersdorf, Germany; 3 Berlin Association of Relatives of People with Mental Illnesses (ApK LV Berlin e.V.), Berlin, Germany

**Keywords:** Caregiver involvement, Mental health, Caregiver, Psychiatry, Involvement, Participation

## Abstract

**Background:**

The importance of the involvement of next of kin (NOK) in mental health treatment settings is widely recognized. Yet, interventions to involve NOK are not sufficiently implemented in clinical practice – a situation that the German-based collaborative-participatory research project “Involvement of Next of Kin in Psychiatric Care” (PazAng) aims to change. This overview intends to systematically collect and evaluate the existing evidence syntheses (=ES) on interventions, programs, and approaches for the involvement of NOK in the field of mental health care to create a foundation for their improved implementation in future.

**Methods:**

The study follows a collaborative-participatory approach to ensure the grounding of the results in diverse and experiential perspectives throughout the research process. An overview of ES is conducted, focusing on the types of interventions and mental disorder for which NOK involvement has been evaluated, the types of outcomes assessed, and the inferences to be drawn on the effectiveness.

**Results:**

The search identified 1340 publications of which 50 ES were included after full text screening. Interventions were heterogenous in relation to their theoretical underpinnings, components, setting, delivery format, the delivering staff, duration, and frequency. The component most assessed was psychoeducation. Outcomes varied widely, especially for the NOK involved. Overall, positive effects on both NOK and PIT were reported.

**Conclusion:**

The heterogeneity of NOK interventions relate to fundamental contingencies: first, to individuum-centrism of the mental health care system with a strong bias towards individualized over social-relational explanation models. Second, to a lack of research on multi-person setting interventions that also pay attention on how to renumerate and institutionally implement them in sustainable ways.

**Prospero ID:**

CRD42024595639

**Supplementary Information:**

The online version contains supplementary material available at 10.1186/s12888-026-08313-5.

## Introduction

The importance of next of kin (NOK) participation in psychiatric and psychosocial treatment settings is widely recognized both worldwide and in Germany [1, 2]. The wellbeing and quality of life of NOK in these settings is often strained on multiple levels [3, 4]. One significant challenge is the economic burden of the support work by NOK [5]. This work also imposes physical [6], psychosocial burden and loneliness [7]. Furthermore, NOK often encounters courtesy stigma [8], also known as affiliate stigma [9]. Addressing these and other challenges requires comprehensive support systems that consider the multifaceted impacts of NOK work and match their needs. In addition, and in relation to the persons in treatment (=PIT), their NOK are important resources, and their systematic participation facilitates recovery and may also improve outcomes such as re-hospitalization rates, risk of relapse, and patients’ satisfaction [10, 11]. The correlation between NOK participation and improved outcomes likely pertain particularly to PIT during an acute crisis [12]. Early NOK participation may play an essential role in promoting recovery, in line with the position statement of the UN Convention on the Rights of Persons with Disabilities [13].

The significance and urgency of NOK participation is demonstrated by recent guidelines, various publications by associations of PIT, NOK, and mental health professionals, and recommendations from national and international organizations [12–15]. In contrast, NOK interventions are not sufficiently implemented [16–22], by which we mean a lack of systematic introduction of these interventions in routine clinical practices [23]. For instance, a cross-sectional survey that was undertaken in 22 countries by the European Association of Families of People with Mental Illness (EUFAMI) found that most NOK do not feel sufficiently valued by mental health professionals, and more than 40% of NOK are dissatisfied with their participation in the treatment and their ability to influence important decisions [15]. In its recommendations, EUFAMI demands that 1) health professionals must be better informed to include NOK in their various participating roles, and 2) that policy makers should incorporate NOK interventions into national mental health care plans (ibid). This conclusion was echoed by the Declaration on Quality of Psychiatry and Mental Health Care of the European Psychiatric Association (EPA), which likewise emphasized the importance of close collaboration between practitioners and NOK in working towards the prevention and treatment of mental illness [14].

This overview is part of the research project “Involvement of NOK in Psychiatric Care” (PazAng study) funded by the German Research Foundation (DFG). This study follows a collaborative-participatory approach to ensure the grounding of the knowledge production in diverse and experiential perspectives throughout the research process by bringing researchers with different professional backgrounds and with and without lived experiences of NOK work. It pursues the aim to advance the involvement of NOK in clinical practices in German mental health care services and beyond. For this purpose, and as a first step to reach this aim, this overview intends to systematically collect and evaluate the existing evidence syntheses (=ES) on interventions, programs, and approaches for the involvement of NOK with the aim of creating a comprehensive foundation for their improved implementation in future. Thereby, it addresses the following research questions:

1. What types of interventions of NOK involvement in mental health care have been studied, and what are their key characteristics (components, settings, delivery formats, duration, frequency, implementing staff)?
2. For which types of mental disorder and kinship relationships have these interventions been evaluated, and which outcomes have been assessed?

As the certainty of evidence was low – as stated below, owing to the heterogeneity across studies, risk of bias, imprecision, and the limited number and variable quality of meta-analyses – only limited and non-generalizable conclusions are drawn about effectiveness. Given this finding, the discussion section offers recommendations for further studies and evidence synthesis.

## Methods

We followed the reporting guideline for overviews of reviews of healthcare interventions (PRIOR) [24] (checklist 1) and also included a PRISMA checklist (checklist 2). The overview was registered at PROSPERO (CRD42024595639).

The various steps described below were implemented mainly by the PazAng review sub-team (PP, SK, KH, SeB, SvP), which had been supported in various phases of the review process by the entire PazAng research team (SiB, ND, LG, JK, SS) and a steering board who, in three sessions, gave advice from different perspectives: lived experience as NOK (*N* = 2), a combination of lived experiences as PIT and researcher (*N* = 2), and expertise as researcher and practitioner (*N* = 3).

This review adopts a constructivist paradigm in which knowledge and knowledge production are understood as socially constructed and context-dependent [25]. Against this background and considering the available data, it is less focused on the aggregation of quantitative findings, instead pursuing an interpretative synthesis that considers the influence of divergent discourses, definitions, and perspectives during the process of knowledge production.

### Wording

The PazAng team and participatory steering board decided on the term *next of kin (=NOK)* to refer to individuals who have a close and trusting relationship with a person undergoing mental health treatment. Kin are not solely understood as biological relatives but also as relationships beyond ancestry or genealogy. Other terms, such as *caregivers,* were rejected, as they focus on responsibilities instead of distinct roles. Further terms such as *relative* or *family* risk excluding friends or other trusted individuals. Despite considering alternative terms (*service user, patient, symptom carrier)*, it was decided to use *person in treatment (=PIT)* to describe individuals receiving psychiatric care, as it highlights the relationship between the three key stakeholders: psychiatric staff, PIT, and NOK.

### Search strategy

A combined search in Medline (via PubMed) and Epistemonikos complemented by reference checking had proven to be a resource-saving option with high sensitivity [26]. This strategy was complemented by additional searches in PubPsych. The search was not limited by year of publication. The search terms covered the domains of (1) diagnosis of mental disorders, (2) NOK involvement, (3) interventions/ programs. For more information on search terms see supplement 1. Given the diverse framings used, the entire PazAng research team and participatory steering board developed as many search synonyms as possible in relation to domains 1 and 2 (see Box 1).

### Eligibility criteria

The in- and exclusion criteria were developed first within the review team and then discussed in the entire PazAng research team and with the members of the steering board (see supplement (S=2).

*Study type*: We included ES with at least a systematic search strategy, with or without meta-analysis and regardless of the type of included studies.

*Population*: We included ES from the research field of mental health, focusing on outcomes for either PIT and/or their NOKs. Further, we included ES on individuals diagnosed with mental health conditions of any diagnostic categories alongside ICD-10/11 and DSM-5. In the case of a mix of mental and other health problems, only studies with at least 20% primary studies on mental health problems were included. Further, this study focuses on adult NOK and PIT, considering the significant differences in history, settings, and requirements for child and adolescent psychiatry. However, ES that included NOK under 18 years were considered when involvement was reported.

*Interventions*: We included ES that reported on all types of interventions, programs, and approaches that aim to systematically involve NOK. “Systematic” means the planed, intentional, structured nature of the involvement, versus, for example, a spontaneous conversation with NOK of-the-moment. “Involvement” is used as an overarching term to encapsulate various collaborations between NOK and PIT alongside the various stage modes of participation [27], such as their active participation, information, consultation, or other forms of including them in mental health care.

*Control*: No exclusion criteria.

*Outcome*: No exclusion criteria.

### Study selection

All members of the review team independently screened the titles and abstracts of the sources identified. Disagreement was discussed until consensus was reached. Included sources were screened and coded in full text by two members (PP, SeB).

### Data extraction

An extraction form was developed within the review team and modified by the full team. It was piloted and iteratively modified during the extraction process. The final categories can be found in S5. Data extraction was carried out by one reviewer (PP) and checked by a second one (SeB).

### Quality assessment

To assess the methodological quality, we used the AMSTAR-2 tool [28]. Due to the shortage of resources and the number of studies included, only 10% were assessed independently by two reviewers (PP, SeB). Dissent was discussed and a joint approach agreed upon. All other ES were assessed by one reviewer (PP).

### Data synthesis

Given the descriptive focus of this overview, the synthesis of data was done narratively. Preliminary categories were developed within the review team during extraction and presented to the entire team to refine them. The complete results again were presented to the entire team to discuss them in relation to their diverse expertise and against the results of the other PazAng sub-projects.

## Results

**Fig. 1:**
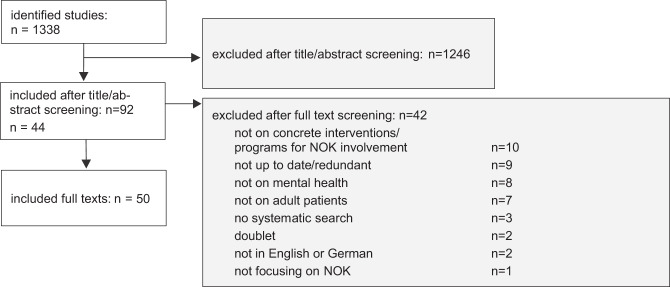
Flowchart of the screening process

We searched Pubmed, Epistemonikos and Pubpsych on October 16th 2023 and identified 1340 sources. After screening titles and abstracts, 93 studies met the inclusion criteria. By full text screening, further 43 studies were excluded, most commonly as they had not reported on NOK interventions, included persons < 18 years as PIT, or did not focus on mental health (see Fig. 1). A list of all excluded ES with corresponding rationales can be found in S3. Finally, a total of 50 ES was included.

This amount of ES includes an estimated totality of 992 primary studies, including 495 randomized controlled studies, the remaining studies covering a variety of designs. These numbers are approximations, as the number of overlapping studies could not be defined without a more extensive screening on the level of primary studies. A more detailed screening process was only applied in relation to the ES on focusing the PIT diagnosis of schizophrenia, revealing that only 9 out of 142 primary studies were included in more than 2 ES (see S8). Tentatively generalizing from these results, a low level of overlap may be inferred.

### Methodological quality of the ES

Due to the various types of ES, the AMSTAR2 criteria were not applicable in all cases. The methodological quality was very poor (see Table 1 and S4). Only 8 ES met all items, while 14 ES met solely the item “comprehensive literature search”. The quality of the primary studies (if assessed) was often described to be insufficient. Most commonly, a lack of comparative studies and especially randomized controlled trials were reported.

**Table 1:** Study characteristics

Source	AMSTAR2 rating	Disorder	Intervention (title)	Caregiver type	Body of evidence	Patients’ outcomes	Caregiver/family outcomes	Limitations	Results (authors conclusion)
Al-Sawafi et al. (2020) [29]	1/5 critical items not met	Psychotic disorders	Psychosocial family interventions	Family/caring relatives	6 studies (3 RCTs) countries: Jordan, Egypt	Symptoms, medication adherence, social functioning, quality of life and knowledge of schizophrenia	Family burden of care, attitude and carers’ quality of life	Small number of studies, small population, high PICO heterogeneity, poor study quality, poor reporting/ lack of information	*“Any family-oriented intervention for schizophrenia is likely to be better than standard care in improving the outcome for patients and their families”*
Ashcroft et al. (2018) [30]	5/7 critical items not met	Schizophrenia	Caregiver-directed psychosocial interventions	Family/close relationship	18 RCTs countries: not reported	Relapse, suicide attempts, emergency service utilization, medication compliance, clinical activities compliance	Not reported	Small sample sizes, heterogeneity of outcome definitions, heterogeneity of intervention duration and frequency	*“Caregiver-focused psychosocial interventions are associated with reductions in hospitalization, relapse, and treatment non-compliance”*
Bademli et al. (2011) [31]	4/5 critical items not met	Schizophrenia	Family to family support programs	Family	12 studies countries: not reported	Not the focus of the review	Family Support, Family Burden, Empowerment Scale, Depression, Quality of Life, Information Needs, Caregiving Satisfaction, Knowledge, Self Efficacy	Very poor reporting	*“… although program content was designed to increase coping skills of caregivers, they did not examine the effect of the program on the coping ability of the of care givers …”*
Barbeito et al. (2020) [32]	3/5 critical items not met	Miscellaneous (severe mental illness; mostly psychotic disorder)	Online interventions for families	Family relatives	9 studies (4 RCTs) countries: not reported	Symptoms, perceived stress, social support, hospitalisations	Acceptability, feasibility, usefulness, family knowledge, perceived stress, burden (relational problems, mental health and difficulties with daily activities), self-compassion, social support	Few studies, few RCTs, partly only on feasibility, high PICO heterogeneity	*“Online intervention programs are superior to standard of care (with respect to reducing caregivers’ symptoms, such as perceived stress and burden), help to improve knowledge, and enable experiences to be shared. … patients … experienced relief from their symptoms (positive) …, had fewer hospitalizations, and seemed to have a higher perceived level of social support and good acceptance of online programs.”*
Baruch et al. (2018) [33]	2/7 critical items not met	Bipolar disorder	Psychological interventions (focussing on caregiver wellbeing)	Relatives, spouses, partners, friends and neighbours	9 studies (8 RCTs), 8 included in meta-analysis countries: 2x USA, Brazil, Italy, Australia, Greece, Ireland, Spain, The Netherlands	Not focus of the review, but still reported: functioning, symptomatology. Note: In 3 studies, patients’ outcomes are reported even though they did not participate.	Subjective/objective burden, psychological symptoms, knowledge, quality of life, self-esteem, health risk behaviour, psychosocial problems, social support, attitudes and family relationships.	Heterogeneity, wide confidence intervals, methodological limitations, diversity of measures used, clinical and community samples may not be comparable, small sample sizes, small study size	*“Evidence for the efficacy of psychoeducation in im-proving caregiver burden at post-treatment, and knowledge at post-treatment and follow-up. The lack of an effect for psychological symptoms could suggest that more targeted interventions are needed to address the needs of caregivers experiencing greater levels of distress.”*
Baudinet et al. (2021) [34]	4/5 critical items not met (limited applicability of AMSTAR2 to scoping reviews)	Eating disorder	Multi-family therapy	Family and caregivers	27 studies (3 RCTs) trials on adults countries: UK, Canada, USA, Sweden, Belgium, Denmark, Czech Republic, Norway	BMI, eating disorder psychopathology	Expressed emotion, general wellbeing, negative/positive aspects of caregiving, perceived burden, perceived social supports, stigma	No exhaustive review of all MFT models, small sample sizes, study methodologies too diverse, sample too homogenous (predominantly white and female), only little data on bulimia nervosa	*“The current review suggest MFT is an effective treatment for anorexia nervosa and leads to improvements in individual, as well as some caregiver and family factors.”*
Brady et al. (2017) [35]	2/5 critical items not met	Depression (major depressive disorder)	Family psychoeducation	Family, spouses	8 studies (5 RCTs) countries: Japan, Italia, Belgium, Indian, Germany	Symptoms and functioning	Family attitude, burnout, distress, negative consequences of care giving	Few RCTs, no attendance reporting, nearly no follow-up outcome reporting, heterogeneity in assessed measures, methodological limitations	*“The results from this systematic review highlight the promising outcomes that may be gained by offering FPE for depression for both the patient and their family–carers. The findings also provide preliminary evidence that MFPE may be as effective as SFPE for patient outcomes and may be potentially more effective for family–carers.”*
Camacho-Gomez et al. (2020) [36]	2/7 critical items not met	Psychosis	Family intervention	Family	11 studies, RCTs only; *n* = 1360 countries: not reported; 5 Asia, 5 Europe, 1 Australia.	Relapse, days of hospitalization, symptom severity, functionality	Not reported	High PICO heterogeneity in domains (i.e. different relapse definitions), no blinding of participants and personnel (partly blinding for outcome assessment)	*“Family Intervention for psychosis … is effective for reducing relapse rates, duration of hospitalization, and psychotic symptoms, and for increasing functionality”*
Davies et al. (2021) [37]	2/5 critical items not met	Miscellaneous (mental illness)	Interventions to support help-seeking	Young people	11 studies (8 on interventions) countries: not reported; Australia, Europe, UK	Not the focus of the review	Help-seeking	Paucity of experimental and pre-post designed studies, does not account for parental disorders, heterogenous outcome measures, small number of studies	*“Schools and communities have the potential to be key agents in negating the pervasive stigma towards mental illness and also facilitating help-seeking behaviours.”*
Dehbozorgi et al. (2023) [38]	4/5 critical items not met	Miscellaneous (chronic mental illness)	Family-centred collaborative care	Family	15 studies (11 RCTs) countries: 4x USA; 1x Colombia, 2x Australia, 1x India, 4x UK, 3x Iran, 1x Thailand, 3x Spain, 1x Turkey, 1x China, 1x Pakistan, 1x Ethiopia, 1x Canada, 1x America, 2x NA	Relapse rate, re-hospitalization, social functioning, social cognition, quality of life, general well-being, symptoms, adherence, maladaptive emotional schemes	Family functioning, family relationship functioning, caregiver burden, guilt, empathy	Differentiation between family interventions and FCCC was challenging, narrative reporting for single studies, no/nearly no synopsis, contradictory statements within the review, unclear statements probably due to English language problems, methodological poor	*“… family involvement in the care of patients with chronic mental illness affects no recurrence of the disease, and no re hospitalization of patients with this disorder”*
Dirik et al. (2017) [39]	All (3/3) critical items met (limited applicability of AMSTAR2 to qualitative reviews)	Miscellaneous (severe mental illness, acute phase)	Miscellaneous concepts	Family	“16 key articles” countries: Canada, France, UK, USA, Germany, Finland	Not the focus of the review (qualitative methodology)	Not the focus of the review (qualitative methodology)	Findings are not exhaustive in relation to all existing family involvement models, unclear translation to other settings (rural environments, more traditional family structures)	*“Although family involvement models have been developed in the context of diverse theoretical perspectives and sociopolitical events, there are many commonalities in their components.”*
Eassom et al. (2014) [40]	1/3 critical items not met (limited applicability of AMSTAR2 to qualitative reviews)	Psychosis	Miscellaneous concepts	Family, not specified	43 studies (qualitative, all types) countries: 50% UK, others Finland, USA, Italy, Australia, Canada, Germany, India, Ireland, New Zealand, Spain, Greece, Portugal	Not the focus of the review (qualitative methodology)	Not focus of the review (qualitative methodological)	Often very small populations/case studies	*“Family work can only be implemented if this is considered a shared goal of all members of a clinical team and/or mental health service, including the leaders of the organisation. This may imply a change in the ethos and practices of clinical teams, as well as the establishment of working routines that facilitate family involvement approaches.”*
Esteban et al. (2022) [41]	4/5 critical items not met	Substance use disorder	Family therapy	No caregivers	18 (15 RCTs), only 5 trials on adults countries: USA, Spain, Netherlands, Germany, Europe, Chile	Number of days abstinent, daily methadone dosage	Family functioning	Small sample sizes, more adolescent than adult samples, women represent only small percentage in most included studies, some studies don’t report number and/or duration of sessions, many studies relied on self-reported measure of substance use, many studies were conducted in United States which limits generalization to other countries and cultures, many variations of FT included	*“. the incorporation of family members in the treatment of substance abuse produces benefits in users and the family functional system.”*
Fleming et al. (2020) [42]	2/3 critical items not met (limited applicability of AMSTAR2 to scoping reviews)	Eating disorder	Miscellaneous concepts	Family	68 studies (50 clinical studies, 8 reviews, 10 grey literature sources) countries: UK (17), United States (12), Canada, Italy, France, Germany, Spain, Sweden, The Netherlands, Iceland, Australia	Not the focus of the review (qualitative methodology)	Not the focus of the review (qualitative methodology)	Limited number of effectiveness studies, heterogeneity of research designs, large range of settings and instruments, absence of studies that evaluated long-term outcomes, dearth of research identified into FIT approaches with family of choice, rather than family of origin, or with remote family members included via new technologies.	*“Results confirmed that family members of adults were willing to be involved with eating disorder treatment services and appeared to respond to interventions of varying intensity and duration. The impact on individual patients, and effect on treatment outcomes, are yet to be established.”*
Frey et al. (2022) [43]	4/5 critical items not met	Suicide (ideation and behaviour)	Family-based treatments	Family	22 studies (16 RCTs) USA (17), Germany (1), Norway (2), Australia (1), not reported (1)	Self-harm behaviour	Family functioning	Family- based interventions are non-existent (in published research) for age groups outside of adolescence, family- outcome measures only reported if an interaction effect was tested in relation to treatment outcomes, no quality assessment of included studies	*“We identified two well-established intervention categories that met the highest standards for interventions and three probably efficacious intervention categories. All interventions found focused solely on suicide risk in adolescent populations.”*
Gracio et al. (2016) [44]	2/3 critical items not met (limited applicability of AMSTAR2 to qualitative reviews)	Psychosis	Family Interventions	Family; not specified	22 studies (qualitative, all types) countries: not reported	Not the focus of the review (qualitative methodology)	Not the focus of the review (qualitative f methodology)	Review does not fulfil methodological requirements for systematic review	*“We found that being in a therapeutically supportive relationship, followed by education about the illness, and later coping skills training, look to be the major active ingredient at different levels of intervention.”*
Hansen et al. (2022) [45]	1/7 critical items not met	Miscellaneous (not only mental disorders)	Mental health interventions (focussing on caregiver wellbeing)	Spouse, child, partner, parent, and/or other members of the family	44 RCTs (31 in meta-analysis, *n* = 1899; 15 on severe mental illness) countries: not reported	Not the focus of the review	Depression, anxiety, stress, subjective burden, quality of life, psychological distress	Small sample sizes, substantial statistical and PICO heterogeneity because of the different and inconsistent component descriptions, no subgroup analyses, different self-report questionnaires to measure psychological distress, risk of bias	*“The evidence supports that several interventions improve the mental health of caregivers. Manualized interventions ≥ 8 weeks with active participation are most effective.”*
Henken et al. (2007) [46]	All critical items met	Depression	Family therapy	Family	6 RCTs countries: USA (5), Australia (1)	Depression	Family functioning, family attitude, family cohesion, coping, parental outcome measures	Very heterogeneous in terms of interventions, participants, and measuring instruments, not only depression, not only adult users	*“Current evidence base is too heterogeneous and sparse to draw conclusions on the overall effectiveness of family therapy in the treatment of depression.”*
Higgins et al. (2020) [47]	1/5 critical items not met	Miscellaneous (mental health difficulties)	Group psychoeducational initiatives	Family	8 studies countries: USA (4), United Kingdom (2), Sweden (1), Australia (1)	No details reported, in 2 studies reported for both family members and service users, in 3 studies for service-users only	No details reported; in 2 studies reported for both family members and service users, in 3 studies for family-members only	Some degree of variability in relation to the factors identified, small sample sizes, the convenience nature of the sampling, diverse range of settings, heterogeneity among the target audience for the intervention (service users/family) as well as combining peer or professionally led interventions, subjective self-report measures, collecting data prior to or immediately post intervention (lack of long-term sustainability)	*“Implementing evidence-based group psychoeducation interventions is a complex process as a broad range of factors across multiple levels affect implementation outcomes … Without this form of evidence, it is unlikely that interventions like group psychoeducation will be added and embedded as a treatment option across all mental health services.”*
Krysinska et al. (2021) [48]	All critical items met	Suicide (attempt)	Psychosocial interventions (focussing on caregiver wellbeing)	Family members and other informal support persons	7 studies (3 RCTs); 3 for caregivers, 4 for suicide attempters but assessing family outcomes countries: UK (2), USA (2), South Korea (1), Sweden (1), Taiwan (1)	Not the focus of the review	Burden, distress, family functioning, mental health, quality of life	Small number of studies, heterogeneity of the outcomes reported, weak study quality, no data on effectiveness of interventions for siblings and informal carers beyond the family	*“Psychosocial interventions designed specifically for informal carers seem to lower their burden of care and improve the ability and willingness to care for a suicidal family member.”*
Lohrasbi et al. (2022) [49]	4/5 critical items not met	Miscellaneous (chronic mental disorders)	Interventions to promote psychosocial health (focussing on caregiver wellbeing)	Family	19 studies (13 RCTs) countries: not reported	Not the focus of the review	Stigma, burden of care, and also in promoting family tolerance, quality of life, emotional regulation, psychological symptoms	Superficial synopsis, methodologically poor	*“Using a collaborative approach, mental health service providers and government systems can help improve the psychosocial health of caregivers. The related managers and policymakers can reduce the emotional and psychological burden of families and promote their psychosocial health through developing a comprehensive program including practical objectives and strategies.”*
Ma et al. (2017) [50]	1/7 critical items not met	Psychosis (recent onset)	Family interventions (focussing on caregiver wellbeing)	1st and 2nd degree relatives (no friends or sprouts)	12 RCTS, 9 in meta-analysis countries: UK, Spain, Australia, Hong Kong	Not the focus of the review	Care burden, family functioning, formal support services utilization, family perceptions of social support, caregiving experience, social problem-solving ability, service satisfaction, psychological distress, expressed emotion, general health	58% of RCTs from Hongkong, one reviewer = lead author of some of the included studies, lack of studies, small population	*“. Cognitive behavioural family intervention is superior to TAU in treating positive and negative symptoms immediately following the intervention.”*
Ma et al. (2020) [51]	2/7 critical items not met	Miscellaneous (severe mental illness)	Cognitive behavioural family intervention	Family relatives	4 RCTs countries: 2x Pakistan, Spain, UK	Positive/negative symptoms, delusions, hallucinations, psychopathology, insight	Reported in 1/4 studies, depression, anxiety, coping ability, self-esteem	Few numbers of studies, only 2 studies in meta-analysis	*“…. Cognitive Behavioural Family Intervention is superior to TAU in treating positive and negative symptoms immediately following the intervention.”*
Macleod et al. (2011) [52]	4/5 critical items not met	Schizophrenia	Miscellaneous (nurse-led)	Carers (not specified)	68 comparative studies countries: not summarized, all continents; many from UK, US, China	Not the focus of the review	Knowledge, social support, coping, burden, distress, self-efficacy, health	High PICO heterogeneity, esp. for interventions, no structured quality rating, small populations	*“A combination of education, mutual support and coping strategies delivered within an intensive community programme could effectively reduce carer burden and improve health and coping. Nurses could also deliver support to carers within structured intensive programmes.”*
McGovern et al. (2021) [53]	1/5 critical items not met	Substance use disorder	Psychosocial interventions (focussing on caregiver wellbeing)	Family	58 studies countries: USA; 5 Iran; 4 Australia; 3 UK, 3 Sweden; 2 Germany, 2 Spain; each 1 Brazil, Korea, Vietnam, the Netherlands, Mexico	Not the focus of the review	Social outcomes (34 trials), psychological outcomes (*n* = 27 trials), health outcomes (8 trials). partner violence, relationship satisfaction, stability and family functioning	High risk of allocation and performance bias, small sample sizes, not sufficiently powered to conduct reliable statistical testing or cost effectiveness analysis	*“There is a large volume of literature examining psychosocial interventions … However, these interventions do not go far enough to address the needs often experienced within substance-affected families.”*
Meis et al. (2013) [54]	3/7 critical items not met	Miscellaneous (mental health conditions)	Family involved psychosocial treatments	Not specified	39 RCTs (51 references) countries: only US studies	Symptoms, service utilization, days abstinent for alcohol and drug use, treatment initiation, substance use, relationship adjustment, relapse, hospitalization, global functioning, treatment initiation, attendance, medication adherence …	Not summarized, but reported for the individual studies, selection: burden, distress, anxiety, family/ couple functioning, intimate partner violence, communication /conflict	Limitation to US studies, poor study quality, trials conducted on mostly white and male samples who were under 40 years old, a lot of exclusion criteria, heterogeneity	*“… behavioural couple or family therapy (BCT/BFT) reduced substance use (small-to-moderate effects) and improved relationship adjustment (large effects) compared to individually-oriented treatments. Community reinforcement and training (CRAFT) increased treatment initiation three-fold but did not improve substance use or family functioning over alternative family interventions. Family focused therapy for bipolar disorder improved symptoms over less intensive treatments with mixed findings when compared to equally intensive treatments. For both bipolar disorder and schizophrenia spectrum disorders, the few trials meeting our search criteria and heterogeneity among trials precluded generating broader conclusions …”*
Meyer et al. (2017) [55]	1/3 critical items not met (other items not applicable as no studies were identified)	Miscellaneous (severe mental illness, mostly psychotic disorder)	Novel technology (focussing on caregiver wellbeing)	Not specified	No studies found	No studies found	No studies found	No studies found	*“none of those studies focused on caregivers, and the ones we identified using mobile or web-based applications were just for patients and not their relatives”*
Morillo et al. (2022) [56]	1/5 critical items no met	Psychotic disorders	Interactive mobile or web-based software	Family (not described in detail)	27 studies (12 RCTs) countries: 11 China, 4 India, 2 Iran, 2 Vietnam, 2 Egypt, 1 South Africa, 1 Brazil, 1 Indonesia, 1 Thailand, 1 Nicaragua, 1 Pakistan	Recovery rate, symptom severity (negative symptoms), relapse rate, attendance rates, service user satisfaction, knowledge about schizophrenia, self-care skills, expressed emotion, social and occupational functioning, psychosocial functioning, unclear, whether some of the outcomes were measured patients and/or family members	unclear, whether some of the outcomes (knowledge, expressed emotion, functioning) were measured (also) for the family members	High PICO heterogeneity, mostly high risk of bias. absence of treatment as control seems inadequate	*“There were positive health impacts across four outcome domains.”*
Morton et al. (2021) [57]	3/5 critical items not met	Suicide (attempt/risk of suicide)	Family-based interventions	Family and friends	17 studies (6 RCTs) countries: USA (11), Australia (3), Taiwan (2), Canada (1)	Not found in primary studies	knowledge, self-efficacy, gatekeeper-related skills, attitude; behavioural change	diversity of study designs and outcome measures, some studies did not report the details of assessment questions, the specific program components that respond to the needs of family and friends remain poorly evaluated	*“Gatekeeper training programs … have a positive impact on improving subjective knowledge …, while also providing increased subjective self- efficacy and confidence in helping an individual at risk of suicide.”*
NICE (2018) [58]	All critical items met	PTSD, at risk for PTSD	Psychological and psychosocial interventions (focussing on caregiver’s wellbeing)	Family (incl. Parents of children)	6 RCTs (2x at risk for PTSD, 4x PTSD) USA (+Canada) (6)	Not the focus of the review	Mental health, depression, anxiety, quality of life, relationship satisfaction	Few studies, few outcomes available, poor study quality, inconsistent effects	Not available in evidence review; five recommendations on involving and supporting families and carers: *“- Consider providing information and support …* *- Involve family members and carers, if appropriate, in treatment …* *- Consider providing practical and emotional support and advice …* *- Think about the impact of the traumatic event on other family members …* *- For members of the same family who have PTSD after experiencing the same traumatic event, think about what aspects of treatment might be usefully provided togethe*r” http://www.nice.org.uk/guidance/ng116/chapter/Recommendation
Norton et al. (2021) [59]	1/5 critical items not met	Miscellaneous (mental health)	Family recovery interventions	Family	3 studies countries: all UK	Not the focus of the review (descriptive focus)	Not the focus of the review (descriptive focus)	Poor quality of studies, UK only	*“The results of this review mirror much of the cultural and organisational attitudes towards families of those with mental health challenges.”*
Okbokoro et al. (2014) [60]	All critical items met	Schizophrenia	Family intervention (brief)	Family (diversely defined)	4 RCTs, *n* = 163 users countries: US, UK, India	Hospital admission, relapse	Understanding schizophrenia, burden, distress, crisis, emotional involvement and criticism	High PICO heterogeneity, poor study quality, few studies	*“No power to inform clinical practice, […] larger studies needed.”*
Petkari et al. (2020) [61]	2/5 critical items not met	Miscellaneous (severe mental illness = psychotic disorder)	Miscellaneous (discharge planning)	Family members and friends (informal carers)	14 studies (8 RCTs, 2 NRCTs) countries: USA (7), UK (3), Australia (1), Taiwan (1), Iran (2)	Re-admissions, relapse, treatment adherence, compliance, treatment satisfaction, medication side effects, functioning, social skills	Functioning, burden, health status, knowledge, availability of support, expressed emotion	Heterogeneity of the treatment models and of the outcomes assessed, different countries with different health care systems	*“The most comprehensive interventions, i.e. those including psychoeducation, care planning and aftercare follow-up were better evaluated and showed a clearer benefit in improving long-term outcomes and, in particular, reduce re-hospitalization”*
Pharoah et al. (2010) [10]	All critical items met	Schizophrenia	Family intervention	Family	53 RCTs countries: Australia (2), Canada (1), Europe (12), China (28), USA (10)	Relapse, admission, compliance with medication, suicides, social impairment	Not summarized, but reported for the individual studies, expressed emotion, coping, burden, experiences, satisfaction	Poor methodological quality, high PICO heterogeneity	*“People with schizophrenia and their families should be willing to spend a significant amount of time in contact with services.”*
Piat et al. (2015) [62]	4/5 critical items not met	Eating disorder	International training programs	Natural supporters (parents or other family members, carers, friends)	10 studies on caregivers, of this 5 on adult patients, 3 RCTs, countries: Canada (3), UK (5), USA (7), Australia (3), Norway (2)	Not the focus of the review	Knowledge and related skills, distress, burden, coping, communication, family functioning	Heterogeneity of studies, divergent objectives of the training programs, wide array of methodologies, no quality rating of included studies, no effect sizes reported, no user outcomes	*“Results suggest that the supportive, face-to-face element of training for families and natural supporters, both between trainers and trainees and among trainees themselves, was highly beneficial. This implies that ED training using passive learning approaches may be less effective for families, for whom the lived experience of training was an added value.”*
Rane et al. (2017) [63]	3/5 critical items not met	Substance use disorder	Psychosocial interventions	Immediate family, other relatives and friends	4 studies (1 cluster-RCT) countries: Mexico (2), Vietnam, Malaysia	Symptom change, psychological symptoms (e.g. depression), drug use behaviour	Coping behaviour, assertiveness, self-esteem	Evidence base extremely sparse, studies either exploratory or pilot trials with small sample sizes, predominantly female participants, measurement of varied elements	*“The limited evidence does suggest positive benefits to AFMs. The scope of research needs to be extended to other addictions, and family members other than spouse and female relatives.”*
Reupert et a. (2013) [64]	2/3 critical items not met (limited applicability of AMSTAR2 (descriptive focus))	Miscellaneous (mental illness)	Miscellaneous programs (focussing on children’s wellbeing)	Children whose parents have a mental illness	not summarized (*n* = 27 incl. Grey literature?) countries: not reported	Not the focus of the review	Family dysfunction, children’s support networks, hyperactivity, anxiety, depression, stress, self-esteem, coping skills, connections within family, knowledge, mental health literacy, awareness, life satisfaction, problem-solving, stigma, social support, parent–child interaction …	Poor reporting, no synopsis on quantitative outcomes	*“The core component across programs is the provision of psychosocial education to children about mental illness. More rigorous research is required to establish the conditions through which children’s outcomes are enhanced.”*
Rodolico et al. (2022) [65]	All critical items met	Schizophrenia	Family intervention	Family/relatives	90 RCTs countries: China (30), USA (16), UK (12), Italy (6), Germany (5), Spain (4), Denmark (2), India (2), Japan (2), Turkey (2), Australia (2), Norway (1), Jordan (1), The Netherlands (1), Hong Kong (1), Indonesia (1), Poland (1), Colombia (1), Thailand (1), Malaysia	Relapse	Not summarized	Partly indirect evidence, partly low statistical power, many studies with high bias risk, wide range of relapse definitions, variability in the level of detail in the intervention descriptions in the study manuscripts	*“Almost all family intervention models were efficacious in preventing relapse in schizophrenia. Family psychoeducation alone, without behavioural or skills training, was superior to the more complex models. The simplest form of family psychoeducation was ranked among the most efficacious interventions”*
Rushton et al. (2023) [66]	2/7 critical items not met	Substance use disorder	Psychosocial interventions (focussing on caregiver wellbeing)	Concerned significant others, caregivers, partners, adult children and friends.	19 studies (11 RCTs) countries: USA, Germany, Spain, Canada, Iran, Sweden, Australia, Vietnam, India, UK	Not the focus of the review	Depression, distress, coping, family functioning	All studies rated as having either some concerns or high/serious risk of bias, insufficient description of methodology, small sample sizes, high drop-outs and limited long-term follow-ups.	*“Findings demonstrate favourable outcomes for families regardless of whether interventions are delivered to individuals or groups, particularly with respect to reducing families’ depression and distress. Although caution is warranted, these findings are promising given the considerable burden experienced by families and the historical omission of families within the evaluation literature.”*
Sin et al. (2017) [67]	1/7 critical items not met	Psychosis	Psychoeducational interventions (focussing on caregiver wellbeing)	Parents, siblings, sprouts, friends	32 RCTs countries: China (10), North America (4), Europe (4), UK (4) Middle East (6), South America (1), Australia (2), India (1)	Not the focus of the review	Wellbeing, stress, global morbidities, depression, perceived burden, negative caregiving experiences, expressed emotion	Very heterogenous scales for caregiver outcomes → low statistical power; sparse follow-up data	*“… how these carers’ outcomes correlate to patients’ outcomes like decreased relapse and better compliance, and to family-wide outcomes like family relationship and communication, and vice versa, needs to be better explored.”*
Spain et al. (2017) [67]	All critical items met	Autism	Family therapy	Family, significant others	No studies found	No studies found	No studies found	No studies found	*“Few studies have examined the effectiveness of family therapy for ASD, and none of these are RCTs. Further research studies employing methodologically robust trial designs are needed to establish whether family therapy interventions are clinically beneficial for enhancing communication, strengthening relationships, augmenting coping and reducing mental health morbidity for individuals with ASD and family members”*
Stahl et al. (2016) [68]	3/5 critical items not met	Depression (late-life)	Dyadic and family-oriented interventions	Dyads (siblings, spouses)	13 articles (10 RCTs) countries: USA (*n* = 8), Germany, Japan, Netherlands	Not summarized, reported for each study separately, depression symptoms	Only reported in 1/13 primary studies (support person’s depression)	Inclusion not consistently via depression as main diagnosis, but also e.g. via dementia etc, no comparison of dyadic with individual therapy	*“Remarkably few dyadic intervention studies have been attempted to treat depression in older adults. This review showed that dyadic interventions are feasible and that including support persons significantly decreased patients’ depressive symptomatology.”*
Stiawa et al. (2014) [69]	4/5 critical items not met	Miscellaneous (mental health problems)	Psychosocial interventions	Children	8 studies (7 RCTs) countries: USA (5), UK (1), Canada (1), Australia (1)	Social relationships outcomes: role function, social competence, self-confidence, social support, problem-solving, coping, parenting, parent-child relationship Medical outcomes: medical utilization, psychological symptoms, utilization of treatment, knowledge on help services, severity of depression or anxiety, quality of life	Problem behaviour, mental illness, anxiety symptoms	Highly heterogenous interventions and population, poor reporting	*The complex, often multidisciplinary support services, frequently with a psychosocial focus, which were offered to the participants of the interventions and which mainly provided them with information and a sense of security, should be available to an appropriate extent for people with (a) psychological symptoms and (b) a low socio-economic status in order to improve the situation of those affected with sufficient support in the long term.”*
Sutherland et al. (2020) [70]	3/5 critical items not met	Borderline	Miscellaneous (focussing on caregiver wellbeing)	Mostly parents, but also spouses/partners, siblings, (adult) children	11 studies on 7 different interventions (2 RCTs) countries: 6 USA, 2 Australia, 1 Ireland, 2 UK	Symptoms, self-harm, aggression, suicidal threats, fights, withdrawal	Burden, mental health, grief, coping, family functioning, expressed emotions, knowledge	Significant methodological limitations: lack of control groups, limited follow-up, high dropout rates, incomplete data sets, studies highly heterogeneous, wide variety of outcome measures, poor reporting of effect sizes	*“Group interventions for carers may lead to some improvements in carer well-being and reductions in carer burden and grief.”*
Sutherland et al. (2023) [71]	4/5 critical items not met	Autism or associated genetic syndrome, intellectual disability (ID)	Family-systems interventions	Family members of a person with the named disorders, included were biological, adoptive, foster or stepfamily members	11 studies (2 RCTs) countries: UK (*n* = 4), Hong Kong (*n* = 4), the Netherlands (*n* = 2), the USA (*n* = 2), Spain (*n* = 1)	Wellbeing, rule breaking behaviour living at home, symptom reduction (such as reduction in behaviour that challenges)	Wellbeing, family relationships family adaptability, family functioning, caregiver strain self-esteem	Few RCTs, small samples, lack of control groups and methodological weaknesses	*“There is a need for higher-quality research to establish whether family-systems interventions are beneficial for families of people who have an ID or who are autistic.”*
Thompson et al. (2017) [72]	2/5 critical items not met	Hoarding disorder	Psychological, pharmacological, and family-based interventions	Not specified	2 studies (uncontrolled) countries: not reported	Family-rated symptoms	Coping, knowledge, anxiety, burden, wellbeing	Few studies on caregivers, no comparative studies (pre-post only), high attrition	*“This review also included two papers looking at interventions for relatives of people with HD. Both studies reviewed here had small sample sizes but showed promising results in reducing the impact on the family and improving understanding of HD.”*
Thompson-Hollands et al. (2014) [73]	4/5 critical items not met	Obsessive compulsive disorder	Miscellaneous “Family involvement in psychological treatment”	Family	29 studies, of this 7 on adults countries: not reported	Symptoms, functioning	Not reported	Most studies on non-adult users, heterogeneity across FITs, as well as the small number of trials, few comparative studies, no quality assessment	*“Results indicate a robust overall response to FITs for OCD and clarify key moderators that inform optimal circumstances for effective treatment.”*
van Es et al. (2023) [74]	1/7 critical items not met	Miscellaneous (not only mental disorders)	Multiple family therapy	Family (55% parents of children < 18)	31 studies (19 RCTs), 16 in meta-analysis countries: United States (52%), Western Europe (19%), China/Hong Kong (16%), Australia (6%)	positive and/or negative psychotic symptoms, for the outcome “mood problems” it is not clear if it refers to patients or to caregivers/families	Family functioning, for the outcome “mood problems” it is not clear if it refers to patients or to caregivers/families	Focus on MFT, resulting in an extremely heterogeneous population: not only mental disorders, 55% on children, all PICO categories very heterogeneous, for meta-analyses only few studies, high statistical heterogeneity	*“To conclude, more methodologically rigorous research is needed to further examine the potential benefits of MFT, as well as the working mechanisms and core components of MFT.”*
Wang et al. (2021) [57]	4/7 critical items not met	Miscellaneous (“serious mental Illness”)	Family- and individual-led peer support	Family	5 RCTs Countries: 4x Hong Kong, 1x USA	Psychosocial functioning, psychiatric symptoms, rehospitalization, duration of rehospitalization	Family function, burden, use of formal community support services	Elements of peer support for interventions differ, scales used for the same aspects are inconsistent, high statistical heterogeneity, follow-up time was inconsistent, different intervention locations (in- and outpatient)	*“These results suggest that peer support, as a potential resource, can be further developed for the rehabilitation of those with SMI, despite limited available evidence.”*
Zinser et al. (2022) [75]	1/7 critical items not met	Eating disorder	Multi-family therapy	Family	15 studies (2 RCTs) countries: 4xUK, 3xUSA, 2xCanada, 2xBelgium, 1xSweden, 1xDenmark, 1xCzech Republic, 1xNorway	Eating disorder symptoms, change in weight, depression, psychological wellbeing	Family functioning, negative caregiving appraisals, positive caregiving experiences	Wide-ranging variation in the MFT model, the length and structure of interventions, follow-up periods, and outcome measures, study quality weak to moderate, many studies without comparison group, no blinding, possible selection bias	*“Significant improvements were only evident when comparisons were drawn before and after the intervention; these dissipated when MFT was compared to another intervention. There was no evidence MFT improves family functioning, positive aspects of caregiving, nor patient and parental anxiety.”*

### Characteristics of the ES

Table 1 provides an overview of ES characteristics (for details: S6). The ES were extremely heterogenous regarding the population included, the interventions/ programs assessed, and the outcomes chosen, severely impeding sufficient comparability.

*NOK characteristics:* The notions and framings regarding NOK used in the ES selected were widely heterogenous (see Box 1). NOK characteristics were reported insufficiently in many ES, some stating poor reporting within the primary studies too [75, 76] (see S6). For instance, the kinship position was rarely specified or only referred to in rather unspecific terms, only some ES explicitly including non-family NOK. Only some ES reported the age and gender of NOK, a few reporting the absence of such information in primary studies. At times, it was not clear whether the data referred to participating PIT or NOK, or both. Inferred on this incomplete basis, most NOK included were female [32, 63, 75].

**Box 1:** NOK terms und synonyms used in the literature screened and selected

**Comprehensive NOK terms** • (informal) caregiver • carer • caring (i.e. persons, relatives, friends, family members) • informal support persons • significant others • natural supporters •close relationship **Non-family NOK terms** • friends • neighbours	**Family NOK terms** • family • relatives (sometimes defined precisely: 1^st^ or 2^nd^ degree, immediate, biological, adoptive, foster, stepfamily members) • family relatives • children • sprouts • parents • siblings • spouses • partners • dyads

*PIT characteristics*: Most ES focused on PIT diagnosed with psychotic disorders (7 publications) and schizophrenia [6]. A total of 19 ES was diagnosis-unspecific, of which 16 focused on various diagnoses, and three ES addressed suicidal behavior.

*Intervention characteristics*: Table 2 summarizes some intervention details. Often, there was a lack of clearly named intervention, program, or manual. If described, one intervention was barely assessed in more than one ES. Interventions were extremely heterogenous in their parameters (e.g. theoretical underpinnings, components/ modules used, setting, etc.). The most common component was psychoeducation, involving a wide spectrum of strategies and recipients (e.g. groups/ individuals/ dyads; with/ without PIT; lectures/ workshops; delivered by various professions, in clinical/ community settings). Other frequent components were counselling, communication and skills training, and support groups. The ES reported huge variations in implementation (see Table 2) even in identical manuals.

**Table 2:** Details of interventions

Parameters	Examples	Comments & Questions
Duration of Intervention	Singular, over a few weeks or months to several years	- often not reported/ poor reporting of details - usually derived from the table of study characteristics - distinctions between the duration of the study and the duration of the practice intervention - often only post-intervention data and no follow-up
Intensity	One or more times per week/a month, or as needed	- often not reported / poor reporting on details - usually derived from the table of study characteristics
Recipients	NOK only (one or more persons), NOK + PIT; individuals, partly together, partly separately, couples/dyads, families and other networks, multi-family	- often not reported / poor reporting on details - usually derived from the table of study characteristics
Setting	Inpatient (hospital, forensic institution), outpatient/community setting/ outreach, other settings (i.e. school, campsite …)	- often not reported / poor reporting on details - if reported, the setting plays an important role
Delivering staff	Professionals staff (clinicians, various types of therapists), nursing staff, social workers, counsellors, peer support workers; various combinations of the above	- often not reported / poor reporting on details - if reported, the type of staff is important, for instance in relation to its feasibility and cost-effectiveness etc.
Delivery format	Sessions, lectures, workshops, internet programs, internet forum bibliotherapy, workbooks, diaries, homework, toolkits, leisure time activities (sports, excursions), creative activities, phone calls, home visits, day care services, mutual support groups (with/without guidance); various combinations of the above	- often not reported/ poor reporting on details - usually derived from the table of study characteristics - often no clear distinction between components, delivery format and theoretical underpinnings - hardly any ES on internet/mobile-based interventions, despite the wealth or primary studies
Concepts, theoretical underpinnings, philosophy, rationale	Behavioural/cognitive/dialectic behavioural theory principles, systemic principles, psychodynamic psychotherapy, acceptance commitment therapy, emotion-focused therapy, trauma-focused therapy, mindfulness-based approaches, recovery	- often not reported/ poor reporting on details - often no clear distinction between components, delivery format and theoretical underpinnings
Components, strategies, techniques	Assessment of needs, exploration of experiences, educational elements, psychological/psychotherapeutic elements (counselling, recovery coaching, emotional coaching, skill training, problem-solving, communication training, stress management, motivational interviewing, relaxation training, drama therapy, positive psychotherapy, emotional regulation training, progressive muscle relaxation, deep breathing, transactional analysis, mentalization-based therapy, relational models, exposure-based treatment, discharge planning, general support, provision of information, referring to specialized supportpeer/partnerships, pharmacotherapy, self-help with/without support; various combinations of the above	- often not reported / poor reporting on details - usually derived from the table of study characteristics - often no clear distinction between components, delivery format and theoretical underpinnings
Manualized interventions, concrete programs, models	Family psychoeducation models, (single) family therapy (i.e. Falloon-Manual], multi family therapy (MFT), behavioral family psychoeducational program (BFPEP), behavioral couple/family therapy (BFT), structural family therapy (SFT), family system approaches, multi systemic therapy (MST), system therapy methods in acute psychiatry (SYMPA), open dialogue, somerset model, family system approach, systemic behavioral family therapy (SBFT), ecosystem focused therapy (EFT), systemic autism-related family enabling (SAFE), attachment-based family therapy (ABFT), reconnecting for recovery (R4R); Calgary family assessment and intervention models; family bereavement program, lifespan emotion focused family therapy model, unilateral family interventions …	- often not reported / poor reporting on details - In many cases, only one study on each intervention - If interventions/programs have been investigated in more than one study, there are usually variations in their implementation often no clear distinction to theoretical underpinnings

*Clustering attempts*: ES differently categorized NOK involvement (by participants, the intervention setting, delivering staff etc.) [56, 61, 64, 65, 77, 78]. To present some examples, Frey et al. separate cognitive and dialectic behavioral therapy-based approaches from systemic and/ or psychoeducational strategies [43]. Rodolico et al. differentiate between systemic-oriented family, community interventions, psychoeducational, and family approaches [65], whereas the latter are further separated according to components and strategies. Dirik et al. identify six family involvement models in acute mental healthcare, mapping them alongside underlying theories [77].

*Outcomes assessed:* Four ES did not report quantitative outcomes [16, 42, 77, 79]. Of the 43 ES with quantitative outcome reporting, 26 reported both NOK and PIT outcomes. Reporting on dropouts was poor, with some exceptions [72]. Some authors [63, 80] stated that there was a lack of information on attrition in primary studies. For an overview of used outcomes, see Table 3.

**Table 3:** Frequently assessed patient and NOK outcomes

Person in Treatment Outcomes	
Disorder-related	Symptoms (disorder-specific measures), relapse, suicidal ideation/attempts, self-harm behaviour
Skills	Knowledge, self-care, social competence, functioning
Treatment-related	Adherence/ compliance, service utilization, hospitalization, treatment initiation, treatment withdrawal
**Next of Kin Outcomes**	
Caregiving	Burden, distress/grief, experiences, negative/positive aspects of caregiving, needs
Health	General wellbeing, psychological wellbeing/mental health, depression, anxiety, quality of life, burnout
Skills	Knowledge, mental health literacy, coping, communication, self-esteem, empowerment, problem-solving, behavioural change, assertiveness
Support	Help-seeking, social support, service utilization, service satisfaction
Patient-related	Stigma, guilt, attitude, expressed emotion, empathy, tolerance, emotional involvement and criticism
System-related	Family functioning, family relationship, relationship satisfaction, stability

*NOK outcomes*: Quantitative NOK outcomes were reported in 40 studies, whereas the reporting quality was poor. Instead of summarizing NOK outcomes, they were reported for each study separately. Or it was impossible to deduce whether outcomes were measured for NOK or for PIT only [56, 74]. This lack of quality often was due to low reporting on the level of primary studies [51, 65, 68]. In three ES, NOK outcomes were not mentioned at all [73, 81]. The most common NOK outcomes were *burden*, *stress*, *quality of life* and *wellbeing*, *coping skills* and *disease knowledge* (see Table 3). At family level, typical outcomes were *family functioning* and *expressed emotions*. A variety of scales were used. The most frequent NOK measures were *Family Assessment Device* (FAD), *Family Burden Interview Scale* (FBIS), and *Family Support Services Index* (FSSI).

*PIT outcomes*: PIT outcomes were reported in only 29 of 43 studies, the remaining 14 ES focused on NOK outcomes only [31, 45, 48, 49, 52, 53, 57, 58, 62, 64, 66, 67, 82, 83]. The most common outcomes at the level of PIT were *relapse rates*, *service utilization*, *symptom severity* and *functionality* as well as *quality of life* or *general wellbeing*.

### Inferences on effectiveness

Certainty of evidence was low, due to heterogeneity (of participants, interventions, controls, outcome measures, and results), risk of bias, and imprecision (few studies and participants, wide confidence intervals). Only 16 of the ES conducted a meta-analysis, with only 9 analysing the effects on NOK, with different quality, making any generalization difficult. The effects of five ES with a good reporting quality are presented in S8, suggesting that interventions for the involvement of NOK may result in moderate to large effect sizes in both NOK and PIT outcomes [45, 66, 67, 79, 83]. The most certain effects were related to psychoeducation.

## Discussion

To our knowledge, this is the first participatory overview of structured interventions for NOK involvement in mental health care. First, we found far more evidence than expected. The included ES reported on a variety of approaches, which is reflected in the heterogeneity of the interventions, their conceptual backgrounds, components, duration, intensity, and delivering staff, but also in the different terms and outcomes used for NOK – a heterogeneity that made it impossible for us to systematically compare and summarize the evidence.

### Types of kinship positions and mental disorders

As stated above, the ES under review used a broad range of terms to designate kinship relationships. Most terms were employed without providing any conceptual background. Only a minority of ES explicitly defined the terms they used, making it necessary to first compile a broad range of synonyms (see Box 1) – a major obstacle to the identification and synthesis of evidence.

Besides this lack of clarity, the NOK sociodemographic characteristics were reported insufficiently, at times leading to confusion, to whom the reported data referred to. Inferred on this incomplete basis, most NOK involved were female, reflecting the unequal distribution of burden of care [84]. Further, most ES reported on the involvement of (core) family members only, whereas non-family NOK were either excluded or not specified – a finding that is unsatisfactory given the rapid diversification of types of kinship [85].

Further, only a few primary studies recruited NOK independently from PIT. Such recruitment logic may indicate that NOK are insufficiently perceived as an intervention group on their own. This secondary status of NOK is reproduced at the level of interventions and outcomes (see below) and has been criticized in relation to clinical practices too [86], for instance through the individuum-centrism of usual mental health care models [87, 88].

Accordingly, most ES focus on PIT diagnoses as primary inclusion criteria and less on the relationships between PIT and NOK. Among them, schizophrenia-spectrum disorders were at the center, which may shed light on the historical developments of NOK-involvement, and especially by the wide spectrum of stereotypes on the NOK (co-)causation of these disorders [65]. Another explanation could be that in care work, psychotic behavior may trigger more misunderstandings and feelings of estrangement in comparison to other disorders [55], making NOK involvement seem more urgent [89].

### Types of interventions

There was a lack of clearly defined interventions. If existing, definitions were inconsistent and heterogenous regarding their theoretical underpinnings, components, strategies, settings, delivery formats, duration and frequency. Even in case of manualization, it often was unclear what “involvement” meant in detail. One intervention was barely assessed in more than one ES, making their comparability across studies difficult.

These findings demonstrate that more program theory is needed to prepare the ground for the sustainable implementation of NOK involvement. Further, interventions that offered process-orientated support in multi-person settings, such as the Open Dialogue approach [90] were underrepresented. This may be explained by their lack of fit to the individuum-centrism of usual mental health care models too, being also reflected in the usual accounting systems that mostly allow remuneration for services to support PIT only [91, 92].

### Types of outcomes and estimation of effects

PIT outcomes were largely clinically oriented, whereas most of NOK outcomes targeted well-being. This raised questions in relation to the commensurability of outcomes across these groups of participants. Thus, it was striking that most ES examined either PIT or NOK outcomes separately, without comparing them, neglecting the fact that both types usually are related [93]: people stand in relationship, making analytic approaches necessary that address the various interdependencies between NOK and PIT data to understand their reciprocal effects as part of an intervention’s context [94–96].

Further, the extreme heterogeneity of both the NOK and PIT outcomes made any systematic comparison across studies impossible. However, estimated effects were positive on both PIT and NOK (see S8), so that it seems sensible to implement structured programs for NOK involvement in mental health care. Yet, it is impossible to argue which types of interventions, therapeutic components, settings and organizational structures contribute to which effects in relation to which mental disorder or kinship position. More nuanced recommendations can only be presented if the goals and mediators of the interventions existing are more refined as well as the roles and functions that NOK fulfill in this context.

### Limitations

This overview has several limitations that should be considered when interpreting the findings. First, as an umbrella review, it represents a tertiary level of evidence and is therefore dependent on the scope, quality, and reporting of the included ES. Missing information may reflect either inadequate reporting in the primary studies or incomplete extraction and synthesis at the review level. Consequently, some intervention characteristics, participant details, and outcomes may have been underrepresented in our analysis. Second, we did not systematically extract or analyse data from the primary studies included in the ES. While this approach is consistent with the methodology of umbrella reviews, it limited our ability to clarify inconsistencies across reviews, verify reported findings, examine intervention components in greater detail, and identify context-specific factors associated with effectiveness. Third, overlap of primary studies across the ES was not systematically assessed. Although our exploratory analysis of reviews on schizophrenia-spectrum disorders suggested a relatively low degree of overlap, duplicate inclusion of primary studies across reviews cannot be ruled out. Fourth, despite employing a resource-efficient search strategy that combined major review databases with reference checking and an additional search in PubPsych, we cannot exclude the possibility that relevant ES were missed. Furthermore, only reviews published in databases accessible through our search strategy were considered. Fifth, due to resource constraints, only a subset of the AMSTAR-2 assessments was conducted independently by two reviewers. Although consensus procedures were applied and a common assessment approach was established, some degree of subjectivity in quality ratings cannot be excluded. Sixth, the substantial heterogeneity of populations, kinship relationships, interventions, outcomes, and review methodologies precluded quantitative synthesis at the overview level. Consequently, no meta-analysis was conducted, and the findings are based on narrative and interpretative synthesis. Finally, the broad conceptualization of NOK and the intentionally inclusive search strategy may have increased heterogeneity and reduced comparability between reviews. However, a narrower focus would likely have excluded important forms of kinship and participation and would therefore have been inconsistent with the aim of providing a comprehensive overview of the field.

## Conclusions

It is to conclude that the individuum-centrism of current mental health care services represents a substantial obstacle towards the systematic implementation of NOK involvement. To ensure the feasibility and sustainability of NOK involvement, we need a mental health care system that remunerates multi-person interventions, allowing for the symmetric participation of both NOK and PIT. The best intervention is useless if there is no sustainable economic basis for its lasting implementation.

Further, the multitude of heterogeneous interventions reflects the growing acknowledgement of the importance of NOK in the field of mental health care. For a systematic comparison, it is necessary to address the interdependency of effects between PIT and NOK outcomes. Further, future studies should report in more detail their rationale, components and mediators to create a theoretical basis that allows for the interventions’ sustainable implementation [97, 98].

## Electronic supplementary material

Below is the link to the electronic supplementary material.

Supplementary Material 1

## Data Availability

All available data has been submitted in the manuscript or as supplemental materials.
