# Supplementary material for: Next of kin involvement in mental health care services – a systematic participatory overview of evidence syntheses
Source: BMC Psychiatry. 2026 Jun 30;26:496. doi: 10.1186/s12888-026-08313-5 (PMC13321765; doi:10.1186/s12888-026-08313-5)
Supplement: Supplementary file 1 — Supplementary Material 1 [file 12888_2026_8313_MOESM1_ESM.docx]

**Supplemental Appendices**

[1 Search Strategy 2](#_Toc232154340)

[2 Inclusion and exclusion criteria 4](#_Toc232154341)

[3 Excluded studies after full text screening 5](#_Toc232154342)

[4 AMSTAR2 assessment 1](#_Toc232154343)

[5 Extraction form 1](#_Toc232154344)

[6 Characteristics of includes studies 3](#_Toc232154345)

[7 Matrix of included primary studies in evidence syntheses on schizophrenia 78](#_Toc232154346)

[8 Effectiveness of a selection of next of kin interventions 83](#_Toc232154347)

1. [Effects on next of kin according to the ES with meta-analyses on next of kin outcomes 85](#_Toc232154348)

# S1 Search Strategy

## Medline via Pubmed

### cluster disorders

((mental[Title] OR psych*[Title] OR schizo*[Title] OR affective[Title] OR mood[Title] OR personality[Title] OR dissociativ*[Title]) AND (crisis[Title] OR crises[Title] OR health[Title] OR healthcare[Title] OR problem*[Title] OR disease*[Title] OR diagnos*[Title] OR illness*[Title] OR behaviour*[Title])) OR disorder*[Title] OR (mad[Title] OR madness[Title] OR depress*[Title] OR bipolar[Title] OR somatoform[Title] OR borderline[Title] OR autism*[Title] OR psychoses[Title] OR psychosis[Title] OR psychotic[Title] OR schizo*[Title] OR addict*[ Title] OR „substance abuse“[Title] OR „drug abuse“[Title] OR „substance use” [Title])

### cluster next of kin

(caregiv*[Title] OR “informal help*”[Title] OR “peer help*” [Title] OR famil*[Title] OR friend*[Title] OR neighbo*[Title] OR relatives[Title] OR carer*[Title] OR "lay person"[Title] OR kinship[Title] OR kinsmen[Title] OR "next of kin"[Title] OR Associate*[Title] OR "social network"[Title] OR "social environment"[Title] OR "Social milieu"[Title] OR Peer*[Title] OR guardian*[Title] OR "reference person*"[Title] OR collegue*[Title] OR Spouse*[Title] OR Partner*[Title] OR Sibling*[Title] OR Husband*[Title] OR Wife*[Title] OR Parent*[Title] OR "adult children"[Title] OR Intrafamily*[Title])
18.09.: 1,194,694

### cluster participation

(participat*[Title] OR collaborat*[Title] OR Involve*[Title] OR Integrat*[Title] OR engage*[Title] OR inclus*[Title] OR responsib*[Title])
20231002:

### cluster interventions

program[Title] OR programme[Title] OR intervention[Title] OR „Open dialog*"[Title] OR Trialog*[Title] OR “Home treatment”[Title] OR „Peer support"[Title] OR „peer intervent*"[Title] OR „Family *therapy"[Title] OR “Integrated care”[Title] OR “Collaborative care”[Title] OR "Social intervention*"[Title] OR psychosocial[Title] OR "social support"[Title] OR training[Title] OR treatment[Title] OR counselling[Title] OR “health service*”[Title] OR “community mental health” [Title] OR “community health”[Title] OR recovery[Title] OR help[Title]

### systematic reviews

VonVille2023

<https://www.yopl.info/post/pubmed-research-methodology-search-filters-and-a-couple-of-nifty-limits>

### realist reviews

(“Realist* review”[TIAB] OR “realist* approach”[TIAB] OR “realist* synthesis”[TIAB] OR “realist* research”[TIAB] OR “realist* evaluation”[TIAB])

## Epistemonikos

title:(((mental OR psych* OR schizo* OR affective OR mood OR personality OR dissociativ*) AND (crisis OR crises OR health OR healthcare OR problem* OR disease* OR diagnos* OR illness* OR behaviour*)) OR disorder* OR (mad OR madness OR depress* OR bipolar OR somatoform OR borderline OR autism* OR psychoses OR psychosis OR psychotic OR schizo* OR addict* OR "substance abuse" OR "drug abuse" OR "substance use")) AND title:((caregiv* OR "informal help*" OR "peer help*" OR famil* OR friend* OR neighbo* OR relatives OR carer* OR "lay person" OR kinship OR kinsmen OR "next of kin" OR Associate* OR "social network" OR "social environment" OR "Social milieu" OR Peer* OR guardian* OR "reference person*" OR collegue* OR Spouse* OR Partner* OR Sibling* OR Husband* OR Wife* OR Parent* OR "adult children" OR Intrafamily*)) AND title:((participat* OR collaborat* OR Involve* OR integrat* OR engage* OR inclus* OR responsib* OR program OR programme OR intervention OR "Open dialog*" OR Trialog* OR Home treatment OR "Peer support" OR "peer intervent*" OR "Family therapy" OR Integrated care OR Collaborative care OR "Social intervention" OR psychosocial OR "social support" OR training OR treatment OR counselling OR "health service*" OR "community mental health" OR "community health" OR recovery OR help))

## PubPsych

TI=(caregiv* OR "informal OR help*" OR "peer OR help*" OR famil* OR carer* OR "social OR network" OR "social OR environment" OR "Social OR milieu" OR Peer* OR intrafamily*)

# S2 Inclusion and exclusion criteria of the literatur search

|  | Inclusion | Exclusion |
| --- | --- | --- |
| P | - mental health; - miscellaneous diseases with a relevant proportion of included studies on mental health; - studies with patients ≥18 years or on studies with a relevant proportion of included studies on patients ≥18 | - not on mental health (incl. dementia); - reviews on miscellaneous diseases without a relevant proportion of included studies on mental health; - reviews on patients <18 years |
|  | - next of kin or family members regardless of their age | - no next of kin/family involvement; - professional carers; - parents of children <18 years |
| I | - concrete interventions, programs, or structured approaches for next of kin involvement or family-oriented therapies - interventions on next of kin wellbeing | - studies on next of kin experiences and needs (perspectives, barriers, facilitators, burden …) without connection to a concrete intervention, program, or structured approach for next of kin involvement or family-oriented therapy |
| C | - not restricted |  |
| O | - not restricted |  |
| publication type | - systematic reviews, scoping reviews, realist reviews, qualitative evidence syntheses (at least a systematic search must have been conducted) | - all other publication types |
| language | - english, german | - all others |

# S3 Excluded studies after full text screening

| Abou Seif N,Wood L,Morant N,N AS,Wood L,Morant N | Invisible experts: a systematic review & thematic synthesis of informal carer experiences of inpatient mental health care | 2022 | primary studies are not on specific next of kin interventions/programs |
| --- | --- | --- | --- |
| Albano G,Hodsoll J,Kan C,Lo Coco G,Cardi V | Task-sharing interventions for patients with anorexia nervosa or their carers: a systematic evaluation of the literature and meta-analysis of outcomes | 2019 | not on adult patients |
| Bentsen H | [Does psychoeducational family intervention improve outcome of schizophrenia?] | 2003 | not in english or german |
| Bird V,Premkumar P,Kendall T,Whittington C,Mitchell J,Kuipers E | Early intervention services, cognitive-behavioural therapy and family intervention in early psychosis: systematic review | 2010 | not up-to-date/redundant |
| Cameron SL,Tchernegovski P,Maybery D | Mental health service users' experiences and perspectives of family involvement in their care: a systematic literature review | 2022 | primary studies are not on specific next of kin interventions/programs |
| Campbell LA,Clark SE,Chorney J,Emberly D,MacDonald J,MacKenzie A,Warner G,Wozney L | Choice and Partnership Approach to community mental health and addiction services: a realist-informed scoping review | 2022 | not on adult patients |
| Cook-Darzens S | The role of family meals in the treatment of eating disorders: a scoping review of the literature and implications | 2016 | no systematic review; focus on meals/exclusion of systemic approaches |
| De Boer K,Muir SD,Silva SS,Nedeljkovic M,Seabrook E,Thomas N,Meyer D | Videoconferencing psychotherapy for couples and families: A systematic review | 2021 | not focussing on psychiatric disorders |
| Doody O,Butler MP,Lyons R,Newman D | Families' experiences of involvement in care planning in mental health services: an integrative literature review | 2017 | primary studies are not on specific next of kin interventions/programs |
| Droogan J,Bannigan K | A review of psychosocial family interventions for schizophrenia | 1997 | not up-to-date/redundant |
| Epps F,To H,Liu TT,Karanjit A,Warren G | Effect of Exercise Training on the Mental and Physical Well-Being of Caregivers for Persons Living With Chronic Illnesses: A Systematic Review and Meta-Analysis | 2019 | not focussing on psychiatric disorders |
| Factor RS,Ollendick TH,Cooper LD,Dunsmore JC,Rea HM,Scarpa A | All in the Family: A Systematic Review of the Effect of Caregiver-Administered Autism Spectrum Disorder Interventions on Family Functioning and Relationships | 2019 | not on adult patients |
| Filges T,Andersen D,JÃ¸rgensen AM | Effects of multidimensional family therapy (MDFT) on nonopioid drug abuse: A systematic review and meta-analysis | 2015 | not on adult patients |
| Gil M,SimÃµes MM,de Oliveira-Cardoso Ã‰A,Pessa RP,Leonidas C,dos Santos MA | Perception of family members of people with eating disorders about treatment: a metasynthesis of the literature, | 2022 | primary studies are not on specific next of kin interventions/programs |
| Goger P,Weersing VR | Family based treatment of anxiety disorders: A review of the literature (2010-2019) | 2021 | not on adult patients |
| Goorden M,Schawo SJ,Bouwmans-Frijters CA,van der Schee E,Hendriks VM,Hakkaart-van Roijen L,E S,Hendriks VM,L HR | The cost-effectiveness of family/family-based therapy for treatment of externalizing disorders, substance use disorders and delinquency: a systematic review | 2016 | not focussing on psychiatric disorders; not on adult patients |
| Gregg L,Adderley H,Calam R,Wittkowski A | The implementation of family-focused practice in adult mental health services: A systematic review exploring the influence of practitioner and workplace factors | 2021 | primary studies are not on specific next of kin interventions/programs |
| Kurnik MesariÄ K,Damjanac Å½,Debeljak T,KodriÄ J | Effectiveness of psychoeducation for children, adolescents and caregivers in the treatment of eating disorders: A systematic review | 2023 | not on adult patients |
| Lambert S,Brahim LO,McCusker J,Coumoundouros C,Audet LA,Yaffe M,Kayser J,Magalhaes M,Belzile E,Turner N | Non-pharmacological Interventions for Caregivers with Depression and Caregivers of Care Recipients with Co-morbid Depression: Systematic Review and Meta-analysis | 2021 | not focussing on psychiatric disorders |
| Lambert SD,Duncan LR,Kapellas S,Bruson AM,Myrand M,D SM,Culos-Reed N,Lambrou A | A Descriptive Systematic Review of Physical Activity Interventions for Caregivers: Effects on Caregivers' and Care Recipients' Psychosocial Outcomes, Physical Activity Levels, and Physical Health | 2016 | not focussing on psychiatric disorders |
| Lamont E,Dickens GL | Mental health services, care provision, and professional support for people diagnosed with borderline personality disorder: systematic review of service-user, family, and carer perspectives | 2019 | not focussing on next of kins |
| Landeweer E,Molewijk B,Hem MH,Pedersen R | Worlds apart? A scoping review addressing different stakeholder perspectives on barriers to family involvement in the care for persons with severe mental illness | 2017 | primary studies are not on specific next of kin interventions/programs |
| Lawrie SM | Review: family intervention added to standard treatment for schizophrenia relapse | 2001 | not up-to-date/redundant |
| Li G,Yuan H,Zhang W | The Effects of Mindfulness-Based Stress Reduction for Family Caregivers: Systematic Review | 2016 | not focussing on psychiatric disorders |
| Ma CF,Chan SK,Chien WT,Bressington D,Mui EY,Lee EH,Chen EY | Cognitive behavioural family intervention for people diagnosed with severe mental illness and their families: A systematic review and meta-analysis of randomized controlled trials | 2019 | doublet |
| Martin RM,Ridley SC,Gillieatt SJ | Family inclusion in mental health services: Reality or rhetoric? | 2017 | primary studies are not on specific next of kin interventions/programs |
| McCarthy MJ,Sanchez A,Garcia YE,Bakas T | A systematic review of psychosocial interventions for Latinx and American Indian patient-family caregiver dyads coping with chronic health conditions | 2021 | not focussing on psychiatric disorders |
| Meis L,Griffin J,Greer N,Jensen A,Carlyle M,MacDonald R,Rutks I,Wilt TJ | Family Involved Psychosocial Treatments for Adult Mental Health Conditions: A Review of the Evidence | 2012 | doublet |
| Pharoah F,Mari J,Rathbone J,Wong W | Family intervention for schizophrenia | 2006 | not up-to-date/redundant |
| Pharoah FM,Mari JJ,Streiner D | Family intervention for schizophrenia | 2000 | not up-to-date/redundant |
| Pharoah FM,Rathbone J,Mari JJ,Streiner D | Family intervention for schizophrenia | 2003 | not up-to-date/redundant |
| Pilling S,Bebbington P,Kuipers E,Garety P,Geddes J,Orbach G,Morgan C | Psychological treatments in schizophrenia: I. Meta-analysis of family intervention and cognitive behaviour therapy | 2002 | not up-to-date/redundant |
| Rodrigues MG,Krauss-Silva L,Martins AC | Meta-analysis of clinical trials on family intervention in schizophrenia | 2008 | not in english or german |
| Schlegl S,Voderholzer U,Maier J,Naab S,Lock J | Wirksamkeit, Moderatoren und Mediatoren manualisierter familienbasierter Therapie bei Jugendlichen mit Essstï¿½rungen: Eine systematische ï¿½bersichtsarbeit | 2020 | not on adult patients |
| Scott R,Aboud A | Engagement of mental health service users and carers in care planning - Is it meaningful and adding value? | 2021 | no systematic review |
| Semrau M,Lempp H,Keynejad R,Evans-Lacko S,Mugisha J,Raja S,Lamichhane J,Alem A,Thornicroft G,Hanlon C | Service user and caregiver involvement in mental health system strengthening in low- and middle-income countries: systematic review | 2016 | primary studies are not on specific next of kin interventions/programs |
| Sin J,Norman I | Psychoeducational interventions for family members of people with schizophrenia: a mixed-method systematic review | 2013 | not up-to-date/redundant |
| Stanton MD,Shadish WR | Outcome, attrition, and family-couples treatment for drug abuse: a meta-analysis and review of the controlled, comparative studies | 1997 | not up-to-date/redundant |
| Tuck M,Wittkowski A,Gregg L | A Balancing Act: A Systematic Review and Metasynthesis of Family-Focused Practice in Adult Mental Health Services | 2023 | primary studies are not on specific next of kin interventions/programs |
| Vasava T,Majhi G,Muralidhar D,Kumar D | Factors Associated with Family Retention or Involvement in Treatment of Persons with Severe Mental Illness: A Scoping Review | 2023 | primary studies are not on specific next of kin interventions/programs |
| Wells J,Kennedy C,Bain H,Lee SH | The experiences of older adults with a diagnosed functional mental illness, their carers and healthcare professionals in relation to mental health service delivery: An integrative review | 2019 | primary studies are not on specific next of kin interventions/programs |
| Zabihi S,Lemmel FK,Orgeta V | Behavioural Activation for Depression in Informal Caregivers: A Systematic Review and Meta-Analysis of Randomised Controlled Clinical Trials | 2020 | not focussing on psychiatric disorders |
| Downs KJ,Blow AJ | A substantive and methodological review of family-based treatment for eating disorders: the last 25 years of research | 2013 | no systematic review |

# S4 AMSTAR2 assessment criteria applied to the studies selected

| **source** | **# critical domains fulfilled** | **item 1** | **item 2** | **item 3** | **item 4** | **item 5** | **item 6** | **item 7** | **item 8** | **item 9** | **item 10** | **item 11** | **item 12** | **item 13** | **item 14** | **item 15** | **item 16** |
| --- | --- | --- | --- | --- | --- | --- | --- | --- | --- | --- | --- | --- | --- | --- | --- | --- | --- |
| Al-Sawafi (2020) | 4/5 | y | y | n | py | y | y | n | py | y | n | no MA | no MA | y | y | no MA | y |
| Ashcroft et al. (2018) | 2/7 | y | n | n | py | n | n | n | n (quite rudimentary table) | n | n | y | n | n | y | n | y |
| Bademli et al. (2011) | 1/5 | y | n | n | py | n | n | n | py | n | n | no MA | no MA | n | n | no MA | n |
| Barbeito et al. (2020) | 2/5 | y | n | n | py | y | n | n | y | n | n | no MA | no MA | y (with good will) | y | no MA | y |
| Baruch et al. (2018) | 5/7 | y | py | n | py | y | y | n | py | y | y | y | y (sensitivity analyses) | y | y | n | y |
| Baudinet et al. (2021) | 1/3 | y | n | n | py | y | y | n | py | Not required for scoping reviews | n | no MA | no MA | not applicable | n | no MA | y |
| Brady et al. (2017) | 3/5 | y | n | n | py | y | n | n | py | y | n | no MA | no MA | y | y | no MA | n |
| Camacho-Gomez (2019) | 5/7 | y | n | n | py | y | y | y (suppl) | py | y | n | y | y | n | y | y | y |
| Davies et al. (2021) | 3/5 | y | n | n | py | n | n | n | py | y | n | no MA | no MA | y (with good will) | y | no MA | y |
| Dehbozorgi et al. (2023) | 1/5 | n | n | n | py | n | n | n | n (quite rudimentary table) | n | n | no MA | no MA | n | y | no MA | y |
| Dirik et al. (2017) | 3/3 | y | y | n | py | n | n | n | n | not applicable | n | not applicable | not applicable | not applicable | not applicable | not applicable | y |
| Eassom et al. (2014) | 2/3 | y | py | n | py | y | y | n | y (supplement) | not applicable | n | no MA | no MA | not applicable | not applicable | no MA | y |
| Esteban et al. (2022) | 1/5 | y | n | n | py | n | n | n | y | n | n | no MA | no MA | n | n | no MA | n |
| Fleming et al. (2020) | 1/3 | y | n | n | py | n | n | n | py | Not required for scoping reviews | n | not applicable | not applicable | not applicable | y | not applicable | y |
| Frey et al. (2022) | 1/5 | n | n | n | py | n | n | n | py | n | n | no MA | no MA | n | n | no MA | n |
| Gracia et al. (2016) | 1/3 | y | n | n | py (with good will) | n | n | n | n (quite rudimentary table) | not applicable (recommended appraisal tools for qualitative research do not exist) | n | no MA | no MA | not applicable | not applicable | no MA | y |
| Hansen et al. (2022) | 6/7 | y | y | y | py | y | n | n | py | y | n | y | y (sensitivity analyses) | y | y | n | y |
| Henken et al. (2007) | 7/7 | y | y | n | y | y | y | y | y | y | n | no MA | no MA | y | y | no MA | y |
| Higgins et al. (2020) | 4/5 | y | py | n | y | y | y | n | py | y (although not required for scoping reviews) | n | no MA | no MA | y (with good will) | y | no MA | y |
| Krysinska et al. (2021) | 5/5 | y | y | n | py | n | y | n | py | y | n | no MA | no MA | y | y | no MA | y |
| Lohrasbi et al. (2022) | 1/5 | y | n | n | py | n | n | n | n (quite rudimentary table) | n | n | no MA | no MA | n | n | no MA | y |
| Ma et al. (2017) | 6/7 | y | py ) | n | py | n | n | y | y | y | y | y | n | n | n | y | n |
| Ma et al. (2020) | 5/7 | y | y | n | py | y | n | n | py | y | n | y | n | n | n | y (planned but not possible) | y |
| Macleod et al. (2011) | 1/5 | y | n | n | py | y | n | n | py | n (assessed but not reported, no validated tool) | n | no MA | no MA | n | y | no MA | y |
| McGovern et al. (2021) | 4/5 | y | py | n | py | y | y (likely according to author contributions) | n | py | y (with good will) | n | no MA | no MA | y | y | no MA | y |
| Meis et al. (2012) | 4/7 | y | n | y | py | n | y | n | py | y | n | y | n | y | y | n (at least not documented) | y |
| Meyer et al. (2017) | 2/3 | y | py | n | py | y | n | n | not applicable | not applicable | not applicable | not applicable | not applicable | not applicable | not applicable | not applicable | y |
| Morillo et al. (2022) | 4/5 | y | y | n | py | y | n | n | py | y | n | no MA | no MA | y | y | no MA |  |
| Morton et al. (2021) | 2/5 | y | n | n | py | n | n | n | py | n | n | no MA | no MA | y (with good will) | y | no MA | y |
| NICE 2018 | 7/7 | y | y | n | y | y | y | y | y | y | n | y | n | y | y | y | y |
| Norton et al. (2021) | 4/5 | y | py | n | py | n (unclear) | y | n | n (quite rudimentary table) | y | n | no MA | no MA | y | n | no MA | y |
| Okpokoro et al. (2014) | 5/5 | y | y | n | y | y | y | y | y | y | y | no MA | no MA | y | y | no MA | y |
| Petkari et al. (2020) | 3/5 | y | y | n | py | y | y | n | py | y | n | no MA | no MA | n | y | no MA | y |
| Pharoah et al. (2010) | 7/7 | y | y | n | y | y | y | py | y | y | n | y | n | y | y | y | y |
| Piat et al. (2015) | 1/5 | y | n | y | py | y | y | n | py | Not required for scoping reviews | n | no MA | no MA | not applicable | y | no MA | y |
| Rane et al. (2017) | 2/5 | y | py | n | py | y | y with good will | n | py | n | n | no MA | no MA | n | y | no MA | y |
| Reupert et al. (2013) | 1/3 | y | n | y | py | n | n | n | py | not applicable | n | not applicable | not applicable | not applicable | not applicable | not applicable | n |
| Rodolico (2022) | 7/7 | y | y | n | py | y | y | y | y/py (supplement not available) | y | y | y | y (sensitivity analyses) | y | y | y | y |
| Rushton et al. (2023) | 5/7 | y | py | n | py | y | n | n | py | RCTs = y NRSI = y | n | RCTs = y NRSI = no MA | n | y | n | n | y |
| Sin et al. (2017) | 6/7 | y | y | n | py | y | y | n | y | y | n | y | n | y | y | y | y |
| Spain et al. (2017 | 3/3 | y | y | n | y | y | n.a. | y | n.a. | n.a. | n.a. | n.a. | n.a. | n.a. | n.a. | n.a. | y |
| Stahl et al. (2016) | 2/5 | y | n | n | py | n | n | n | py | n (assessed but not reported) | n | no MA | no MA | y (although not formally assessed, but critically discussed) | y | no MA | y |
| Stiawa et al. (2014) | 1/5 | y | n | n | py | n | n | n | py | n | n | not applicable | not applicable | n | y | not applicable | y |
| Sutherland et al. (2020) | 2/5 | y | n | y | py | y (for full text screening) | y | n | py | n | n | no MA | no MA | y (although not formally assessed, but critically discussed) | y | no MA | n |
| Sutherland et al. (2023) | 1/5 | y | n | n | py | y | y | n | py | n | n | no MA | no MA | n | y | no MA | y |
| Thompson et al. (2017) | 3/5 | y | n | n | py | y (with good will) | n | n | py | y | n | no MA | no MA | y (with good will) | y | no MA | y |
| Thomson-Hollands et al. (2014) | 1/5 | y | n | n | py | y | n | n | py | n | n | unclear | no | n | y | no MA | n |
| van Es et al. (2023) | 6/7 | y | y | n | py | y | y | n | py (suppl.) | y | n | y | n | y (with good will) | y | y | n |
| Wang et al. (2021) | 3/7 | y | n | n | py | y | y | n | py (with good will) | y | n | y | n | n | y | n | y |
| Zinser et al. (2022) | 6/7 | y | y | n | py | y | y | n (suppl. Not available) | py | y | n | y | n | y | y | y (with good will) | y |
| AMSTAR 2 items:   1. Did the research questions and inclusion criteria for the review include the components of PICO? 2. Did the report of the review contain an explicit statement that the review methods were established prior to the conduct of the review and did the report justify any significant deviations from the protocol? 3. Did the review authors explain their selection of the study designs for inclusion in the review? 4. Did the review authors use a comprehensive literature search strategy? 5. Did the review authors perform study selection in duplicate? 6. Did the review authors perform data extraction in duplicate? 7. Did the review authors provide a list of excluded studies and justify the exclusions? 8. Did the review authors describe the included studies in adequate detail? 9. Did the review authors use a satisfactory technique for assessing the risk of bias (RoB) in individual studies that were included in the review? 10. Did the review authors report on the sources of funding for the studies included in the review? 11. If meta-analysis was performed did the review authors use appropriate methods for statistical combination of results? 12. If meta-analysis was performed, did the review authors assess the potential impact of RoB in individual studies on the results of the meta-analysis or other evidence synthesis? 13. Did the review authors account for RoB in individual studies when interpreting/discussing the results of the review? 14. Did the review authors provide a satisfactory explanation for, and discussion of, any heterogeneity observed in the results of the review? 15. If they performed quantitative synthesis did the review authors carry out an adequate investigation of publication bias (small study bias) and discuss its likely impact on the results of the review? 16. Did the review authors report any potential sources of conflict of interest, including any funding they received for conducting the review? | | | | | | | | | | | | | | | | | |

# S5 Extraction form for the studies selected

|  | operationalisation |
| --- | --- |
| study | citation |
| design | general information on the study design (e.g. systemativ review, all study designs, narrative synthesis)  authors background (nursing, inhospital care, community …)  investigators participative? yes/no (if yes: who and how?) |
| objective | overall objective of the review, research question |
| search period | date of systematic search |
| participants | psychiatric disorder: (psychosis, depression, miscellaneaus)  caregiver type: (parents, siblings, life partner/spouse, friends, others, miscellaneous)  if relevant: specific inclusion/exclusion criteria |
| interventions | exact name of the intervention according to the title or abstract;  detailed description if available in the methods section: |
| controls | according to methods section |
| outcomes/Purpose | user outcomes (according to methods section)  caregiver outcomes (according to methods section) |
| body of evidence | number of included studies, differentiated by study type; if reported, number of included participants (patients, caregivers)  publication years (range)  countries in which the studies were conducted  quality rating of the included studies (in short form with the tool used and the most important deficiencies) |
| AMSTAR2 rating | - number of AMSTAR2 items not met - specification of the unfulfilled critical items   number of cricital items depends on type of evidence synthesis; comment if necessary |
| results | **Decriptive results**   - study scientists background (nursing, inhospital care, community …) - participants details - patients   - disorder type   - disorder onset/first treatment contact   - mean age (range)   - percentage women (range) - caregivers   - type   - mean age (range)   - percentage women (range) - recruitment - interventions details (as detailed as possible) - duration and frequency - settings: (inhospital, outpatient …) - delivery format: (e.g. caregiver groups, mixed user/caregiver groups) - type (e.g. psychoeducation, counselling …) - concrete programme titles or used manuals - components/elements (e.g. individual sessions, home visits, workshops …) - personnel: (e.g. professional-led, self-led) - follow-up (range) |
|  | **Quantitative results**  patient outcomes   - most important quantitative endpoints incl. confidence interval, heterogeneity statements - measurement instruments used (e.g. questionnaires)   caregiver outcomes   - most important quantitative endpoints incl. confidence interval, heterogeneity statements - measurement instruments used (e.g. questionnaires) - results of subgroup analyses if useful - attrition/adherence/drop-out/acceptability |
|  | **Qualitative outcomes**  individual presentation of qualitative results, special consideration of figures and tables |
|  | **Limitations**  as reported from autors; additional remarks if applicable |
|  | **Authors conclusion**  from discussion section or abstract |
| qualitative richness | - None (review contains no qualitative statements) - Low (review contains only little qualitative content) - Moderate (review contains explicit qualitative statements, even if this was not the primary focus of the study) - High (review focuses on qualitative content or review was designed for qualitative content)   briefly assess with regard to a possible subsequent realist review,  note where this content can be found |

# S6 Characteristics of includes studies

###### *Note on wording: Since the reviews listed here used a different wording for the target groups analysed than we did in our working group (see article), the following table contains the terms of the review for the sake of simplicity.*

###### Al-Sawafi et al. 2020

| Al-Sawafi et al. 2020 | |
| --- | --- |
| study | Al-Sawafi, A., Lovell, K., Renwick, L., & Husain, N. (2020). Psychosocial family interventions for relatives of people living with psychotic disorders in the Arab world: systematic review. BMC Psychiatry, 20(1). https://doi.org/10.1186/s12888-020-02816-5 |
| design | systematic review, all study designs, narrative synthesis  authors background: Division of Nursing Midwifery & Social Work, The University of Manchester, College of Nursing/Sultan Qaboos, University of Manchester  investigators participative? no |
| objective | To synthesize the available evidence about culturally adapted psychosocial family interventions in the Arab world. |
| search period | 201908 |
| participants | psychiatric disorder: schizophrenia or related disorders  caregiver type: relatives or family members caring for an individual with schizophrenia or related disorders  inclusion criteria:  1) All study designs that evaluated or developed any type or format of culturally adapted psychosocial family interventions in the Arab world.  2) Participants who were relatives or family members caring for an individual with schizophrenia or related disorders.  3) The majority of carers (70% or above) were adults of 18 years or older, and the majority (70% or above) of people who had schizophrenia or related disorders based on ICD-10criteria.  exclusion criteria:  1) The intervention did not include family members or caregivers.  2) Languages were not Arabic or English. |
| interventions | Culturally adapted psychosocial family interventions (psychoeducation, family therapy, counselling, communication and problem-solving skills training or CBT). |
| controls | Not applicable. |
| outcomes/ purpose | patient outcomes: not predefined  caregiver outcomes: not predefined  review focuses on intervention characteristics, contents and components as well as strategies of cultural adaption; feasibility, acceptability |
| body of evidence | 6 studies: 3 RCTs, 2 NRSI, 1 cross-sectional; n=394 patients; n=344 caregivers  Sample size: 8 patients/9 caregivers to 63 dyads of patients and their caregivers  publication years: 2008-2018  countries: Jordan (2), Egypt (4)  quality rating of the included studies: not assessed |
| AMSTAR2 rating | - 1/5 critical items not met - No list of excluded studies |
| results | Descriptive results   - scientists background: not reported - participants details   - disorder type: schizophrenia   - disorder onset/first treatment contact: not reported   - caregiver type: no details reported   - recruitment: not reported   - mean age (range): not reported   - percentage women (range): not reported - interventions details   - duration and frequency: 8 weeks to 6 months; not reported in detail   - setting: 4x clinical setting in the outpatient department, 1 patients’ home (booklet); no inpatient setting   - delivery format: individual-family sessions, attended by patients and their caregivers; 1 group   - components: psychoeducation, partly + communication enhancement, problemsolving skills, stress management strategies, counselling   - personnel: Healthcare providers or researchers; 1 booklet only   - follow-up (range): not reported |
|  | Quantitative results  Patient outcomes:   - Symptom severity: sig. effects (not reported in detail as they are not valid because of poor quality and size) - Other outcomes: medication adherence, social functioning, quality of life and knowledge of schizophrenia   Caregiver/Family outcomes:   - family burden of care, attitude, carers’ quality of life: Family Burden Interview Scale (FBIS); Schizophrenics’ Carers’ Quality of Life Scale (S-CQoL); Caregiver Burden Scale; Opinions about Mental Illness Scale (OMI)   attrition/adherence/drop-out/acceptability: not reported |
|  | Qualitative results  Adaptation Model:   - BFT manual by (Mueser and Glynn, 1999); educational component from the psychoeducational program by ElShafie and colleagues (2002) - framework Atkinson and Coia, Bloom’s taxonomy of learning domains - The one intervention provided patients and caregivers with coping skills and knowledge to manage schizophrenia. Family members are important to improve the outcome for patients |
|  | Limitations   - small number of studies, small populations - high PICO heterogeneity - poor study quality; poor reporting/lack of information |
|  | Authors conclusion  "Furthermore, the data suggests that any family-oriented intervention for schizophrenia is likely to be better than standard care in improving the outcome for patients and their families." |
| qualitative richness | Low (review contains only little qualitative content, see chapter results, discussion) |

###### Ashcroft et al. 2018

| Ashcroft et al. 2018 | |
| --- | --- |
| study | Ashcroft, K., Kim, E., Elefant, E., Benson, C., Carter, J. A. (2018). Meta-Analysis of Caregiver-Directed Psychosocial Interventions for Schizophrenia. Community Mental Health Journal, 54(7), 983–991. https://doi.org/10.1007/s10597-018-0289-x |
| design | systematic review of RCT, meta-analysis  authors background: Janssen Scientific Affairs, LLC  investigators participative? no |
| objective | To compared CDPI versus treatment as usual (TAU) on outcomes such as hospitalization, relapse, non-compliance, and “other outcomes” (emergency services utilization, suicide attempt, and death). |
| search period | 2005–2015 |
| participants | psychiatric disorder: schizophrenia or schizophreniform disorder  caregiver type: "non-healthcare professional who has a close relationship with the patient and assumed responsibility for the patient’s welfare"; ≥ 18 years; "family members or individuals with a close relationship to the consumer"  inclusion criteria: article published in-full and peer-reviewed; RCT; outpatient treatment setting, randomization to either CDPI or TAU; outcomes measured at pre-defined intervals; <50% of patients lost to follow-up; patients and caregivers ≥ 18 years of age; caregivers are family members or close relationship; caregivers were not service providers of the investigated health services  exclusion criteria: not reported |
| interventions | Psychosocial interventions directed toward the caregivers (CDPI) administered by a health care professional in a pre- defined format over multiple sessions. |
| controls | TAU (any treatment for schizophrenia that did not include a caregiver-directed component) |
| outcomes/ purpose | patient outcomes: inpatient hospitalization, relapse, non-compliance with any intervention received in the study period, and “other events” (suicide attempt, psychiatric emergency services utilization, death)  caregiver outcomes: none |
| body of evidence | 18 RCTs; n range 34–1239  mean n consumer/caregiver pairs 98,4 (SD 142,6); mean controls 91,4 (SD 147,9)  publication years: 2006 to 2015  countries: not reported  quality rating of the included studies: not assessed |
| AMSTAR2 rating | - 5/7 critical items not met - No protocol, No list of excluded studied, No quality assessment of included studies, Risk of bias not mentioned in discussion, Publication bias not assessed - Reporting poor; systematic review standards not fulfilled |
| results | Descriptive results   - scientists background: not reported - participants details   - disorder type: schizophrenia   - disorder onset/first treatment contact: not reported   - caregiver type: not reported   - recruitment: not reported   - mean age (range): not reported   - percentage women (range): not reported - interventions details   - duration and frequency: 57 weeks (± 32), mean number of sessions 28 (± 17); 0.56 (± 0.34)   - sessions per week on average   - setting: not reported in detail, „pre- defined format over multiple sessions"   - delivery format: not reported   - components: not reported   - personnel: not reported in detail; "administered by a health care professional"   - follow-up (range): not reported |
|  | Quantitative results  patient outcomes   - CDPI vs TAU - Relapse: 109/397 vs. 185/382; RR 0,58 (0,47 vs. 0,73); I^2^=32%; 13 RCTs, n=779; - Suicide attempt: 4/55 vs. 3/45; n.s.; 2 RCTs, n=100 - Emergency Service Utilization: 77/107 vs. 95/292; n.s.; 2 RCTs, n=399 - Medication compliance: 18/111 vs. 24/91; n.s.; 3 RCTs; n=209 - Clinical activities compliance: 7/124 vs. 32/114; RR 0,22 (0,11; 0,47); I^2^=0%; 3 RCTs, n=238   caregiver outcomes: not reported  attrition/adherence/drop-out/acceptability: not reported |
|  | Qualitative results  none |
|  | Limitations  Small sample sizes, heterogeneity of outcome definitions; heterogeneity of intervention duration and frequency. |
|  | Authors conclusion  "CDPI is associated with reductions in hospitalization, relapse, and treatment non-compliance." |
| qualitative richness | None (review contains no qualitative statements) |

###### Bademli et al. 2011

| Bademli et al. 2011 | |
| --- | --- |
| study | Bademli K & Cetinkaya Duman Z (2011). Family to family support programs for the caregivers of schizophrenia patients: a systematic review. Turk Psikiyatri; 22(4), 255-65. |
| design | systematic review, all study designs, narrative synthesis  authors background: psychiatric nursing  investigators participative? no |
| objective | To analyze the family to family support programs provided for the families of the schizophrenic patients and there potential impact on caregivers. |
| search period | 2009 |
| participants | psychiatric disorder: schizophrenia  caregiver type: family |
| interventions | family to family support/training programs administered to the caregivers  exclusion: programs given by healthcare workers |
| controls | Not applicable. |
| outcomes/ purpose | patient outcomes: not predefined  caregiver outcomes: not predefined |
| body of evidence | 12 studies; sample size 37 to 462  publication years: 1997 to 2008  countries: not reported  quality rating of the included studies: not reported |
| AMSTAR2 rating | 4/5 critical items not met   - No protocol, no list of excluded studies, no quality assessment, risk of bias not mentioned in discussion - Review is not fulfilling methodological requirements for systematic reviews. |
| results | Descriptive results   - scientists background: not reported - participants details   - disorder type: schizophrenia   - disorder onset/first treatment contact: not reported   - caregiver type: not reported in detail   - recruitment: not reported   - mean age (range): not reported   - percentage women (range): not reported - interventions details   - duration and frequency: 8-12 weeks, mostly weekly sessions; 60-180 min   - setting: not reported   - delivery format: individually, group or both   - components: psychoeducation, communications, problem solving, coping, psychosocial needs, counselling   - personnel: previously trained caregivers or health professionals/ psychiatric nurses   - follow-up (range): mostly 6 months |
|  | Quantitative results  Patient outcomes: not reported  Caregiver outcomes   - "caregivers had more information on the causes and treatment of mental disease, coped better with the difficulties they experienced, their negative feelings decreased, their satisfaction levels increased, their social support was enhanced and their concerns about the patient were alleviated" - Used instruments   - Family Support Services Index   - Family Burden Interview Schedule   - Family Assesment Device   - Family Empowerment Scale   - Center for Epidemiological Studies Depression Scale   - General Health Questionnaire   - The Family Information Needs Scale   - The Caregiving Satisfaction Scale   - Family Knowledge Scale and Self Efficacy Scale   attrition/adherence/drop-out/acceptability: not reported |
|  | Qualitative results  None. |
|  | Limitations  No quality assessment; abstract misses "peer“as common wording for caregiver-to-caregiver interventions. |
|  | Authors conclusion  "… although program content was designed to increase coping skills of caregivers, they did not examine the effect of the program on the coping ability of the of care givers … The psychiatry nurse should participate in the implementation and planning of family to family support programs and in the process of evaluating the effect on families and follow-up." |
| qualitative richness | Low (review contains only little qualitative content, see chapter discussion) |

###### Barbeito et al. 2020

| Barbeito et al. 2020 | |
| --- | --- |
| study | Barbeito, S., Sánchez-Gutiérrez, T., Becerra-García, J. A., Pinto, A. G., Caletti, E., & Calvo, A. (2020). A systematic review of online interventions for families of patients with severe mental disorders. Journal of Affective Disorders, 263, 147–154. https://doi.org/10.1016/j.jad.2019.11.106 |
| design | systematic review, all study designs, narrative synthesis  authors background: Health Sciences, Neurosciences and Mental Health  investigators participative? no |
| objective | To clarify whether online treatments are effective, well accepted, and feasible for improving symptoms in patients with severe mental disorders and their families. |
| search period | 201806 |
| participants | psychiatric disorder: severe psychiatric disorders (first episode psychosis, schizophrenia, schizoaffective, bipolar disorder, psychotic disorder); at least 50% psychotic spectrum disorder  caregiver type: informal family caregivers |
| interventions | psychological online interventions |
| controls | Not predefined. |
| outcomes/ purpose | patient outcomes: symptoms, adherence "Outcomes were optional for patients."  caregiver outcomes: acceptance, satisfaction |
| body of evidence | 9 studies (4 RCTs), n=1329 family caregivers  publication years: 2002 to 2017  countries: not reported  quality rating of the included studies: not assessed |
| AMSTAR2 rating | 3/5 critical items not met   - No protocol, no list of excluded studies, no quality assessment |
| results | Descriptive results   - scientists background: not reported - participants details   - disorder type: 1x first episode psychosis, 5 Schizophrenia/Psychosis, 1 bipolar, 2 miscellaneous with at least 50% psychotic disorder   - disorder onset/first treatment contact: not reported   - caregiver type: family relatives (spouse, siblings, parents)   - recruitment: not reported   - mean age (range): 33-50 years for patients, 29-54 for caregivers (not all studies reported mean age)   - percentage women (range): 79,26% - interventions details: not summarized   - components: psychoeducation, peer discussion, support forum |
|  | Quantitative results  Narrative synthesis, superficial reporting "well accepted, with good results both for patients and for family members"  Patient outcomes   - symptoms - perceived stress - social support - hospitalisations   Caregiver/family outcomes   - acceptability, feasibility, usefulness - family knowledge - perceived stress - burden (relational problems, mental health, and difficulties with daily activities) - self-compassion - social support   Used instruments   - RDS: Ratings of disease-related distress - SSQ: Social support - WEI: Web site using the Website Evaluation Instrument - SAPS: Scale for the Assessment of Positive Symptoms - PEUQ: Perceived Ease of Use Questionnaire - BPRS: Brief Psychiatric Rating Scale - BSI: Brief Symptom Inventory - FAS: Family Attitude Scale - PSS: The Perceived Stress Scale - DAS: Depression, Anxiety Stress Scale (DASS; 38). - SPW: Scales of Psychological Well-being (SPW; 39). - MOD-SSS: Medical Outcomes Study: Social Support Survey ( - FFMQ: Five Facet Mindfulness Questionnaire (FFMQ) CarerQoL7-D - SCS-SF: Self-Compassion Scale-Short Form (SCS-SF) - SUS: Usability, confounding factors, and negative effects of Training   attrition/adherence/drop-out/acceptability: not reported |
|  | Limitations  Few studies, few RCTs, partly only on feasibility, high PICO heterogeneity. |
|  | Authors conclusion  "… online intervention programs are superior to standard of care (with respect to reducing caregivers’ symptoms, such as perceived stress and burden), help to improve knowledge, and enable experiences to be shared. In addition, patients with severe mental disorders who underwent online intervention programs experienced relief from their symptoms (positive) of severe mental disorder, had fewer hospitalizations, and seemed to have a higher perceived level of social support and good acceptance of online programs." |
| qualitative richness | None (review contains no qualitative statements). |

###### Baruch et al. 2018

| Baruch et al. 2018 | |
| --- | --- |
| study | Baruch, E., Pistrang, N., & Barker, C. (2018). Psychological interventions for caregivers of people with bipolar disorder: A systematic review and meta-analysis. Journal of Affective Disorders, 236, 187–198. https://doi.org/10.1016/j.jad.2018.04.077 |
| design | Systematic review of RCT/pseudo-RCT; meta-analysis  authors background: Clinical, Educational and Health Psychology  investigators participative? no |
| objective | This review examined the efficacy of psychological interventions in improving caregiver-focused outcomes, including burden, psychological symptoms and knowledge. |
| search period | 201710 |
| participants | psychiatric disorder: bipolar disorder  caregiver type: relatives, spouses, partners, friends and neighbours |
| interventions | Psychological therapy, psychoeducational interventions, support groups  Note: Interventions could involve the person with bipolar disorder as well as their caregiver, or caregivers alone |
| controls | Treatment as usual, a waitlist control, or an alternative intervention, where this was clearly intended to be a control for the intervention of interest |
| outcomes/ purpose | patient outcomes: none  caregiver outcomes: burden, psychological symptoms or knowledge |
| body of evidence | 9 studies included (8 RCTs), 8 included in meta-analysis  n= 224  publication years: 1990 to 2016  countries: 2x USA, Brazil, Italy, Australia, Greece, Ireland, Spain, The Netherlands  quality rating of the included studies: 5x low, 3x unclear, 1x high risk of bias (all: no blinding) |
| AMSTAR2 rating | 2/7 critical items not met  No list of excluded studies; publication bias not assessed |
| results | Descriptive results   - scientists background: not reported - participants details (   - disorder type: bipolar disorder   - disorder onset/first treatment contact: not reported   - caregiver type: mostly parents (28-71%), spouses/partners (6-100%)   - recruitment: radio, clinics, university, NGOs. Media, mental health services, support groups, outpatient clinics, volunteers from allied research study   - mean age (range): 44,1 to 53,3 (carers), 32,2-47,1 (patients)   - percentage women (range): 53% to 84% (carers), 63% to 67% (patients) - interventions details   - duration and frequency: 2-18 sessions á 45-150 minutes   - setting: 1x inpatient setting, 8x community setting   - delivery format: 5x group format (carers only), 2x individual (carers only), 2x family sessions   - type: psychoeducational   - components: education, coping strategies, communication skills, problem-solving skills   - personnel: psychology, psychiatry, family therapy, nursing; n=2 therapists had received training and supervision   - follow-up (range): 1 to 24 months |
|  | Quantitative results  Note: n=2 both caregivers and patients participated; n=3 patients’ outcomes were reported even though they did not participate.  Patient outcomes   - Assessed: symptomatology, functioning - Not reported/meta-analysed   Caregiver outcomes   - Outcomes assessed: subjective/objective burden, psychological symptoms, knowledge, quality of life, self-esteem, health risk behaviour, psychosocial problems, social support, attitudes and family relationships. - Psychoeducation vs. control - burden at post-treatment: g = −0.8 (95% CI: −1.32, −0.27). I² = 81%; 6 studies (N = 379) - burden at follow-up: g = −1.22 (95% CI: −3.19, 0.75). I² = 95%; 3 studies (N = 137) - psychological symptoms at post-treatment: g = −1.76 (95% CI: −4.21, 0.70). I² = 97%; 3 studies (N = 155) - psychological symptoms at follow-up: g = −2.44 (95% CI: −5.91, 1.03), I² = 97%; 2 studies (N = 101) - knowledge at post-treatment: g = 2.60 (95% CI: 1.39, 3.82). I² = 88%; 4 studies (N = 183) - knowledge at follow-up: g = 2.41 (95% CI: 0.85, 3.98). I² = 89%; 3 studies (N = 127) - Effects on burden were large (global burden), moderate to small (subjective burden) and no effect for objective burden. - Psychoeducation vs. active psychological intervention - 1 study, 1-year follow-up - Knowledge: g = 0.80 (95% CI: 0.01, 1.59) in favor of solution-focused group psychotherapy - Burden, symptoms: unclear results   attrition/adherence/drop-out/acceptability: not reported |
|  | Qualitative results  None. |
|  | Limitations  substantial heterogeneity between studies (mainly driven by Kolostoumpis et al., 2015); confidence intervals were wide; some studies had substantial methodological limitations; diversity of measures used, severity of symptoms in patients; clinical and community samples may not be comparable; small sample sizes; small study size. |
|  | Authors conclusion  “This review provides tentative meta-analytic evidence for the efficacy of psychoeducation in improving caregiver burden at post-treatment, and knowledge at post-treatment and follow-up. The lack of an effect for psychological symptoms could suggest that more targeted interventions are needed to address the needs of caregivers experiencing greater levels of distress.” |
| qualitative richness | None (review contains no qualitative statements). |

###### Baudinet et al. 2021

| Baudinet et al. 2021 | |
| --- | --- |
| study | Baudinet, J., Eisler, I., Dawson, L., Simic, M., & Schmidt, U. (2021). Multi‐family therapy for eating disorders: A systematic scoping review of the quantitative and qualitative findings. International Journal of Eating Disorders, 54(12), 2095–2120. https://doi.org/10.1002/eat.23616 |
| design | Systematic scoping review  Parallel-results convergent synthesis design (qualitative and quantitative data analysed separetely and then synthesized together |
| objective | To review the quantitative and qualitative evidence-base for multi-family therapy (MFT) for eating disorders regarding change in physical and psychological symptoms, broader individual and family factors, and the experience of treatment. Further:  1. To review the impact of MFT on the physical and psychological symptoms of eating disorders.  2. To review the impact of MFT on families and caregivers.  3. To review the individual and family experience of receiving MFT. |
| search period | 20210624 |
| participants | psychiatric disorder: eating disorder (note: not adults only)  caregiver type: family/caregivers |
| interventions | Multi-family therapy (MFT) |
| controls | Not restricted. |
| outcomes/ purpose | patient outcomes: weight, psychopathology, mood, negative impacts, physical health, family functioning, %mBMI, level of care, stigma, emotional awareness/regulation  caregiver outcomes: general wellbeing, expressed emotions, symptoms of depression, percecived social support, impact of stigma, isolation, parental mood, |
| body of evidence | 27 studies: 3 RCTs (adults: 1), 4 non-randomized comparison studies (adults: 1), 10 case series (adults: 3), 10 qualitative studies (adults: 3), 1 mixed-methods (adults: 0)  N = 914  publication years: 2000 to 2020  countries: UK, Canada, USA, Sweden, Belgium, Denmark, Czech Republic, Norway  quality rating of the included studies: not assessed (not required for scoping reviews) |
| AMSTAR2 rating | - 2/3 critical items not met - No protocol; no list of excluded studies - AMSTAR2 applicable to scoping reviews only to a limited extent |
| results | Descriptive results  note: N and mean ranges also include studies with only patients and no caregivers   - scientists background: not reported - participants details   - disorder type: eating disorder   - disorder onset/first treatment contact: not reported   - caregiver type: caregiver (not specified) or family   - recruitment: not reported   - mean age (range): Young people outpatient 13,9 to 16. Young people inpatient 14,3 to 17,1. Adult outpatient 21,3 to 24,5. Adult inpatient 26,6 to 45,7.   - percentage women (range): 100% to 90% - interventions details   - duration and frequency: not reported   - setting: 4x inpatient, 12x outpatient, specialist ED service   - type: 10x Maudsley (full or inspired), 1x Cognitive-Interpersonal Maintenance Model of Anorexia Nervosa, 1x Family workshops, 1x Neurobiologically informed intensive family therapy, 1x manualized R4R MFT Group, 1x Psychoeducation groups, 1x Wallin model, 1x Family therapy   - components: not reported   - personnel: not reported   - follow-up (range): 3 to 30,9 months |
|  | Quantitative results  (adult studies only)   - Adult-Inpatient MFT vs. single-family therapy (1 RCT, n=48): Caregiver outcomes (Whitney et al. 2012)   - patient: higher BMI at 6-month follow-up, but lower BMI at 36-month follow-up (not stat. sig.)   - caregiver: expressed emotion, general wellbeing, negative/positive aspects of caregiving: no differences between the treatments   - drop-out: 13% (MFT) vs. 12% (FT) - Adult-Inpatient MFT vs. single-family therapy (1 non-randomized comparison study, n=45): Caregiver outcomes (Dimitropoulos et al. 2015)   - Patient: BMI and eating disorder psychopathology: no differences between the treatments   - caregiver factors (perceived burden, expressed emotion, perceived social supports, and stigma): no differences between the treatments   attrition/adherence/drop-out/acceptability: not reported   - drop-out: 17% (MFT) vs. 18% (FT) |
|  | Qualitative results  (From intervention studies Whitney (2012) and Dimitropoulos (2015) only):  Themes: take on new perspectives; helped particularly parents/caregivers, feel less guilty, scared, and anxious and feel more confident; value placed on being able to observe and learn from other families who had similar experiences; intensity of the group was also mentioned by some participants as both helpful and exhausting. |
|  | Limitations  No exhaustive review of all MFT models; small sample sizes; many different study methodologies; homogenic samples (predominantly white and female), only little data about bulimia nervosa. |
|  | Authors conclusion  “The current review suggest MFT is an effective treatment for anorexia nervosa and leads to improvements in individual, as well as some caregiver and family factors.” |
| qualitative richness | Moderate (review contains explicit qualitative statements. |

###### Brady et al. 2016

| Brady et al. 2016 | |
| --- | --- |
| study | Brady, P., Kangas, M., & McGill, K. (2016). “Family Matters”: A Systematic Review of the Evidence for Family Psychoeducation for Major Depressive Disorder. Journal of Marital and Family Therapy, 43(2), 245–263. https://doi.org/10.1111/jmft.12204 |
| design | systematic review, all study designs, narrative synthesis  authors background: university, Mental Health Service  investigators participative? No. |
| objective | To evaluate the efficacy of face-to-face FPE for persons with depression and their carers; and to evaluate whether the effectiveness was dependent upon delivery mode, including comparison of outcomes for (a) multi-family and single-family psychoeducation, (b) peer-led and clinician-led groups, and (c) FPE groups for MDD only compared to FPE for any mental illness (mixed-diagnosis groups). |
| search period | 1985-201601 |
| participants | psychiatric disorder: Major Depressive Disorder  caregiver type: family |
| interventions | Psychoeducation incl. skills buiding; clinician or peer led; open or closed group formats  Exclusion: CBT, self-help (bibliotherapy) |
| controls | Not restricted |
| outcomes/ purpose | patient outcomes: depressive symptoms, well-being  caregiver outcomes: depressive symptoms, well-being |
| body of evidence | 8 studies (7 samples, 5 RCTs)  publication years: 1990 to 2015  countries: 1x Japan, 1x Italia, 1x Belgium, 1x Indian, 1 Germany  quality rating of the included studies: 5/10 adequate-to-strong, but only 2x blinded outcome assessment |
| AMSTAR2 rating | - 2/5 critical items not met - No protocol, no list of excluded studies |
| results | Descriptive results   - scientists background: not reported - participants details:   - disorder type: major depressive disorder   - disorder onset/first treatment contact: not reported   - caregiver type: as many family members who opted to be involved, spouses …   - recruitment: not reported   - mean age (range): mixed (partly adolescents), not always reported   - percentage women (range): only reported for carers, not always reported - interventions details   - duration and frequency: not summarized   - setting: at home, inpatient, outpatient   - delivery: group or family/partners   - components: 4x single-family psychoeducational (SFPE, after "Falloon-Model"), 4x multifamily psychoeducational (MFPE, after "McFarlane-Model"), 1x SFPE vs, MFPE   - components: education, family attitude, coping strategies, communication skills, problem solving   - personnel: not reported   - follow-up (range): not summarized   adaption of schizophrenia family psychoeducation models:   - single-family psychoeducation (SFPE, after "Falloon-Model")   - patients included   - focusing on patient's outcomes, but mostly reporting caregiver outcomes too   - mostly positive effects for patient's outcomes - multifamily psychoeducation (MFPE, after "McFarlane-Model")   - patients not included (not consistent with McFarlane!)   - nearly no patient outcome reporting (focusing on carer outcomes as family functioning, burden …)   - no convincing effects |
|  | Quantitative results  reported narratively and separately for each study  patient outcomes: Patient Symptoms and Functioning   - 6/7 studies measuring patient variables 🡪 positive outcomes - 1/7 studies 🡪 worse effect on patient functioning   caregiver outcomes: Family Attitude, Burnout, distress, Negative consequences of Care giving   - 4/6 studies measuring carer well-being or carer burden 🡪 positive effects - not consistently improvements in family attitudes/expressed emotion   „MFPE is at least as effective as SFPE in improving outcomes for patients with MDD; MFPE may be more effective than SFPE in improving outcomes for family–carers."  attrition/adherence/drop-out/acceptability: not reported:   - "no studies reported level of session attendance by participants" |
|  | Qualitative results  None. |
|  | Limitations  Few RCTs, no attendance reporting, nearly no follow-up outcome reporting, heterogeneity in assessed measures, methodological limitations |
|  | Authors conclusion  "The results from this systematic review highlight the promising outcomes that may be gained by offering FPE for depression for both the patient and their family–carers. The findings also provide preliminary evidence that MFPE may be as effective as SFPE for patient outcomes and may be potentially more effective for family–carers." |
| qualitative richness | None (review contains no qualitative statements) |

###### Camacho-Gomez & Castellvi 2019

| Camacho-Gomez 2019 | |
| --- | --- |
| study | Camacho-Gomez, M., & Castellvi, P. (2019). Effectiveness of family intervention for preventing relapse in First-Episode psychosis until 24 months of follow-up: a systematic review with meta-analysis of randomized controlled trials. Schizophrenia Bulletin, 46(1), 98–109. https://doi.org/10.1093/schbul/sbz038 |
| design | systematic review of RCT with meta-analysis  authors background: Department of Psychology, University of Jaén, Spain  investigators participative? no |
| objective | To assess the effectiveness of family intervention for psychosis for preventing second episode psy chosis (SEP) and other relapse-related outcomes until 24 months of follow-up in FEP (first episode psychosis) patients within the first 5 years of onset compared to treatment as usual (TAU) and/or TAU plus other active psychosocial interventions including randomized controlled trials (RCTs) only. |
| search period | 201806 |
| participants | psychiatric disorder: psychosis  caregiver type: "family"  inclusion criteria: first episode psychosis <5 years, RCT design  exclusion criteria: intervention <6 month |
| interventions | family intervention for psychosis ≥6 months +/- pharmacotherapy |
| controls | TAU, TAU + other psychosocial interventions (eg. psychoeducation) |
| outcomes/ purpose | patient outcomes: relapse, duration of hospitalization, symptom severity, functionality  caregiver outcomes: not reported |
| body of evidence | 11 studies (14 articles), RCT only, n= 1360; sample size 34-547  publication years: 1994 to 2014  countries: not reported; continents: 5 Asia, 5 Europe, 1 Australia.  quality rating of the included studies: 4x good, 4x fair, 3x poor (minding no blinding of participants and personnel, partly blinded outcome assessment) |
| AMSTAR2 rating | - 2/7 critical items not met - no protocol, risk of bias not mentioned in discussion |
| results | Descriptive results  study scientists background: in SR not reported   - participants details   - disorder type: psychosis   - disorder onset/first treatment contact: not reported   - caregiver type: "family", "parents were the main caregivers"; sample size of family members: range 1-15 per patient   - recruitment: not reported   - mean age (range): patients mean age 16,1-28,1 (range 16-40) years; caregiver age: "majority between 30 and 50 years”   - percentage women (range): not reported - interventions details: very heterogen   - duration and frequency: 6-18 month   - setting: not reported for all studies, partly assertive community treatment   - delivery format: 5/11 relatives+patients   - components: 6x manual-based (Falloon et al.; McFarlane et al.): (structured) psychoeducation, communication skills and problem solving; 3x family multi-component integrated treatment (eg, CBT + relapse prevention therapy); Assertive community treatment provided by multidisciplinary teams (1:10 caseload): social skill training; psychoeducational multifamily groups focusing on problem-solving procedures; Family-led mutual support groups and (b) Group psychoeducation for caregivers   - personnel: mostly not reported, 1x family-led (mutual support groups)   - follow-up (range): 6-24 month |
|  | Quantitative results  Patient outcomes   - relapse risk vs. TAU: 17/144 vs. 40/130; RR 0,36 (0,21; 0,63); I^2^=9%; 4 studies - relapse risk vs. TAU + active intervention: 14/64 vs. 30/64; RR 0,48 (0,27; 0,86); I^2^=14%; 2 studies - relapse risk vs. any control: 31/208 vs. 70/194; RR 0,42 (0,29; 0,61); I2=1%; 6 studies - days of hospitalization vs. TAU: MD -3,31 (-6,48; -0,14)); I^2^=71%; 6 studies, n=857 - symptom severity vs. TAU: SMD -0,68 (-1,14; -0,22); I^2^=84%; 5 studies, n=375 - functionality level vs. TAU: SMD 1,53 (0,63; 2,44); 5 studies, I^2^=95%; n=676   Caregiver outcomes   - not reported   attrition/adherence/drop-out/acceptability: not reported   - adherence rates: 43%-100% |
|  | Qualitative results  none |
|  | Limitations   - high PICO heterogeneity in domains P, I, C, O (i.e. different relapse definitions) - no blinding of participants and personnel (partly blinding for outcome assessment) |
|  | Authors conclusion  "These findings suggest that FIp is effective for reducing relapse rates, duration of hospitalization, and psychotic symptoms, and for increasing functionality in FEP patients up to 24 months."  (FIp = Family Intervention for psychosis) |
| qualitative richness | None (review contains no qualitative statements) |

###### Davies et al. 2021

| Davies et al. 2021 | |
| --- | --- |
| study | Davies, G., Deane, F. P., Williams, V., & Giles, C. (2021). Barriers, facilitators and interventions to support help‐seeking amongst young people living in families impacted by parental mental illness: A systematized review. Early Intervention in Psychiatry, 16(5), 469–480. https://doi.org/10.1111/eip.13194 |
| design | systematic review, all study designs, narrative synthesis  authors background: Psychology, Kids Foundation  investigators participative? no |
| objective | To synthesize the research describing barriers, facilitators and specific elements of interventions aimed at promoting help seeking among young people living in families affected by parental mental illness. |
| search period | 2005-202005  (2005 was set as the boundary to appropriately reflect increasing accessibility to help seeking resources such as online forums) |
| participants | psychiatric disorder: mental illness  caregiver type: children with at least one parent with mental illness |
| interventions | Any therapeutic strategy aiming to promote protective factors for young people living in families affected by parental mental illness.  Note: qualitative studies on barriers and facilitators were included too |
| controls | Not applicable. |
| outcomes/ purpose | patient outcomes: none  caregiver outcomes: help-seeking, operationalized as the sharing of information about a mental health problem to others, including informal (peers, family) and formal (mental health professionals, GP) sources, with the aim of gaining support that would reduce personal distress |
| body of evidence | 11 studies; of these 9 interventions studies, 1 RCT  countries: not reported; continents: Australia, Europe, UK  quality rating of the included studies: medium to high quality with moderate risk of response bias |
| AMSTAR2 rating | - 2/5 critical items not met - No protocol, no list of excluded studies |
| results | Descriptive results   - scientists background: not reported - participants details: not summarized/not reported   - mean age (range): 8-25 years - interventions: not summarized   - core components: 8/9 psychoeducation (educational booklets, online resources, role plays, creative activities); 4/9 peer support (creative and physical activities and group/forum discussions); 5/9 accessibility of help 24/7   - Specific programs: Kookaburra Kids |
|  | Quantitative results  Not reported for each study.  **Quantitative results** from an evaluation of this program [kookaburra kids] reported significant increases in help seeking intentions specific to the telephone helpline source. |
|  | Qualitative results  Barriers for help-seeking   - Stigma (preventing from searching professional help due to fear of taken into foster care and bullying) - Family communication (secrecy about illness, distrust by children and/or parents in public health system) - Lack of belonging or sense of shared experience (difficulty being vulnerable and establishing trust, fear of burdening others, sense of guilt and insecurity in relationships imposes greater reluctance to seek help)   Facilitators for help-seeking   - Individual characteristics (female, later age of problem onset, presence of a mood disorder) - Group identification - Anonymity (in online forums) |
|  | Limitations  Paucity of experimental and pre-post designed studies, not account for specific parental disorders, heterogene outcome measures, small number of studies. |
|  | Authors conclusion  "This at-risk group is affected by negative societal attitudes towards mental illness and this appears to have consequences for their helpseeking intentions and behaviours. Schools and communities have the potential to be key agents in negating the pervasive stigma towards mental illness and also facilitating help-seeking behaviours." |
| qualitative richness | High (review focuses on qualitative content or review was designed for qualitative content) |

###### Dehbozorgi et al. 2023

| Dehbozorgi et al. 2023 | |
| --- | --- |
| study | Dehbozorgi, R., Shahriari, M., Fereidooni-Moghadam, M., & Moghimi-Sarani, E. (2023). Family-centered collaborative care for patients with chronic mental illness: A systematic review. Journal of Research in Medical Sciences, 28(1). https://doi.org/10.4103/jrms.jrms_410_22 |
| design | systematic review, all study designs incl. qualitative research, narrative synthesis  authors background: Nursing and Midwifery  investigators participative? no |
| objective | to examine whether family centered collaborative care is an acceptable treatment option for individuals with chronic mental illness |
| search period | 2000-20210427 |
| participants | psychiatric disorder: chronic mental illness  caregiver type: family |
| interventions | family centered collaborative care (FCCC) |
| controls | Not applicable. |
| outcomes/ purpose | patient outcomes: not predefinded  caregiver outcomes: not predefinded |
| body of evidence | 15 studies (11 RCTs) (27 articles)  publication years: 2001 to 2019  countries: 4x USA; 1x Colombia, 2x Australia, 1x India, 4x UK, 3x Iran, 1x Thailand, 3x Spain, 1x Turkey, 1x China, 1x Pakistan, 1x Ethiopia, 1x Canada, 1x America, 2x NA  quality rating of the included studies: 7x good, 15x moderate, 7x poor |
| AMSTAR2 rating | - 4/5 critical items not met - No protocol, no bias assessment and discussion, no list of excluded studies - Review does not fulfill methodological requirements for systematic reviews. |
| results | Descriptive results   - scientists background: not reported - participants details   - disorder type: not reported for all included studies; reported: schizoaffective disorder, schizophrenia (n=1052), major depressive disorder (n=343), bipolar disorder (n=763);   - disorder onset/first treatment contact: not reported   - caregiver type: not reported   - recruitment: not reported   - mean age (range): not reported   - gender: not reported for all included studies - interventions details   - duration and frequency: not reported   - setting: often at home, but not always described   - delivery format: not reported   - components: very heterogen combinations; family education, communication skills and interpersonal relations, family’s skills to caring, discharge planning, psycho‑social intervention, problem-solving …   - personnel: not reported   - follow-up (range): not reported |
|  | Quantitative results  Patient outcomes   - relapse rate, re-hospitalization, social functioning, social cognition, quality of life, general well-being, symptoms, adherence, maladaptive emotional schemes (all improved) - effect estimates not reported "FCCC reduces relapse rate and re‑hospitalization"/“no conclusive evidence for the effectiveness for participants"   Family outcomes   - family functioning, family relationship functioning, caregiver burden, guilt, empathy - effect estimates not reported „significant reduction in caregiver burden"   attrition/adherence/drop-out/acceptability: not reported |
|  | Qualitative results   - None. |
|  | Limitations   - "… differentiating between family interventions and FCCC was challenging." - Narrative reporting for single studies, no/nearly no synopsis - Contradictory statements within the review, Unclear statements probably due to english language problems |
|  | Authors conclusion  "The findings demonstrated that family involvement in the care of patients with CMI affects no recurrence of the disease, and no re hospitalization of patients with this disorder." |
| qualitative richness | Low (review contains only little qualitative content, see chapter results/discussion) |

###### Dirik et al. 2017

| Dirik et al. 2017 | |
| --- | --- |
| study | Dirik, A., Sandhu, S., Giacco, D., Barrett, K., Bennison, G., Collinson, S., & Priebe, S. (2017). Why involve families in acute mental healthcare? A collaborative conceptual review. BMJ Open, 7(9), e017680. https://doi.org/10.1136/bmjopen-2017-017680 |
| design | "conceptual“ review, all study designs, narrative synthesis  authors background: Social and Community Psychiatry, Mental Health Services Development  investigators participative? **yes** |
| objective | To explore the theoretical background of family involvement models in acute mental health treatment and how this relates to their delivery. |
| search period | 20240119 |
| participants | psychiatric disorder: severe mental illness, acute phase  caregiver type: family |
| interventions | programs designed for family involvement in acute mental health treatment |
| controls | Not applicable. |
| outcomes/ purpose | Clustering of family involvement models, identifying program components, exploring the role of patients and carers. |
| body of evidence | 16 key articles  quality rating of the included studies: not assessed  countries: Canada, France, UK, USA, Germany, Finland |
| AMSTAR2 rating | - 3/3 critical items met - AMSTAR2 only partially applicable to qualitative reviews |
| results | Descriptive results  Not applicable. |
|  | Quantitative results  None. |
|  | Qualitative results  Family involvement models in acute mental healthcare:   - Calgary Family Assessment and Intervention Models (Canada) - ERIC (Equipe Rapide d’Intervention de Crise) - Family Psychoeducation Models (UK, USA) - Family Systems Approach, SYMPA (systems therapy methods in acute psychiatry) (Germany) - Open Dialogue (Finland) - Somerset Model (UK)   theoretical references underpinning the models:   - System theories (systemic family therapy) - Diathesis-stress theory (family psychoeducation models for schizophrenia; expressed emotion) - Postmodern theories   components of the approaches   - Communication/language use - Joint decision-making and the role of experts - Support for the family themselves - Wider social network involvement - Medication use - System organisation   Role of patients and families   - Families are a resource (adherence, information) - Linear roles and relationships   - There is a ‘patient’ and a ‘carer’   - Families want to help   - Family involvement is always beneficial - Risk of identity loss (patients) - Implementation versus choice |
|  | Limitations  Findings are by no means an exhaustive list of all existing family involvement models; unclear translation to other settings (rural environments, more traditional family structures). |
|  | Authors conclusion  "Although family involvement models have been developed in the context of diverse theoretical perspectives and sociopolitical events, there are many commonalities in their components.” |
| qualitative richness | High (review focuses on qualitative content or review was designed for qualitative content) |

###### Eassom et al. 2014

| Eassom et al. 2014 | |
| --- | --- |
| study | Eassom, E., Giacco, D., Dirik, A., & Priebe, S. (2014). Implementing family involvement in the treatment of patients with psychosis: a systematic review of facilitating and hindering factors. BMJ Open, 4(10). https://doi.org/10.1136/bmjopen-2014-006108 |
| design | Qualitative systematic review, all study designs, narrative synthesis  authors background: Social and Community Psychiatry  investigators participative? no |
| objective | To synthesize the evidence on implementing family involvement in the treatment of patients with psychosis with a focus on barriers, problems and facilitating factors. |
| search period | 20140601 |
| participants | psychiatric disorder: psychosis  caregiver type: families (or other unpaid carers) |
| interventions | Family involvement = any process allowing health professionals, families and patients to actively collaborate in treatment, such as in making joint treatment decisions; intervention involved tripartite communication between health professionals (any), families (unpaid carers) and adult patients  Exclusion criteria:   - studies not reporting clear information on how families were involved in treatment were excluded - studies into general experiences, opinions, satisfaction or needs, unless they related to a clearly described specific involvement in treatment - studies focused exclusively on professional–family communication, family–family communication or multiple-family groups |
| controls | Not applicable. |
| outcomes/ purpose | Barriers, problems and/or facilitating factors in involving families in treatment. |
| body of evidence | 43 studies; 588 professionals, 321 patients and 276 family members or families  publication years: 1978 to 2013  countries: 50% UK, others Finland, USA, Italy, Australia, Canada, Germany, India, Ireland, New Zealand, Spain, Greece, Portugal  quality rating of the included studies: not assessed. |
| AMSTAR2 rating | - 1/3 critical items not met - no list of excluded studies - very limited applicability of AMSTAR2 for qualitative reviews; no clear recommendations and tools for quality rating for qualitative studies |
| results | Descriptive results   - scientists background: not reported - participants details: not reported - interventions details   - duration and frequency: not reported   - setting: in- and outpatient, specialised service, partly not reported   - delivery format: not reported   - type: mainly psychosocial and family psychoeducation; but also Open Dialogue approaches (n=6), Systemic Psychotherapy (n=3) and Behavioural Therapy programme (n=1)   - personnel: not reported   - follow-up (range): not reported |
|  | Quantitative results  None. |
|  | Qualitative results  Barriers, problems and facilitating factors related to family work (see figure 2)   1. Context    1. Practical need associated with family work       1. Managing workload, practical burdens and balancing clinical responsibilities       2. Poor allowance of time from service       3. Lack of structure & standardises systems to support family work       4. Logistics (out of hours & practical requirements not facilitated) 🡪 top down support & strategic solutions 🡪 logistics (flexibility with appointments)    2. Personal & training need       1. Problems with access to supervision       2. Lack of skills or confidence 🡪 supervision & ongoing support    3. System-wide approaches & attitudes to family work       1. Organisational culture & paradigms       2. Unsupportive attitudes of managers       3. Lacking shared team commitment to family work       4. Anti-family work attitudes amongst staff       5. Difficulties with referrals & finding "appropriate“clients 🡪 organisation prioritisation & shared culture of family work 🡪 promotion of family work (among colleagues & within the organisation) 🡪 collaboration – multidisciplinary support 🡪 personal motivation & enthusiams 2. Engagement    1. Reservations about involving families       1. Issues with the nature of family involvement       2. Fear of worsening the situation 🡪 supervision & ongoing support    2. Problems with engaging families       1. Problems with engaging families to become involved       2. Problems with engagement during treatment 🡪 critical period of engagement with services 3. Delivery    1. Working with complex needs       1. Difficulties working with complex needs (family conflict, patient symptoms) 🡪 professional qualities, experiences & skills 🡪 professional relinquishing control    2. Working relationships between families & professionals       1. Lack of joint working during sessions       2. Professionals not applying systemic thinking effectively       3. Families experiencing approach as negative or critical       4. Lack of staff continuity 🡪 equal partnership & active collaboration with families 🡪 professional interpersonal qualities (non-blaming approach, empathising) 🡪 professional communication 🡪 continuity of car    3. Individualisation within the approach       1. Approach not considerate of individual needs or cultural differences 🡪 having a clear structure, allowing for flexibility |
|  | Limitations  use of appraisal tools in qualitative research remains contentious; often very small populations/case studies |
|  | Authors conclusion:  "Family work can only be implemented if this is considered a shared goal of all members of a clinical team and/or mental health service, including the leaders of the organisation. This may imply a change in the ethos and practices of clinical teams, as well as the establishment of working routines that facilitate family involvement approaches." |
| qualitative richness | High (review focuses on qualitative content or review was designed for qualitative content) |

###### Esteban et al. 2022

| Esteban et al. 2022 | |
| --- | --- |
| study | Esteban, J., Suárez‐Relinque, C., & Jiménez, T. I. (2022). Effects of family therapy for substance abuse: A systematic review of recent research. Family Process, 62(1), 49–73. https://doi.org/10.1111/famp.12841 |
| design | Systematic review, all study designs, narrative synthesis  authors background: Social and Human Sciences  investigators participative? no |
| objective | To review outcomes-focused evidence published in the last decade on sytemic family interventions for substance abuse in adolescent and adult samples. |
| search period | 2010–202101 |
| participants | psychiatric disorder: substance abuse (note: partly children/adolescents)  caregiver type: mother, father, partner, child  exclusion criteria: samples of relatives of people who have a substance use-related problem |
| interventions | family interventions with systemic approach  exclusion: multicomponent program, |
| controls | Not restricted. |
| outcomes/ purpose | patient outcomes: reduced substance abuse (degree of drug use, intention to use, readiness to change), mental health, behavior problems, Behavior disorder, Delincuency, Crimes  caregiver outcomes: family functioning |
| body of evidence | 18 studies (13 adolescent sample, 5 adult sample), 15 RCTs, 1 Quasi-experimental trial, 1 Single-case trial, 1 Pilot trial  n= 3200  publication years: 2010 to January 2021  countries: USA, Spain, Netherlands, Germany, Europe, Chile  quality rating of the included studies: Methodological Quality Rating Scale (MQRS, 0 = lowest, 16 = highest) ranging from 8 to 16. |
| AMSTAR2 rating | - 4/5 critical items not met - No protocol, no list of excluded studies, no risk of bias assessment, RoB not mentioned in discussion |
| results | Descriptive results   - scientists background: not reported - participants details   - disorder type: substance use disorder   - disorder onset/first treatment contact: not reported   - caregiver type: not reported   - recruitment: not reported   - mean age (range): 15,36 to 36,79 (excluded Single-case trial with N = 1, Age: 55)   - percentage women (range): 11% to 100% - interventions details   - duration and frequency: 4 to 24 sessions (most commonly 12 sessions between 3 and 4 months)   - setting: „real-world“clinical settings“; 5x outpatient, others not reported   - delivery format: individual, group, family, multi-family   - type: Multidimensional Family Therapy (MDFT, 7/18), Brief Strategic Family Therapy (BSFT, 3/18), Ecologically-Based Family Therapy (EBFT, 3/18), Functional Family Therapy (FFT, 1/18), Culturally-Informed and Flexible Family Treatment for Adolescents (CIFFTA, 1/18), Behavioral Family Therapy (FBT, 2/18), and Multifamily Therapy (MFT, 1/18)   - components: not reported (interventions details not described by types)   - personnel: not reported   - follow-up (range): 1 to 24   - Control groups: Cognitive Behavioral Therapy (CBT, 3/18), Motivational Interviewing (MI, 1/18), psychoeducation (2/18), individual treatment (2/18), or Residential Treatment (RT, 1/13) |
|  | Quantitative results  (Adult patients AND adult caregivers only)  Substance abuse reduction   - FBT: Participants improved significantly as they reported a greater number of days abstinent. - MFT-RT: MFT-RT group benefited more as they reduced their substance abuse and daily methadone dosage more than the controls (individual counselling).   Family functioning   - Most studies showed benefits in family functioning for participants; but partly also for controls   attrition/adherence/drop-out/acceptability: not reported |
|  | Qualitative results  none |
|  | Limitations  small sample sizes, More adolescent than adult samples, Women represent only small percentage in most included studies, partly poor reporting, Many studies relied on self-reported measure of substance use, Many studies were conducted in United States which limits generalization to other countries and cultures, Many variations of FT included, No distinguishment by type of substance |
|  | Authors conclusion  "The empirical evidence accumulated in the last decade and reviewed in the present study indicates that the incorporation of family members in the treatment of substance abuse produces benefits in patients and the family functional system. Overall, the results of the present study are clearer for the adolescent sample and with cases of higher severity of substance abuse. Therefore, age and severity need to be considered as potential moderators of the effects of a TF and must be taken into account in treatment decisions with this population." |
| qualitative richness | None |

###### Fleming et al. 2020

| Fleming et al. 2020 | |
| --- | --- |
| study | Fleming, C., Brocque, R. L., & Healy, K. (2020). How are families included in the treatment of adults affected by eating disorders? A scoping review. International Journal of Eating Disorders, 54(3), 244–279. https://doi.org/10.1002/eat.23441 |
| design | scoping review, all study designs, narrative synthesis  authors background: School of Nursing, Midwifery, and Social Work  investigators participative? no |
| objective | To evaluate the recent evidence on the inclusion of families in treatment programs for adults with eating disorders. |
| search period | 2006-2020  The search period was chosen to ensure relevance to current practice as, prior to 2006, the dominant conceptual model regarded families with anorexia nervosa as dysfunctional systems rather than resources for recovery and, clinically, attempts to involve families in the treatment regime of adults were limited. |
| participants | psychiatric disorder: eating disorders (adults)  caregiver type: family |
| interventions | family inclusive treatment approaches/systematic inclusion of family members |
| controls | Not restricted. |
| outcomes/ purpose | Not applicable (focus on descriptive results). |
| body of evidence | 68 studies (50 clinical studies, 8 review papers, and 10 grey literature sources)  publication years: 2006 to 2020  countries: United Kingdom (17), United States (12), Canada, Italy, France, Germany, Spain, Sweden, The Netherlands, Iceland, Australia  quality rating of the included studies: not assessed (not required for scoping reviews) |
| AMSTAR2 rating | - 2/3 critical items not met: no protocol, no list of excluded studies - AMSTAR2 applicable to scoping reviews only to a limited extent |
| results | Descriptive results   - scientists background: not reported - participants details   - disorder type: eating disorders; (27/53 anorexia nervosa; 6 bulimia nervosa or binge-eating)   - disorder onset/first treatment contact: not reported - caregiver type: family (not reported in detail)   - recruitment: mostly specialist eating disorder treatment settings (30/50 clinical studies), 10 studies unspecified or from a general mental health setting   - mean age (range): 8 studies focussing on adult patients, 32 studies mixed age; range 18 –57 years   - percentage women (range): only 3 studies included male patients; caregivers "predominantly“females: 2 only women, 30 mixed but dominated by females, in 11 studies not reported - interventions details   - duration and frequency: not summarized, heterogen; preset and predetermined by service provider   - setting: 10 inpatient, 40 outpatient, 5 in- and outpatient   - individual or group; interactive workbook, DVD, web-interventions, workshops, co-joint sessions, mail/phone support   - models: Cognitive interpersonal maintenance model; CBT, Systemic and cognitive behavioral theory principles, Relational/Motivational adaptation of Multifamily Therapy Group treatment model, Brief Adlerian Psychodynamic Psychotherapy, Calgary Family Intervention Model, and Lifespan emotionfocused family therapy model, Family systems theory …   - components: psychoeducation, communication skills, recovery coaching, emotion coaching, counseling … - personnel: "mostly expert service providers and highly experienced staff" - follow-up (range): not summarized - Programs:   - Maudsley Model of Treatment for Adults with Anorexia Nervosa (MANTRA)   - Uniting couples (in the treatment of) anorexia nervosa (UCAN)   - Uniting couples in the treatment of eating disorders binge-eating disorder edition (UNITE-BED)   - emotion-focused family therapy (EFFT)   - Experienced caregivers helping others (ECHO)   - Family-based treatment (FBT)   - Cognitive Behavioral Therapy for Bulimia Nervosa (CBT-BN) |
|  | Quantitative results  limited number of effectiveness studies  "Despite having been conducted in clinical treatment settings, very little data was found on the aggregate clinical effects of family intervention for adult patients. 11 papers that included both patient and carer outcomes of a family intervention, 20 studies provided outcome data for carer participants only." |
|  | Qualitative results  See FIGURE 2 Thematic map of family inclusive treatment for adults affected by eating disorders  Themes:   - a) conceptualization (context, setting, population) - b) central process (known or supposed mechanism, activities/procedures, materials used/provided) - c) clinical practice (mode of delivery/features; number of sessions/durations, provider/background/expertise)   currents gaps/areas for development (exampled):   - to a) Identification and engagement of non-primary caregivers, Recognition and inclusion, Implementation a multicomponent, as-needed, matched care model with a standardized family consultation method - to b) Flexible involvement options including titrated services with low intensity options - to c) Titrated intervention options based on recognition of varying family capacity and patient choice |
|  | Limitations  Limited number of effectiveness studies, heterogeneity of research designs, large range of settings and instruments, absence of studies that evaluated long-term outcomes, dearth of research identified into FIT approaches with family of choice, rather than family of origin, or with remote family members included via modern technologies. |
|  | Authors conclusion  "Results confirmed that family members of adults were willing to be involved with eating disorder treatment services and appeared to respond to interventions of varying intensity and duration. The impact on individual patients, and effect on treatment outcomes, are yet to be established." |
| qualitative richness | Moderate (review contains explicit qualitative statements, even if this was not the primary focus of the study); see figure 2 and chapter 3.3 Synthesis of results. |

###### Frey et al. 2021

| Frey et al. 2021 | |
| --- | --- |
| study | Frey, L. M., Hunt, Q. A., Russon, J. M., & Diamond, G. (2021). Review of family‐based treatments from 2010 to 2019 for suicidal ideation and behavior. Journal of Marital and Family Therapy, 48(1), 154–177. https://doi.org/10.1111/jmft.12568 |
| design | systematic review, all study designs, narrative synthesis  authors background: Couple and Family Therapy Program, Marriage and Family Therapy Program, Human Development and Family Science, Marriage and Family, Counseling and Family Therapy  investigators participative? no |
| objective | To review the empirical evidence for couple and family interventions for suicidal ideation and behavior. |
| search period | 2010-2019 |
| participants | psychiatric disorder: suicide ideation and behavior  caregiver type: family |
| interventions | family- based treatments |
| controls | Not applicable. |
| outcomes/ purpose | patient outcomes: not predefined  caregiver outcomes: not predefined |
| body of evidence | 22 studies (16 RCT)  publication years: 2010 to 2019  countries: USA (17), Germany (1), Norway (2), Australia (1), not reported (1)  quality rating of the included studies: not assessed |
| AMSTAR2 rating | - 4/5 critical items not met - No protocol, no list of excludes studies, risk of bias not assessed and mentioned in discussion   This review does not fulfill the methodological requirements for systematic reviews. Given the focus on descriptive results this might be acceptable. |
| results | Descriptive results   - scientists background: not reported - participants details   - disorder type: Suicide ideation and behavior   - disorder onset/first treatment contact: not reported   - caregiver type: "family", not reported in detail   - recruitment: not reported   - mean age (range): adolescents patients (lowest cut- off 10-13, highest cut- off 17-19 years)   - percentage women (range): not reported - interventions details   - duration and frequency: not summarized   - setting: emergency, outpatient   - (a) individual sessions with the suicidal person, (b) family training sessions, (c) family psychoeducational sessions, and (d) conjoint family sessions (details see below)   - components: some type of crisis intervention (all studies); skill- building, coping, problem-solving   - personnel: not reported   - follow-up (range): not reported - Categories of components   - (a) individual sessions with the suicidal person (n=8)   - (b) family training sessions (n=7): "training skills needed to be in- home coaches for suicidal individuals or to repair family dynamics"; "family members often learn or review the same skills the suicidal persons learn in their individual sessions"; "learn additional skills targeting their specific role as a coach, such as facilitating healthy conflict resolution, improving family communication, and/or contingency management and behavior contracting", "to increase parent motivation to support patients and to decrease family- based barriers to treatment"   - (c) family psychoeducational sessions (n=2): lecture- and discussion- based, incl. Stress management   - (d) conjoint family sessions (sessions attended by both the suicidal person and the family member; n=10); partly on top of separate lessons - Categories of interventions   - based on clinical approach (e.g., CBT, DBT, systemic) and setting (e.g., emergency department). Rating by Southam-Gerow and Prinstein’s criteria for classifying evidence-based psychosocial interventions (Level 1 = highest, Level 5 = lowest).   - CBT + Parent Training (n=3) (Level 2)   - CBT Family— Emergency Setting (n=1) (Level 4)   - CBT + Systemic Principles— Emergency Setting (n=1) (Level 4)   - CBT + Systemic Principles (n=3) (Level 2)   - Dialectical- Behavior Therapy + Family Training (n=6) (Level 1)   - Systemic Principles (n=6) (Level 1)   - Psychoeducation (n=2) (Level 2) |
|  | Quantitative results  Suicide- related outcome measures + family- outcome measures: self-harm behavior (severity markers); family functioning  9/22 reported family- focused outcomes  "Fewer studies reported potential interaction variables for suicide- related and family- focused outcomes"  used instruments:   - ASQ- R, Adolescent Suicide Questionnaire- Revised; Ax, Assessment; BPD, Characteristics of borderline personality disorder; BSS, Beck Scale for Suicide Ideation; CBQ- 20, Conflict Behavior Questionnaire- 20; C- SSRS, Columbia- Suicide Severity Rating Scale; DISC- IV, National Institute of Mental Health Diagnostic Interview Schedule for Children Version IV; FAD, McMaster Family Assessment Device; GPACS, Goal- Corrected Partnership in Adolescence Coding System; HASS, Harkavy Asnis Suicide Scale; K- SADS, Kiddle Schedule for Affective Disorders and Schizophrenia— Patient Version; K- SADS- PL, Kiddle Schedule for Affective Disorders and Schizophrenia for School- Aged Children, Present and Lifetime Version; NSSI, Nonsuicidal self- injury; PBI, Parent bonding instrument; PCICS, Parent- Child Interactional Coding Scale; RSQ, Relationship Scale Questionnaire; SACA, Service Assessment for Children and Adolescents; SASII, Suicide Attempt Self- Injury Interview; SCID- I/P, Structured Clinical Interview for DSM- IV— Patient Version; SIQ, Suicide Ideation Questionnaire; SIQ- JR, Suicide Ideation Questionnaire— Junior Version; SRFF, Self- Report of Family Functioning   attrition/adherence/drop-out/acceptability: not reported |
|  | Qualitative results  None. |
|  | Limitations  Family-based interventions are nonexistent (in published research) for age groups outside of adolescence.  Family- outcome measures only reported if an interaction effect was tested in relation to treatment outcomes; no quality assessment of included studies. |
|  | Authors conclusion  "… we identified two well established intervention categories that met the highest standards for interventions and three probably efficacious intervention categories. All interventions found focused solely on suicide risk in adolescent populations" |
| qualitative richness | Low (review contains only little qualitative content, see chapter results/discussion/limitations …) |

###### Grácio et al. 2015

| Grácio et al. 2015 | |
| --- | --- |
| study | Grácio, J., Gonçalves‐Pereira, M., & Leff, J. (2015). What do We Know about Family Interventions for Psychosis at the Process Level? A Systematic Review. Family Process, 55(1), 79–90. https://doi.org/10.1111/famp.12155 |
| design | Qualitative systematic review, all study designs, narrative synthesis  authors background: Psychiatry, mental health  investigators participative? no |
| objective | to identify process analyses of the interventions, which could allow the identification of key elements and drawing of conclusions about the relationships between process and effective outcomes |
| search period | December 2013 |
| participants | psychiatric disorder: psychosis  caregiver type: not specified |
| interventions | family interventions |
| controls | Not applicable |
| outcomes/ purpose | Qualitative: process-related information (therapeutic alliance, group dynamics, themes, core techniques, mediating variables, mechanisms of change) |
| body of evidence | 22 studies  publication years: 1988 to 2012   - 1 Study Comprehensively Examining the Process of an Intervention - 11 Studies on Participants’ Opinions about the Intervention Received - 4 studies Comparative Studies Individualizing Process-related Variables - 6 studies Studies on the Exploration of Mediating Variables Related to FIP Outcome   countries: not reported  quality rating of the included studies: not assessed |
| AMSTAR2 rating | - 2/3 critical items not met: no protocol, no list of excluded studies - Comment: very limited applicability of AMSTAR2 for qualitative reviews |
| results | Descriptive results   - scientists background: not reported - participants details: not reported - interventions details: not reported - quality rating of the included studies: not assessed |
|  | Quantitative results  Not applicable. |
|  | Qualitative results  Core elements identified:   - Participants' opinion: Therapeutic alliance, support, education - Comparative studies: therapeutic alliance, education, coping skills training   Common therapeutic factors:   - therapeutic alliance, support, and the opportunity for sharing (regardless of specific techniques)   Role of information-giving and education   - Information alone can be effective in decreasing the stress in the family - Education can be effective in reframing relatives’ views about patients’ locus of control and agency - Reframing emerges as a mediating variable in lowering or dealing with expressed emotion . Therefore, education becomes a major predictor of relapse prevention - Education may also play a part in engagement and emotional support. It is likely that relatives perceive education as meaning that professionals are highly committed to helping them and appreciate their role as caregiver. |
|  | Limitations  no clear recommendations and tools for quality rating for qualitative studies and qualitative reviews |
|  | Authors conclusion  „We found that being in a therapeutically supportive relationship, followed by education about the illness, and later coping skills training, look to be the major active ingredient at different levels of intervention. FIP must be pragmatic programs, delivered by professionals trained in evaluating families’ needs that have the expertise to deliver different techniques moment-by-moment as they are necessary." |
| qualitative richness | High (review focuses on qualitative content or review was designed for qualitative content) |

###### Hansen et al. 2022

| Hansen et al. 2022 | |
| --- | --- |
| study | Hansen N. H., Bjerrekær, L., Pallesen, K. J., Juul, L. & Fjorback, L. O. (2022). The effect of mental health interventions on psychological distress for informal caregivers of people with mental illness: A systematic review and meta-analysis. Frontiers in Psychiatry, 13. https://doi.org/10.3389/fpsyt.2022.949066 |
| design | systematic review of RCTs, meta-analysis  authors background: Clinical Medicine, Mindfulness  investigators participative? no |
| objective | (1) to investigate which intervention modality showed the most effect on decreasing caregiver psychological distress,  (2) to investigate which type of delivery format (group or individual) showed the most effect,  (3) to investigate which intervention components … showed the most effect,  (4) to investigate the effect … when grouped into (a) dementia and (b) severe mental disorders |
| search period | 201906 |
| participants | disorder: mental illness (incl. dementia) (exclusion: mixed samples of both physical and mental illness)  caregiver type: a spouse, child, partner, parent, and/or other members of the family |
| interventions | mental health interventions  Exclusion: intervention given in dyads (caregiver + care receiver), |
| controls | waitlist, treatment as usual, or active control. |
| outcomes/ purpose | patient outcomes: none  caregiver outcomes: psychological distress (primary outcome)  depression, anxiety, stress, satisfaction with life, emotion regulation |
| body of evidence | 44 RCTs (31 in meta-analysis, n=1899; 15 on severe mental illness)  countries: not reported  quality rating of the included studies: poor (all) |
| AMSTAR2 rating | - 1/7 critical items not met - Publication bias not assessed |
| results | Descriptive results   - scientists background: not reported - participants details: not summarized - interventions details: not summarized - assessed outcome measures: depression (CES-D; DASS), anxiety (BAI, STAI, HADS), stress (PSS, RMBPC), subjective burden (ZCBS, ZBI, FBIS, SBAS), quality of life (GHQ, WHOQOL-BREF, SF-36, AQoL-8D, GHQ-28), psychological distress (NPI, HSCL, SCL-90, CISR) - quality rating of the included studies: all low (Cochrane RoB) |
|  | Quantitative results  Patient outcomes: not reported  Caregiver outcomes: "31/44 with sig. effect"   - "An effect is seen in psychoeducation, psychosocial, multicomponent, cognitive behavioral therapy, and mindfulness-based and support group interventions. - The no-effect studies are characterized by short duration, having a long-term follow-up period, or being a technology-based intervention."   Meta-analyses on Psychological distress   - interventions vs. control (all types): SMD −0.32 (95% CI −0.53 to −0.11); I2=78%; 31 RCTs, n=1899 - interventions vs. active control: SMD −0.24 (95% CI: −0.48 to 0); I2=41%; 10 RCTs, n=484 - severe mental illness (vs. all types of controls): SMD – 0,58 (-1,19 to -0,16); I2=91; 15 RCTs, n=823 - dementia/Alzheimer: SMD -0,20 (-0,34 to -0,05); I2=13%; 17 RCTs; n=1049 - individual format: SMD -0,38 (-0,64 to -0,11); I2=68%; 12 RCTs, n=828 - group format: SMD -0,43 (-0,80 to -0,07); I2=86%; 19 RCTs, n=944 - manualized interventions ≥ 8 weeks: SMD −0.38 (95% CI −0.56 to −0.2); I2=47%; 20 RCTs, n=1049 - non-manualized interventions <8 weeks: SMD −0.31 (95% CI: −0.79 to 0.17); I^2^= 96%; 21 RCTs, n=728   attrition/adherence/drop-out/acceptability: not reported |
|  | Qualitative results  none |
|  | Limitations   - small sample sizes, substantial statistical and PICO heterogeneity - because of the different and inconsistent component descriptions, we were not able to conduct subgroup analyses on intervention modality (components) - Different self-report questionnaires to measure psychological distress, - Risk of bias |
|  | Authors conclusion  „The evidence supports that several interventions improve the mental health of caregivers. Manualized interventions ≥ 8 weeks with active participation are most effective." |
| qualitative richness | Low (review contains only little qualitative content, see subgroup analyses) |

###### Henken et al. 2007

| Henken et al. 2007 | |
| --- | --- |
| study | Henken T, Huibers MJ, Churchill R, Restifo KK, Roelofs JJ. Family therapy for depression. Cochrane Database of Systematic Reviews 2007, Issue 3. Art. No.: CD006728. DOI: 10.1002/14651858.CD006728. |
| design | systematic review, controlled trials, narrative synthesis  authors background: Cochrane Common Mental Disorders Group; Psychological Science; Psychiatry; Community Based Medicine; Medical, Clinical and Experimental Psychology  investigators participative? no |
| objective | To assess the efficacy of family therapy for depression |
| search period | 200510 |
| participants | psychiatric disorder: depression  caregiver type: family  inclusion criteria: therapy consists of different phases (assessment, psychoeducation, improving functioning, cognitive, behavioural, systemic approaches, feedback, closure; delivered by clinician or therapist; sessions attended by patient and caregivers; minimum six sessions, duration of at least one hour |
| interventions | Family therapy |
| controls | No or alternative intervention |
| outcomes/ purpose | patient outcomes: depression symptomatology; other symptoms (eg hopelessness, suicidal ideation, anxiety); behaviour and functioning; other outcomes (eg social support, expectancies of treatment, quality of life)  caregiver/family outcomes: family functioning |
| body of evidence | 6 RCTs, n= 519  publication years: 1985 to 2004  countries: USA (5), Australia (1)  quality rating of the included studies: 3x high, 3x low quality |
| AMSTAR2 rating | all critical items met |
| results | Descriptive results   - scientists background: not reported - participants details   - disorder type: depression, in some studies miscellaneous or depression as co-morbidity   - disorder onset/first treatment contact   - caregiver type: not reported   - recruitment: not reported   - mean age: not summarized; children (1), adolescents (2), adults (3)   - percentage women: not reported, 1 trial on mothers - interventions details   - duration and frequency: 8-18 weeks; 8-15 sessions   - setting: 4x outpatient, 2x inpatient   - delivery format: miscellaneous, individual/multifamily, sessions, workshops   - models: systemic behaviour family therapy (SBFT), attachment-based family therapy (ABFT), psychoeducational inpatient family intervention, family therapy, pharmacotherapy, psychoeducational group therapy, behavioural family intervention (BFI), cognitive behavioural family intervention (CBFI), family bereavement program   - components: psychoeducation, problem solving, communication roles, affective responsiveness, affective involvement, and behaviour control; didactic teaching, therapist-guided practice, roleplay, feedback, and coaching to teach behavioural principles and and techniques to parents, cognitive therapy components; family grief workshop   - personnel: not reported   - follow-up (range): 0 to 28 months |
|  | Quantitative results  No effect sizes reported; narrative description for each study separately  „positive findings of some studies … on decreasing depression and on increasing family functioning"  Patient outcomes:   - Miscellaneous depression rating scales   Caregiver/family outcomes   - Self-Report of Family Functioning (SRFF) - Family Attitude Inventory - Goals of Inpatient Family Intervention Rating Scale - Role Performance Treatment Scale - Family cohesion: 7 items of the FES - Stable positive events: GLES-C and PDEL - Family coping by reframing: F-COPES - miscellaneous parental outcome measures   attrition/adherence/drop-out/acceptability: not reported |
|  | Qualitative results  None. |
|  | Limitations  very heterogeneous in terms of interventions, participants, and measuring instruments; not only depression; not only adult patients |
|  | Authors conclusion  "current evidence base is too heterogeneous and sparse to draw conclusions on the overall effectiveness of family therapy in the treatment of depression " |
| qualitative richness | None (review contains no qualitative statements) |

###### Higgins et al. 2020

| Higgins et al. 2020 | |
| --- | --- |
| study | Higgins, A., Murphy, R., Barry, J., Eustace-Cook, J., Monahan, M., Kroll, T., Hevey, D., Doyle, L. & Gibbons, P. (2020). Scoping review of factors influencing the implementation of group psychoeducational initiatives for people experiencing mental health difficulties and their families. Journal Of Mental Health, 31(6), 859–872. https://doi.org/10.1080/09638237.2020.1714002 |
| design | scoping review, all study designs, qualitative synthesis  authors background: Nursing and Midwifery, Psychology, Mental Health Service  investigators participative? no |
| objective | to synthesise the peer-reviewed literature on barriers and enablers influencing the implementation of group psychoeducation in adult mental health services |
| search period | 2018 |
| participants | psychiatric disorder: mental health problems  caregiver type: family |
| interventions | Psychoeducational programs for adults (>18) with mental health problems and/or their families; conducted in a group format in the mental health services |
| controls | Not applicable. |
| outcomes/ purpose | evidence on barriers and enablers to the implementation and sustainability of the educational initiatives; and/or information on the strategies for overcoming such barriers |
| body of evidence | 8 studies  publication years: 2009 to 2016  countries: USA (4), United Kingdom (2), Sweden (1), Australia (1)  quality rating of the included studies: 6x good, 2x fair |
| AMSTAR2 rating | - 1/3 critical items not met (quality rating and discussion realised, although this is not required for scoping reviews) - No list of excluded studies - AMSTAR2 applicable to scoping reviews only to a limited extent |
| results | Descriptive results   - scientists background: not reported - participants details   - disorder type: bipolar disorder, schizophrenia, first episode of psychosis, serious mental illness, major depressive disorder, PTSD   - disorder onset/first treatment contact: not reported   - caregiver type:   - recruitment: not reported   - mean age (range): not reported   - percentage women (range): not reported - interventions details:   - duration and frequency: not summarized   - setting: not summarized   - individual/group/couples/family: not summarized   - components: education   - personnel: 4x clinicians, 1x co-facilitated clinicians+peers, 3x not reported   - follow-up (range): not reported |
|  | Quantitative results  Outcome reporting: 2x both family members and service patients; 3 service-patients only, 3 family-members only |
|  | Qualitative results   \| **Levels** \| **Barriers** \| **Enablers** \| \| --- \| --- \| --- \| \| **Participant** \| Service patient   - Mood - Familial dynamics - Concerns about confidentiality - Stigma - Competing demands   Family member   - Discomfort with group format - Familial dynamics - Stigma - Competing demands \| Service patient and family members   - Format of intervention that provided opportunity to share experiences, learn from each other and engage socially \| \| **Provider** \| Clinical facilitators   - Challenges negotiating workload - Skills deficits - Lack of support - Perception of benefit to attendees   Peer facilitators   - Financial reimbursement - Lack of support \| - Positive attitudes to developing expertise in recovery oriented and consumer oriented approaches - Presence of a peer facilitator - Facilitator skill \| \| **Intervention** \| - Content not tailored to needs of group - Pressures from group format - Style and skill of facilitators - Programme guidelines too restrictive - Recruitment approach - Timing & Location impacting on accessibility \| - Content of programme - Written handouts - Delivery in spaces within the community - Recruitment through ongoing engagement \| \| **Organisation** \| - Leadership deficits across the mental health system state/service/centre/programme level - Planning deficits - Managerial support deficits - Resource deficits (securing long terms funding and venue) - Resistance within organisation \| - Leadership across the mental health system state/service/centre/programme level) that championed the intervention - Strong culture of innovation - Allocation of extra personnel - Managerial support - Clear communication between stakeholders \| \| **Structural** \| - Cost and reliability of public transport \|  \| |
|  | Limitations  some degree of variability in relation to the factors identified, small sample sizes, the convenience nature of the sampling, diverse range of settings, heterogeneity among the target audience for the intervention (service patients/family) as well as combining peer or professionally led interventions, subjective self-report measures, collecting data prior to or immediately post intervention (lack of long-term sustainability) |
|  | Authors conclusion  "Implementing evidence-based group psychoeducation interventions is a complex process as a broad range of factors across multiple levels affect implementation outcomes … Without this form of evidence, it is unlikely that interventions like group psychoeducation will be added and embedded as a treatment option across all mental health services" |
| qualitative richness | High (review focuses on qualitative content or review was designed for qualitative content) |

###### Krysinska et al. 2021

| Krysinska et al. 2021 | |
| --- | --- |
| study | Krysinska, K., Andriessen, K., Ozols, I., Reifels, L., Robinson, J. & Pirkis, J. (2022). Effectiveness of Psychosocial Interventions for Family Members and Other Informal Support Persons of Individuals Who Have Made a Suicide Attempt. Crisis, 43(3), 245–260. https://doi.org/10.1027/0227-5910/a000776 |
| design | systemativ review, all study designs, narrative synthesis  authors background: School of Population and Global Health, Mental Health at Work  investigators participative? no |
| objective | - to identify and synthesize available studies on the effectiveness of psychosocial interventions for informal carers after a suicide attempt |
| search period | 202004 |
| participants | psychiatric disorder: suicide attempt (exclusion: suicidal ideation or self-harm without any suicidal intent)  caregiver type: family members and other informal support persons |
| interventions | interventions designed specifically for informal carers and interventions designed for persons who have made a suicide attempt, and which involved their informal carers |
| controls | Not restricted |
| outcomes/ purpose | Not predefined  Exclusion: no outcome reporting for caregivers |
| body of evidence | 7 studies (3 RCTs, 1 NRSI, 3 pre/post cohorts); of this 3 for informal caregivers (all non-RCTs)  publication years: 1996 to 2018  countries: UK (2), USA (2), South Korea (1), Sweden (1), Taiwan (1)  quality rating of the included studies: weak (6), moderate (1) |
| AMSTAR2 rating | all critical items met |
| results | Descriptive results   - scientists background: not reported - participants details: not summarized/not reported for all studies   - interventions for suicide attempter: 4/4 targeted young people 🡪 parents as caregivers - interventions details: not summarized   - interventions for caregivers: Family Connections program (dialectical behav. therapy); education, suicide prevention plan; communication, counselling |
|  | Quantitative results  No synapsis, reported for each study separately  Studies for caregivers (n=3):   - mixed results in terms of carers’ burden and distress and family functioning - „Only one of the three studies [on caregiver interventions] measured outcomes related to carers’ mental health, quality of life, and engagement in the intervention, and this study showed promising results"   Studies for suicide attempter (n=4):   - "mixed results in terms of carers’ mental health and family functioning, although carers were involved and satisfied with the intervention“„None of the four studies measured carers’ burden or distress or their quality of life" |
|  | Qualitative results  none |
|  | Limitations  small number of studies, heterogeneity of the outcomes reported, weak study quality; no data on effectiveness of interventions for siblings and informal carers beyond the family |
|  | Authors conclusion  „Psychosocial interventions designed specifically for informal carers seem to lower their burden of care and improve the ability and willingness to care for a suicidal family member " |
| qualitative richness | None (review contains no qualitative statements) |

###### Lohrasbi 2022

| Lohrasbi 2022 | |
| --- | --- |
| study | Lohrasbi, F., Alavi, M., Akbari, M. & Maghsoudi, J. (2023). Promoting Psychosocial Health of Family Caregivers of Patients with Chronic Mental Disorders: A Review of Challenges and Strategies. Chonnam Medical Journal, 59(1), 31. https://doi.org/10.4068/cmj.2023.59.1.31 |
| design | systematic review, all study designs, narrative synthesis  authors background: Nursing & Midwifery Care Research  investigators participative? no |
| objective | to investigate the social and mental health challenges of family caregivers of patients with chronic mental disorders and strategies which can be helpful in this regard |
| search period | 2000-2021 |
| participants | psychiatric disorder: chronic mental disorders  caregiver type: not specified |
| interventions | interventions to promote psychosocial health of caregivers |
| controls | Not predefined |
| outcomes/ purpose | patient outcomes: none  caregiver outcomes: challenges, needs, and strategies of family caregivers |
| body of evidence | 19 studies (13 RCTs)  publication years: 2004 to 2021  countries: not reported  quality rating of the included studies: not assessed |
| AMSTAR2 rating | - 4/5 critical items not met - No protocol, no bias assessment and discussion, no list of excluded studies - Review does not fulfil methodological requirements for systematic reviews. |
| results | Descriptive results   - scientists background: not reported - participants details: not reported - interventions details: not summarized |
|  | Quantitative results  No effect sizes reported.   1. Empowerment programs  - psycho-educational most important methods 🡪 can be effective in reducing individual dimensions of stigma, burden of care, and also in promoting family tolerance, caregivers quality of life, caregivers’ emotional regulation and improvement of psychological symptoms. - web-based applications or (face-to-face)  1. Peer support programs  - improve psychosocial health in family caregivers of people with CMD, peer support = “help and support that people with experience of caring for a mental illness or disability have learned and can pass on these experiences to others” |
|  | Qualitative results   - None. |
|  | Limitations   - Very superficial synopsis. |
|  | Authors conclusion   - "Using a collaborative approach, mental health service providers and government systems can help improve the psychosocial health of caregivers. The related managers and policymakers can reduce the emotional and psychological burden of families and promote their psychosocial health through developing a comprehensive program including practical objectives and strategies" |
| qualitative richness | - None (review contains no qualitative statements) |

###### Ma et al. 2017

| Ma et al. 2017 | |
| --- | --- |
| study | Ma C. F., Chien, W. T., & Bressington, D. T. (2017). Family intervention for caregivers of people with recent-onset psychosis: A systematic review and meta-analysis. Early Intervention in Psychiatry, 12(4), 535–560. https://doi.org/10.1111/eip.12494 |
| design | systematic review, RCTs only, meta-analysis  authors background: hospital/university  investigators participative? no |
| objective | to critically examine the effects of family intervention on recent-onset psychosis by systematically evaluating caregiver-related outcomes and comparing the effects resulting from different types of family intervention |
| search period | 201606 |
| participants | psychiatric disorder: recent-onset psychosis  caregiver type: first- and second-degree relatives  inclusion criteria: RCTs reporting effectiveness of family interventions on caregiver outcome  exclusion criteria: friends and maids |
| interventions | Any family intervention targeting the needs of family members: psychosocial interventions (psychoeducation, mutual support group and bibliotherapy), psychotherapies (mindfulness, family therapy and narrative therapy) and other structured psychological interventions |
| controls | Usual psychiatric care and multiple comparison treatment groups |
| outcomes/ purpose | patient outcomes: not assessed  caregiver (family) outcomes: family’s expressed emotion, psychological condition and/or distress, emotional climate, care burden or strain, functioning, caregiving experience, coping skills and problem-solving ability  Secondary outcomes: supportive service utilization and/or satisfaction, perceived social support and general health condition |
| body of evidence | 12 RCTs (9 in meta-analysis), n=1644 (698 patients, 946 carers)  publication years: 2004 to 2016  countries: UK, Spain, Australia, Hong Kong  quality rating of the included studies: overall low or unclear; no blinding |
| AMSTAR2 rating | - 1/7 critical items not met - Risk of bias not considered in discussion |
| results | Descriptive results   - scientists background: not reported - participants details   - disorder type: first-episode psychosis, early-onset psychosis, recent-onset psychosis or schizophrenia   - disorder onset/first treatment contact: <=5 years   - caregiver type: mostly "living with patients", parents or spouses   - recruitment: not reported   - mean age (range): patients 16.4 to 28.8 years, carers 40.6 to 45.2 years   - percentage women (range): about 1/3 of patients and 2/3 of carers - interventions details   - duration and frequency: 22 weeks (SD = 12.5; range = 5-36 weeks); average number of sessions was 11.1 (SD = 4.60; range = 5-18 sessions, number of contact hours 20.5 (SD = 10.3; range = 9-36 hours)   - setting: community treatment setting   - delivery format: 4 RCTs both patients and carers together, 1 same intervention separately, 4 partly together, 7 carers only   - components: main approaches: mutual support groups and psychoeducation; others: adopted self-help or clinician-supported bibliotherapy; no psychotherapy   - personnel: not reported   - follow-up (range): mean follow-up 11.8 months (SD = 10.1; range = 1-36 months) - caregiver outcomes (used instruments): care burden (FBIS), family functioning (FAD), formal support services utilization (FSSI), Family perceptions of social support (SSQ6), Caregiving experience (ECI), Social problemsolving ability (SPSI-R:S), Service satisfaction (VSSS- 32), Psychological distress (K10), Expressed emotion (FQ-CC), Carers’ general health SF-12-PH/MH |
|  | Quantitative results  patient outcomes: not assessed  caregiver outcomes (vs. usual care):   - care burden (FBIS) short-term: SMD −0.65, 95% CI = −0.87 to −0.42), 4 RCTs, n=312 - care burden medium-term: SMD −0.76, 95% CI = −1.20 to −0.32), 3 RCTs, n=256 - care burden long-term (>6-12 m): SMD −0.97, 95% CI = −1.49 to −0.46), 3 RCTs, n=264 - family functioning (FAD): <6 month n.s.; 6-12 month SMD 0,82 (0,45; 1,18); 3 RCTs, n=287 - formal support services utilization (FSSI): n.s. until 12 month, >12-24m SMD -0,57 (-0,8; -0,34), 3 RCTs, n=337   subgroup analysis: mutual support more effective than psychoeducation  attrition/adherence/drop-out/acceptability: not reported   - attrition rate 13% (median = 9.8%; range = 5.2%-42.2%) - average intervention dropout rate 10% (median = 10.6%; range = 4.5%-17.5%). |
|  | Qualitative results  None |
|  | Limitations  58% of RCTs from Hongkong, 1 reviewer lead author of some of the included studies; lack of studies, small population |
|  | Authors conclusion  „family intervention is effective for caregivers of patients with recent-onset psychosis, especially on care burden where the positive effects are enhanced over time." |
| qualitative richness | Low (review contains only little qualitative content, see chapter discussion and implications for future research) |

###### Ma et al. 2019

| Ma et al. 2019 | |
| --- | --- |
| study | Ma, C. F., Chan, S. K. W., Chien, W. T., Bressington, D., Mui, E. Y. W., Lee, E. H. M. & Chen, E. Y. H. (2019). Cognitive behavioural family intervention for people diagnosed with severe mental illness and their families: A systematic review and meta‐analysis of randomized controlled trials. Journal Of Psychiatric And Mental Health Nursing, 27(2), 128–139. https://doi.org/10.1111/jpm.12567 |
| design | systematic review of RCTs, meta-analysis  authors background: Psychiatry, Brain and Cognitive Sciences, School of Nursing, Caritas  investigators participative? no |
| objective | to systematically examine the trial evidence of the effective‐ ness of CBFI versus treatment as usual (TAU) on improving the outcomes of people diagnosed with SMI and their families |
| search period | 201808 |
| participants | psychiatric disorder: severe mental illness: psychotic disorders, such as schizophrenia, schizoaffective disorder and bipolar affective; exclusion of studies with > 30% of service patients co‐ morbid with neurocognitive disorder and substance‐induced psychotic disorder  caregiver type: families/all adult relatives; exclusion of studies with > 30% of the family subjects are non‐family primary caregivers |
| interventions | - Cognitive behavioural family intervention (CBFI) as a brief psychosocial intervention - any CBT‐based family interventions delivered in inpatient, community or rehabilitation centre with a minimum of 4 sessions involving family members with/without service patients - exclusion: combination with other therapies |
| controls | any |
| outcomes/ purpose | all usable service patient and family outcome data collected by validated instruments |
| body of evidence | 4 RCTs, n=524 (of this n=287 caregivers)  countries: 2x Pakistan, Spain, UK  quality rating of the included studies: low/unclear risk of bias, but no blinding |
| AMSTAR2 rating | - 2/7 critical items not met - No list of excluded studies, risk of bias not mentioned in discussion |
| results | Descriptive results   - scientists background: not reported - participants details   - disorder type: psychotic disorder (schizophrenia, schizoaffective disorder, bipolar affective d.)   - disorder onset/first treatment contact: 4.7 years to 12.7 years   - caregiver type: family   - recruitment: not reported   - mean age (range): 30.2 to 36.0 (service patients; caregivers not reported)   - percentage women (range): not reported - interventions details   - duration: mean 16.5 weeks (SD = 7.53; range = 8–24 weeks)   - sessions: mean12 (SD = 3.67; range = 7–16),   - setting: 2x inpatient, 2x outpatient   - components: CBT sessions, workbooks, homework, home visits   - personnel: not reported   - follow-up (range): not reported |
|  | Quantitative results  Patient outcomes   - Meta-analysis (2 studies)   - overall positive symptoms MD = −7.39 (95% CI = −13.86 to −0.92), ; I2 = 96%   - delusions MD = −7.48; 95% CI = −12.38 to −2.58; I2=93%   - hallucinations: MD (MD = −10.38; 95% CI = −21.45 to 0.70); I2=96%   - overall negative symptoms (MD = −4.35; 95% CI = −5.62 to −3.08); I2=31%   - general psychopathology (MD = −10.26; 95% CI = −17.41 to −3.10); I2=94%   - insight MD = 5.38; 95% CI = −0.99 to 11.75); I2=98% - not meta-analysed (3^rd^ study): number of relapses, number of admissions, duration of relapse, number of relapses without admission, first relapse rate   caregiver outcomes:   - reported in 1/4 studies - assessed outcomes (all significant):   - depression, anxiety   - coping ability (by Maladjustment Scale)   - level of self‐esteem (by Rosenberg Self‐Esteem Scale)   attrition/adherence/drop-out/acceptability: not reported |
|  | Qualitative results  Expectations towards caregivers: "family caregivers could share information, ask questions, complete homework and collaborate closely with the therapists“… "teaching how to improve cognitive distortions, automatic thoughts and coping skills" |
|  | Limitations  Few number of studies, only 2 studies in meta-analysis |
|  | Authors conclusion  "The findings reveal that CBFI is superior to TAU in treating positive and negative symptoms immediately following the intervention " |
| qualitative richness | Low (review contains only little qualitative content, see chapter results/discussion/limitations …) |

###### MacLeod et al. 2011

| Macleod et al. 2011 | |
| --- | --- |
| study | Macleod, S. H., Elliott, L. & Brown, R. What support can community mental health nurses deliver to carers of people diagnosed with schizophrenia? Findings from a review of the literature. Int J Nurs Stud. 2011 Jan;48(1):100-20. doi: 10.1016/j.ijnurstu.2010.09.005. Epub 2010 Oct 16. PMID: 20956000. |
| design | Qualitative systematic review of comparative studies, narrative synthesis  authors background: Nursing and Midwifery  investigators participative? no |
| objective | to determine the nature of support that mental health nurses could deliver to carers of people diagnosed with schizophrenia. The aim was to identify approaches that could be delivered within community practice to reduce burden and increase knowledge, mental health and coping. |
| search period | 2008 |
| participants | psychiatric disorder: schizophrenia  caregiver type: not specified |
| interventions | psychosocial, education, day care, mutual support, community, mental health services |
| controls | Not predefined |
| outcomes/ purpose | patient outcomes: not predefined  caregiver outcomes: knowledge, coping and managing, mental and physical health. |
| body of evidence | 68 studies  publication years: 1980 to 20008  countries: not summarized, all continents; many from UK, US, China  quality rating of the included studies: assessed, but not reported in detail: "varying in quality" |
| AMSTAR2 rating | - 4/5 critical items not met - No protocol, not list of excluded studies, quality rating not reported and not mentioned in discussion |
| results | Descriptive results   - scientists background: not reported - participants details: not summarized - interventions details   - duration and frequency: not summarized   - setting: not summarized   - delivery format: not summarized   - type: education, supportive family education, family interventions, community support services, mutual support groups, day care services   - personnel: 18% delivered by nurses; 24% nurses included   - follow-up (range): not reported |
|  | Quantitative results  for each study reported separately; summarization:   - education programmes improve knowledge about schizophrenia, however there is little evidence that this addresses more substantive areas of caring such as improved coping, reduced burden or improved health - Other interventions such as supportive family education move beyond information giving by providing social support and developing coping strategies. As might be expected these interventions are able to reduce carer burden and distress and increase self efficacy. - More intensive programmes such as behavioural family therapy aim to address stressful relationships which commonly occur in families where a member is diagnosed with schizophrenia. These programmes are particularly effective in reducing carer burden and in some instances improving the carer’s coping and mental health. - Community based support such as intensive outreach when combined with low case loads is also effective in reducing carer burden and increasing coping. - There is some evidence that mutual support groups reduce carer burden and improve coping. … mutual support groups remained relatively ineffective in reducing carer burden - Finally there is minimal evidence that day care for those diagnosed with schizophrenia reduces carer burden or improves health and coping |
|  | Qualitative results  none |
|  | Limitations  High PICO heterogeneity, esp. for interventions; no structured quality rating, small populations |
|  | Authors conclusion  "A combination of education, mutual support and coping strategies delivered within an intensive community programme could effectively reduce carer burden and improve health and coping. Nurses could also deliver support to carers within structured intensive programmes" |
| qualitative richness | No/low (review contains only little qualitative content, only between the lines/implicitly) |

###### McGovern et al. 2021

| McGovern et al. 2021 | |
| --- | --- |
| study | McGovern, R., Smart, D., Alderson, H., Araújo-Soares, V., Brown, J., Buykx, P., Evans, V., Fleming, K., Hickman, M., Macleod, J., Meier, P. & Kaner, E. (2021). Psychosocial Interventions to Improve Psychological, Social and Physical Wellbeing in Family Members Affected by an Adult Relative’s Substance Use: A Systematic Search and Review of the Evidence. International Journal Of Environmental Research And Public Health, 18(4), 1793. https://doi.org/10.3390/ijerph18041793 |
| design | systematic review, controlled studies, narrative synthesis  authors background: Population Health Sciences, Behavioural, Management and Social Science, Humanities and Social Science …  investigators participative? no |
| objective | to systematically search and review the international published evidence for psychosocial interventions for family members affected by an adult relative’s substance use to determine the type of interventions used and what is known about the impact of the interventions at improving their psychological, social, and physical wellbeing |
| search period | 202211 |
| participants | psychiatric disorder: Substance Use  caregiver type: family members |
| interventions | - Psychosocial Interventions: any non-pharmacological intervention incl. but not limited to systemic family and couples therapy (approaches involving the family/partner to address problems in their relationships and interactions; helping family members to better understand one another, change negative behaviours, and resolve conflicts); unilateral family interventions (teaching strategies and skills to influence the substance-using relative’s behaviour and motivate change, with the primary aim of increasing treatment seeking behaviour in the substance patient); and psychological interventions which recognise that the substance use of a relative can negatively impact family members - Interventions that seeks to enhance their ability to cope, alleviate stress, or address trauma (e.g., forgiveness therapy, stress-strain-coping-support model, five step approach) |
| controls | Not specified |
| outcomes/ purpose | patient outcomes: none  family outcomes: psychological, social, or physical wellbeing (stress, coping, anxiety, or more generic psychological and mental health problems)   - social wellbeing: measures relating to family and relationships including reduced conflict and violence, improved family communication, and measures of child welfare (e.g., abuse/neglect and change in legal status) - Physical wellbeing: reduced physical health symptoms, disease, and health compromising behaviours (e.g., smoking, drug use, diet, and exercise) |
| body of evidence | 65 papers/58 trials; n=5955  publication years: 1984 to 2019  countries: USA; 5 Iran; 4 Australia; 3 UK, 3 Sweden; 2 Germany, 2 Spain; each 1 Brazil, Korea, Vietnam, the Netherlands, Mexico  quality/risk of bias: all studies high risk of selection bias (no blinding); majority high risk of performance bias (self-report outcomes), sometimes no random assignment |
| AMSTAR2 rating | - 1/5 critical items not met - no list of excluded studies |
| results | Descriptive results   - scientists background: not reported - participants details   - disorder type: substance abuse   - disorder onset/first treatment contact: not reported   - caregiver type: partner/spouse (n = 30), parents and/or children (n = 14) which included child aged <18 years (n = 3), adult child (n = 5), family (usually a combination of multiple relationship types, n = 14)   - recruitment: not reported   - patient age: not summarized   - percentage women (range): "Most of the trials reported on mainly female samples" - interventions details   - duration and frequency: not summarized; partly not reported in primary studies   - setting: 25 trials: to a single family member; 2 trials: primarily to a single family member, but option to attend sessions conjointly with the substance patient; 31 trials: conjoint intervention with the substance patient against   - components:   - Parental and Child Interventions (15 Trials) (incl. 5 for adult children)   - Behavioural Couples and Family Therapy (19 Trials)   - Systemic Family Interventions (6 Trials)   - Unilateral Family Interventions (7 Trials)   - Psychosocial Interventions for the Individual Affected Family Member (11 Trials)   - including components: behavioral couples/family therapy, CBT, Coping, Forgiveness therapy, skills training, case management, Emotional intelligence training, psychoeducation, stress management, CRAFT …   - personnel: not reported   - follow-up (range): not summarized |
|  | Quantitative results  Patient outcomes: none  Family outcomes:   - social outcomes for the affected family member (n = 34 trials) - psychological outcomes (n = 27 trials) - health outcomes (n = 8 trials)   No synopsis   - Behavioural interventions delivered conjointly with the substance patient and the affected family members 🡪 effective in improving the social wellbeing of family members (reducing intimate partner violence, enhancing relationship satisfaction and stability and family functioning). - adjacent individually focused therapeutic intervention component 🡪 affected adult family members may derive psychological benefit - attrition/adherence/drop-out/acceptability: not reported |
|  | Qualitative results  None. |
|  | Limitations  a high risk of allocation and performance bias and small sample sizes, not sufficiently powered to conduct reliable statistical testing or cost effectiveness analysis |
|  | Authors conclusion  "There is a large volume of literature examining psychosocial interventions … However, these interventions do not go far enough to address the needs often experienced within substance-affected families." |
| qualitative richness | None (review contains no qualitative statements) |

###### Meis et al. 2012

| Meis et al. 2012 | |
| --- | --- |
| study | Meis LA et al. Family Involved Psychosocial Treatments for Adult Mental Health Conditions: A Review of the Evidence. VA-ESP Project #09-009; 2012  also taken into account: Meis, L. A., Griffin, J. M., Greer, N., Jensen, A. C., Macdonald, R., Carlyle, M., Rutks, I. & Wilt, T. J. (2013). Couple and family involvement in adult mental health treatment: A systematic review. Clinical Psychology Review, 33(2), 275–286. https://doi.org/10.1016/j.cpr.2012.12.003 |
| design | systematic review of RCTs, meta-analyses  authors background: Chronic Disease Outcomes Research, Medical School, Department of Medicine  investigators participative? no |
| objective | (1) do family involved interventions improve patient symptoms, family functioning, or treatment engagement for adults with mental health conditions (i.e., efficacy; in comparison to no alternative psychosocial or psychological intervention) and  (2) which family involved interventions are superior to alternative individually-focused or family involved interventions (i.e., specificity or effectiveness compared to similarly, more, or less intensive interventions) at improving patient symptoms, family functioning, or treatment engagement? |
| search period | 19960101-20111231 |
| participants | psychiatric disorder: mental health conditions  caregiver type: not specified (exclusion of trials on caregiver distress/burden) |
| interventions | - family interventions - US trial only |
| controls | diverse (waitlist, medication management, attentional control, alternative individually-oriented, and/or alternative family interventions [less intensive, similar, or more intensive]) |
| outcomes/ purpose | patient outcomes: mental health symptoms (i.e., symptom severity, relapse rates, and days abstinent from drugs and/or alcohol), mental health treatment engagement  family outcomes: patient perception of couple/family functioning (i.e., couple/family adjustment, conflict, communication, and intimate partner violence)  "Interventions that solely seek to improve the distress and burden experienced by family members of those with mental illness are highly important, but beyond the scope of the present review." |
| body of evidence | 39 RCTs (51 references)  countries: only US studies  quality rating of the included studies: fair (n=25), poor (n=10), good quality (4) |
| AMSTAR2 rating | - 3/7 critical items not met - No protocol, no list of excluded studies, publication bias not assessed (at least not documented) |
| results | Descriptive results   - scientists background: not reported - participants details   - disorder type: substance use disorders (22 RCTs), schizophrenia spectrum disorders (4 RCTs), bipolar disorder (6 RCTs), PTSD (2 RCTs), sexual functioning disorders (2 RCTs); mixed depression, binge eating disorder, and nicotine dependence (1 RCT)   - disorder onset/first treatment contact: not summarized - caregiver type: not summarized - recruitment: not reported - mean age (range): not reported - percentage women (range): not reported - interventions details: 21 different active family treatments   - behavioral couples, marital, or family therapy for SUDs (BCT/BFT; 16 of 39 trials): dyadic (one patient and one family member/intimate partner) couple/family treatment, designed to address SUDs through cognitive–behavioral strategies to promote abstinence, involving the family member, and traditional behavioral couple therapy techniques to enhance communication, problem solving skills, and relationship satisfaction.   - community reinforcement and family training (CRAFT; 3 trials): family member-only intervention (patients not included) that supports and trains families in order to facilitate treatment entry of patients with SUDs who are not in treatment   - family-focused treatment (FFT, 4 trials): nine month psychoeducational intervention providing education on bipolar disorder, communication training, and problem-solving skills training to patients and their family members; FFT-variant = family-focused treatment-health promoting intervention; FFT-HPI) designed to address both patient and caregiver health behaviors   - all other interventions examined in 1 trial each |
|  | Quantitative results  Patient outcomes   - No high strength of evidence for efficacy found (table 3) - moderate strength of evidence for efficacy   - behavioural couple therapy vs. individual behavioural therapy for substance use disorder (outcomes: substance use, relationship adjustment)   - CRAFT vs. alternative family treatments for substance use disorders (outcome: treatment initiation) - All other interventions on all disorders: low strength of evidence, efficacy uncertain/not efficacious - Meta-analyses: - BCT/ ICBT; individual vs. couple/martial approaches - Outcome: days abstinent for alcohol and drug use - Post treatment MD 4.43 [2.16, 6.70]; 9 studies, n=577; I2=0% - After 6 months: MD 11.21 [7.17, 15.24; 9 studies, n=468; I2=0% - After 12 months: MD 11.93 [7.82, 16.04]; 8 studies, n=668; I2=0% - comparative effectiveness of family therapy to alternative approaches - "findings were mixed and broadly either favored family treatment or resulted in non-significant differences"   caregiver outcomes   - not focus of the review - reported for studies (e.g. burden, distress, anxiety, family/couple functioning, intimate partner violence, communication/conflict), not summarized   attrition/adherence/drop-out/acceptability: not reported |
|  | Qualitative results  None |
|  | Limitations   - "As family structure and family roles vary greatly across cultures (e.g., eastern versus western societies) and the role of family in health care systems is culturally bound (e.g., US health care versus socialized medicine in Europe or Canada), we limited our review to US studies." - Poor study quality; trials conducted on mostly white and male samples who were under 40 years old, lots of exclusion criteria, heterogeneity - „whereas posttreatment symptom severity was frequently reported, many RCTs did not assess family/couple functioning" |
|  | Authors conclusion  „Findings suggest behavioral couple or family therapy (BCT/BFT) reduced substance use (small-to-moderate effects) and improved relationship adjustment (large effects) compared to individually-oriented treatments. Community reinforcement and training (CRAFT) increased treatment initiation three-fold but did not improve substance use or family functioning over alternative family interventions. Family focused therapy for bipolar disorder improved symptoms over less intensive treatments with mixed findings when compared to equally intensive treatments. For both bipolar disorder and schizophrenia spectrum disorders, the few trials meeting our search criteria and heterogeneity among trials precluded generating broader conclusions regarding which family interventions are most effective for US populations" |
| qualitative richness | None (review contains no qualitative statements) |

###### Meyer et al. 2017

| Meyer et al. 2017 | |
| --- | --- |
| study | Meyer, T. D., Casarez, R., Mohite, S. S., La Rosa, N. & Iyengar, M. S. (2017). Novel technology as platform for interventions for caregivers and individuals with severe mental health illnesses: A systematic review. Journal Of Affective Disorders, 226, 169–177. https://doi.org/10.1016/j.jad.2017.09.012 |
| design | systematic review, all study designs, narrative synthesis  authors background: Psychiatry and Behavioral Sciences, Nursing, Public Health, Biomedical Informatics Core, Clinical Science & Translational Research  investigators participative? no |
| objective | to review and summarize recent advancements in such interventions for caregivers |
| search period | 201707 |
| participants | psychiatric disorder: Severe mental illnesses (recurrent or persistent mood disorders of at least moderate severity, non-affective psychotic disorders or borderline personality disorder  caregiver type: “informal caregivers” but none of the studies had data for caregivers, all patient-centric |
| interventions | interactive mobile or web-based software (using no or only minimal support from a professional) with and without patients |
| controls | Not restricted |
| outcomes/ purpose | patient outcomes: not predefined  caregiver outcomes: not predefined |
| body of evidence | N=0 studies on interventions for caregivers |
| AMSTAR2 rating | - 1/3 critical items not met (other items not applicable due to zero primary studies) - No list of excluded studies |
| results | Descriptive results  Not applicable. |
|  | Quantitative results  None. |
|  | Qualitative results  None. |
|  | Limitations  Differentiation between online and offline usage application was not always futile when searching for relevant literature. Some articles might have been missed due to this search strategy. |
|  | Authors conclusion  „none of those studies focused on caregivers, and the ones we identified using mobile or web-based applications were just for patients and not their relatives" |
| qualitative richness | None (review contains no qualitative statements) |

###### Morillo et al. 2022

| Morillo et al. 2022 | |
| --- | --- |
| study | Morillo, H., Lowry, S. & Henderson, C. (2022). Exploring the effectiveness of family-based interventions for psychosis in low- and middle-income countries: a systematic review. Social Psychiatry And Psychiatric Epidemiology, 57(9), 1749–1769. https://doi.org/10.1007/s00127-022-02309-8 |
| design | systematic review, all study designs, narrative synthesis  authors background: King’s College London; London School of Hygiene and Tropical Medicine  investigators participative? no |
| objective | To describe the key features and implementation strategies of family-based interventions and appraise their effectiveness |
| search period | 202108 |
| participants | psychiatric disorder: psychotic disorder including schizophrenia and bipolar disorder  caregiver type: one or more family member(s)  inclusion criteria: in low and middle-income countries (109 countries)  exclusion criteria: psychosis due to substance abuse |
| interventions | any form of family-based intervention (family intervention, family therapy, family psychoeducation, family workshops, “crisis intervention support for the family” , “family focused intervention” |
| controls | usual or absence of treatment |
| outcomes/ purpose | patient outcomes: patient condition (symptoms), self-management, social outcomes, delivery outcomes  caregiver outcomes: self-management, social outcomes, delivery outcomes |
| body of evidence | 27 studies (12 RCTs, 2 qualitative Studies, 2 descriptive studies, 4 non-controlled studies, 3 cluster RCTs, 2 controlled studies, 2 cost effectiveness analyses  publication years: 1993 to 2021  sample size: 1 (sic!) to 3092  countries: 11 China, 4 India, 2 Iran, 2 Vietnam, 2 Egypt, 1 South Africa, 1 Brazil, 1 Indonesia, 1 Thailand, 1 Nicaragua, 1 Pakistan  quality rating of the included studies: Integrated Quality Criteria for Review of Multiple Study Designs (ICROMS), CHEERS checklist: 6x "low“risk of bias, 2 "moderate", others "high" |
| AMSTAR2 rating | - 1/5 critical items no met - no list of excluded studies |
| results | Descriptive results   - study scientists background: in SR not reported - participants details   - disorder type: 20 schizophrenia, 3 schizophrenia+bipolar disorder, mixed   - disorder onset/first treatment contact: not reported   - caregiver type: not reported   - recruitment: not reported   - mean age (range): not reported   - percentage women (range): not reported - interventions details   - duration and frequency: 3 to 24 month (f.e. 12 weekly sessions, monthly home visits   - setting: inpatient, community sites (for home visits), outpatient, combination of in- and out-patient facilities, conducted within non-government organizations, orphanage   - delivery format: family members or patients+family members   - type: psychoeducation (f. e. Behavioral Family Psychoeducational Program (BFPEP)), family system approach, Cognitive-behavioural skills training for stress management, communication training, coping and problem-solving training, counselling; individual and family psychotherapy; Assertive Community Treatment (ACT)   - components: group therapy (heterogenous settings), home visits, calls, on-call availability, seminars and workshops; networking (professional, peers)   - personnel: mental health professionals: "village doctors", psychiatrists, clinical psychologists or therapists, psychiatric nurses (68%); social workers, researchers, non-government organization staff (32%)   - follow-up (range): 3 to 24 month - conceptual or theoretical underpinnings/psychotherapeutic components (24 studies): psychoeducation (22), therapeutic technique (12; individual therapy, family therapy), family systems approach   - Psychoeducation sessions: workshops, interactive discussions; 3 to 14 regular lectures lasting 15 min to 2 h; content: lectures about schizophrenia, treatments and rehabilitation, caring for a family member with psychosis, coping strategies, and how to care for them   - Family systems approach: highlights the dynamics within the family, addressing the interaction among members to affect the outcomes of the person with psychosis: expressed emotion, communication/interaction patterns.   - task-sharing: assigning care and support by family members, to provide quality aftercare and community networking, such as care networks and social support networks; home visits, home-based therapeutic support |
|  | Quantitative results  Narrative synthesis of patient outcomes:   - "Among the 27 studies, patient condition was mentioned 46 times from 26 studies, all reporting positive health impacts.“[recovery rate, symptom severity (negative symptoms), relapse rate] - mixed patients/caregiver outcomes (unclear, whether some of the outcomes were also measured for the family members): - "self-management outcomes appeared 17 times from 11 studies, all reporting positive health impacts.“[knowledge about schizophrenia, self-care skills] - "social outcomes were mentioned 12 times from 15 studies, with 3 studies reporting no change in social outcomes“[expressed emotion, social and occupational functioning, psychosocial functioning] - "3 studies presented positive delivery outcomes“[increase in attendance rates, service patient satisfaction] |
|  | Qualitative results  None. |
|  | Limitations   - high PICO heterogeneity - mostly high risk of bias - absence of treatment as control seems inadequate - no mention of effect sizes |
|  | Authors conclusion  "these findings may inform policymakers, healthcare providers, and academics to improve patient outcomes through a cost-effective intervention that can promote more effective task-sharing of quality care with the family" |
| qualitative richness | low (review contains only little qualitative content, see chapters "delivery strategies“and "discussion") |

###### Morton et al. 2021

| Morton et al. 2021 | |
| --- | --- |
| study | Morton, M., Wang, S., Tse, K., Chung, C., Bergmans, Y., Ceniti, A., Flam, S., Johannes, R., Schade, K., Terah, F. & Rizvi, S. (2021). Gatekeeper training for friends and family of individuals at risk of suicide: A systematic review. Journal Of Community Psychology, 49(6), 1838–1871. https://doi.org/10.1002/jcop.22624 |
| design | systematic review, all study designs, narrative synthesis  authors background: Psychiatry, Medical Science, Social Work, Suicide and Depression Studies Program  investigators participative? no |
| objective | To assess the characteristics of the gatekeeper training programs and their effectiveness in equipping family and friends with the knowledge and skills to care for and prevent a possible suicide of an individual under their care |
| search period | 20190719 |
| participants | psychiatric disorder: previous experience with suicide or are at risk of suicide  caregiver type: family and friends |
| interventions | Gatekeeper training program aimed at suicide prevention or caring for individuals at risk of suicide (providing support, providing resources, crisis management, etc.) |
| controls | Not predefined |
| outcomes/ purpose | patient outcomes: none  caregiver outcomes: knowledge, self-efficacy/confidence, attitudes (thoughts, beliefs, or feelings), Behavioral impacts/acquired skills |
| body of evidence | 17 studies (6 RCTs)  publication years: 2003 to 2020  countries: USA (11), Australia (3), Taiwan (2), Canada (1)  quality rating of the included studies: not assessed (only generally mentioned in discussion) |
| AMSTAR2 rating | - 3/5 critical items not met - No protocol, no list of excluded studies, risk of bias not assessed (only generally mentioned in discussion) |
| results | Descriptive results   - scientists background: not reported - participants details: not reported - interventions details   - duration and frequency: one-time workshop to multiple sessions over a 3‐month period   - format: workshop, conference, lecture   - components: needs assessment, Accpetance and Commitment Therapy, education, role‐playing note: „There were no time periods during any of the interventions for participants to discuss personal experiences, takeaways, or interpretations, and none facilitated the participant‐to‐participant provision of support."   - personnel: 15x healthcare professionals and/or suicide interventionists/ crisis; 1x peer-supported   - follow-up (range): not reported |
|  | Quantitative results  Reported separately for each study  Patient outcomes:   - "In the 17 studies included in this review, the behavioral outcomes focused solely on the trainees in the programs, neglecting any consideration of the impact these programs could have on the individuals at risk of suicide."   Caregiver outcomes:   - „significant post‐program impacts were found in knowledge, self‐efficacy, and gatekeeper‐related skills" - "findings in the attitude domain were inconclusive" - "gap in behavioral change observed across studies … , results appeared to be strongest when there was a significant program component that involved role play" - adherence/dropout: not reported - measures:   - Knowledge (SIRI-II, KAS, LAKS)   - Attitude (SOSS-SF, AAQ-II, SIQ, SAS, KAS, LAKS)   - Confidence (ORS-GS, RSE, SAS)   - Behavior (FAD, SCAS)   - Others (DDS, FFMQ-SF, ELS, ASQ-R, SDQ, HoN-OSCA, CSS)   Specific programs:   - - Question, Persuade, and Refer (QPR; QPR Institue, 2006; Quinnett, 1995)   - Applied Suicide Intervention Skills Training (ASIST; LivingWorks, 2010)   - Hope often looks like you (HOLLY)   - Jason Foundation's “A Promise for Tomorrow”   - Garrett Lee Smith Memorial Suicide Prevention Program   attrition/adherence/drop-out/acceptability: not reported |
|  | Qualitative results  None. |
|  | Limitations  diversity of study designs and outcome measures, some studies did not report the details of assessment questions; the specific program components that respond to the needs of family and friends remain poorly evaluated |
|  | Authors conclusion  „Gatekepper training (GKT) programs designed for FFSI are scarce, and few have been developed to specifically target family and friends alone. Despite this, GKT programs have a positive impact on improving subjective knowledge …, while also providing increased subjective self‐ efficacy and confidence in helping an individual at risk of suicide. " |
| qualitative richness | None (review contains no qualitative statements) |

###### NICE 2018

| NICE 2018 | |
| --- | --- |
| study | Post-traumatic stress disorder [G] Evidence reviews for psychological and psychosocial interventions for family members of people at risk of, or with, PTSD. NICE guideline NG116. 2018 |
| design | systematic review, RCTs, meta-analyses  authors background: National Guideline Alliance hosted by the Royal College of Obstetricians and Gynaecologists  investigators participative? no |
| objective | For family members (including children and carers) of people at risk of or with PTSD, do specific psychological, psychosocial or other nonpharmacological interventions result in an improvement in their mental health and wellbeing, a reduction in burden and improved social and occupational outcomes? |
| search period | 20180301 |
| participants | psychiatric disorder: (at risk of) posttraumatic stress disorder  caregiver type: family members |
| interventions | Psychological interventions, Psychosocial interventions, Other non-pharmacological interventions |
| controls | Not restricted |
| outcomes/ purpose | patient outcomes: Family member mental health, Family member wellbeing or quality  caregiver outcomes: carer mental health, carer wellbeing or quality of life, Carer burden; Employment, Housing, Lifestyle disruption, Relationship difficulties |
| body of evidence | 6 RCTs (2x at risk for PTSD, 4x PTSD)  publication years: 2004 to 2016  countries: USA (+Canada) (6)  quality rating of the included studies: risk of bias serious/very serious |
| AMSTAR2 rating | all critical items met |
| results | Descriptive results   - scientists background: not reported - participants details: not summarized, reported for each study separately   - disorder type: (at risk of) PTSD   - caregiver type: family, incl parents   - age: very heterogen incl. children - interventions details   - components (at risk for PTSD): only psychological interventions identified: problem solving, self-help without support   - components (PTSD): trauma-focused CBT, couple interventions, self-help without support |
|  | Quantitative results  At risk for PTSD:   - Problem solving vs. TAU (n=124): sig. benefit on caregiver mental health, no effect on quality of life [caregiver after traumatic brain injury] (low quality) - Self-help without support vs. attention placebo (n=66-105): small to moderate benefit in anxiety symptoms (up to 1 y) and depression symptoms (up to 6 months) [parents of children after paediatric ICU] (low quality)   With PTSD   - trauma-focused CBT vs. counselling (n=166,168); small to moderate effect on depression, no sig. effect on parenting difficulties [parents of children with PTSD] - couple interventions: - CB conjoint therapy (n=40) vs. waitlist: non sig. effect, suggesting higher discontinuation rate - CB conjoint therapy (n=41-57) vs. psychoeducation: non sig. effects - self-help without support vs. waitlist (n=41): sig. benefit on mental health, no sig. effects on relationship satisfaction   attrition/adherence/drop-out/acceptability: not information found |
|  | Qualitative results  None. |
|  | Limitations  Few studies, few outcomes available, poor study quality, inconsistent effects |
|  | Authors conclusion  not available.  Guideline recommendations, based on these evidence reviews and expert discussion:   - Involving and supporting families and carers - 1.4.5 Consider providing information and support to family members and carers of people with PTSD. This could cover:   - the treatment and management of trauma-related psychological and behavioural problems, including the person's possible risk to themselves and others   - discussing with family members and carers how they are being affected by the person's PTSD   - how they can support the person to access treatment, including what to do if they do not engage with, or drop out of treatment. [2018] - 1.4.6 Involve family members and carers, if appropriate, in treatment for people with PTSD as a way to:   - inform and improve the care of the person with PTSD and   - identify and meet their own needs as carers. [2018] - 1.4.7 Consider providing practical and emotional support and advice to family members and carers, for example directing them to health or social services or peer support groups. [2018] - 1.4.8 Think about the impact of the traumatic event on other family members because more than one family member might have PTSD. Consider further assessment, support and intervention for any family member suspected to have PTSD. [2018] - 1.4.9 For members of the same family who have PTSD after experiencing the same traumatic event, think about what aspects of treatment might be usefully provided together (such as psychoeducation), alongside individual treatments. [2018] |
| qualitative richness | None (review contains no qualitative statements) |

###### Norton et al. 2021

| Norton et al. 2021 | |
| --- | --- |
| study | Norton, M. J. & Cuskelly, K. (2021). Family Recovery Interventions with Families of Mental Health Service Patients: A Systematic Review of the Literature. International Journal Of Environmental Research And Public Health, 18(15), 7858. https://doi.org/10.3390/ijerph18157858 |
| design | systematic review, all study designs, narrative synthesis  authors background: Mental Health Engagement & Recovery, Principal Social Worker, Adult Mental Health Services, Adult Continuing Education  investigators participative? no |
| objective | to place focus on family recovery interventions in mental health |
| search period | 2010-2020 |
| participants | psychiatric disorder: not restricted (exclusion: addiction)  caregiver type: family |
| interventions | recovery interventions that focus on family in their own right: any individual or group-based interaction, that includes clinician to family member, peer-to-peer, online synchronise, asynchronis, self-help, recovery education, recovery colleges that focus specifically on family members/carers |
| controls | Treatment as usual |
| outcomes/ purpose | effectiveness (not specified)  identifying key enablers of family recovery interventions |
| body of evidence | 3 studies (exclusion of n=41 studies without qualitative content)  countries: all UK  quality rating of the included studies: 3x low quality |
| AMSTAR2 rating | - 1/5 critical items not met - No list of excluded studies |
| results | Descriptive results   - scientists background: not reported - participants details   - disorder type: 1x psychosis, 1x forensic mental health, 1x not specified   - disorder onset/first treatment contact: not reported   - caregiver type: relatives - Parents/Partners; Families - Parents/Siblings; Children of parents with mental health challenges   - recruitment: not reported   - mean age (range): not reported   - percentage women (range): not reported for all studies - interventions details   - duration and frequency: not reported   - setting: 2x Non-NHS Community Setting, 1x Low Security Forensic Mental Health Services   - delivery format: not reported   - components: 1x self-management intervention for relatives/ family recovery toolkit, 1x family psychoeducation programme, 1x Kidstime programme   - personnel: not reported   - follow-up (range): not reported |
|  | Quantitative results  Narratively reported: „several benefits to the aforementioned family recovery interventions. These included: education, social inclusion and facilitation of discussions on difficult topics" |
|  | Qualitative results  Themes and Subthemes   \| Themes \| Sub-Themes \| \| --- \| --- \| \| - Family Recovery Initiatives \| - Recovery Toolkit - Family Psychoeducation - Kidstime \| \| - Benefits \| - Education - Social Inclusion - Facilitation of Discussion of Difficult Topics \| \| - Challenges \| - Hidden Interventions (publicity) - Practicalities (access, availability 24/7, transport, group size) - Age Appropriate Interventions \| \| - Enablers for Recovery Interventions \| - Written Information - Access (no reliable information on family member) - Supports (for caregivers) - Decision Makers for Attending Interventions \| |
|  | Limitations  poor quality of studies, UK only |
|  | Authors conclusion  „The results of this review mirror much of the cultural and organisational attitudes towards families of those with mental health challenges " |
| qualitative richness | High (review focuses on qualitative content or review was designed for qualitative content) |

###### Okpoboro et al. 2014

| Okpokoro et al. 2014 | |
| --- | --- |
| study | Okpokoro U., Adams C. E. & Sampson S. Family intervention (brief) for schizophrenia. Cochrane Database Syst Rev. 2014 Mar 5;2014(3):CD009802. Doi:10.1002/14651858.CD009802.pub2. PMID: 24595545; PMCID: PMC7437394. |
| design | systematic review of RCTs, meta-analyses planned, but not possible  authors background: Cochrane Schizophrenia Group  investigators participative? no |
| objective | To assess the eJects of brief family interventions for people with schizophrenia or schizophrenia-like conditions. |
| search period | 201207 |
| participants | psychiatric disorder: schizophrenia or schizoaffective disorder  caregiver type: "family“ |
| interventions | 'family intervention' of brief duration (≤5 sessions or <3 months duration) |
| controls | Standard care, non-brief family interventions, any other non-family intervention |
| outcomes/ purpose | patient outcomes: hospital admission, relapse, global state, symptoms, social functioning, AE, cost  family outcomes: family burden, coping abilities, understanding of schizophrenia, care/maltreatment, expressed emotion, quality of life, satisfaction with care (either recipients of care or their carers) |
| body of evidence | 4 RCTs, n= 163 people with schizophrenia  publication years: 1987 to 2005  countries: US, UK, India  quality rating of the included studies: poor, often unclear, partly high; no blinding possible |
| AMSTAR2 rating | all critical items met |
| results | Descriptive results   - scientists background: not reported - participants details   - disorder type: schizophrenia/schizoaffective disorder   - disorder onset/first treatment contact: not reported; most with admissions in history   - caregiver type: differently defined: parents, then spouses, siblings, children and 'other'; "same household", "directly and actively involved in the care of the patient (supervising medication, bringing him/her to hospital for follow-up) for at least one month"   - recruitment: not reported   - age (range): 20 to 60   - 104 males, 59 females - interventions details   - duration and frequency: heterogen, mostly weekly sessions   - setting: heterogen (inpatient, outpatient, mixed)   - delivery format: sessions, workshops   - type: educational component; handling difficult problems' and handling communication and emotions   - personnel: not reported for all studies; investigator, nurse   - follow-up (range): 3 to 12 month |
|  | Quantitative results  patient outcomes:   - hospital admission (12 month): RR 0.50 (95% CI 0.22 to 1.11); n = 30, 1 RCT, very low quality evidence - relapse (4 month): RR 0.50 (95% CI 0.10 to 2.43); n = 40, 1 RCT, low quality evidence   family outcomes:   - understanding schizophrenia: MD 14.90, 95% CI 7.20 to 22.60, n = 70, 1 RCT, very low quality evidence - used family outcome instruments (measured in 2 studies):   - Burden Assessment Schedule - BAS (Thara 1998)   - Family Crisis Oriented Personal Evaluation Scales – FCOPES (McCubbin 1981)   - Family Emotional Involvement and Criticism Scale – FEICS (Shields 1992)   - Family Distress Scale - FDS (Pasamanick 1967)   - Patient Rejection Scale - PRS (Kreisman 1979)   attrition/adherence/drop-out/acceptability: only reported in 1 study |
|  | Qualitative results  none |
|  | Limitations  size and quality of studies; for many outcomes no meta-analysis possible |
|  | Authors conclusion  „the importance of brief family intervention should not be dismissed outright, with the present state of demand and resources available. The designs of such brief interventions could be modified to be more effective with larger studies, which may then have enough power to inform clinical practice." |
| Qualitative richness | None (review contains no qualitative statements) |

###### Petkari et al. 2020

| Petkari et al. 2020 | |
| --- | --- |
| study | Petkari, E., Kaselionyte, J., Altun, S. & Giacco, D. (2020). Involvement of informal carers in discharge planning and transition between hospital and community mental health care: A systematic review. Journal Of Psychiatric And Mental Health Nursing, 28(4), 521–530. https://doi.org/10.1111/jpm.12701 |
| design | systematic review, all study designs, narrative synthesis  authors background: Social and Community Psychiatry, Medical School  investigators participative? no |
| objective | To explore the evidence on the effectiveness of different models that involve carers in the transition between hospital and community mental health care |
| search period | 20200430 |
| participants | psychiatric disorder: severe mental disorders  caregiver type: Family members and friends (informal carers) |
| interventions | - discharge planning interventions - different models that involve carers in the transition between hospital and community mental health care - inclusion only if a-priori planning of carer involvement; individual only - exclusion: group interventions |
| controls | Not applicable |
| outcomes/ purpose | patient outcomes: positive clinical and psychosocial outcomes (eg. Relapse prevention or adherence)  caregiver outcomes: not predefined |
| body of evidence | 14 studies (of this, 8 RCTs, 2 NRCT)  publication years: 1978 to 2018  countries: USA (7), UK (3), Australia (1), Taiwan (1), Iran (2)  quality rating of the included studies: 8x moderate, 3x weak |
| AMSTAR2 rating | - 2/5 critical items not met - No list of excluded studies; risk of bias not mentioned in discussion |
| results | Descriptive results   - scientists background: not reported - participants details:   - disorder type: psychotic disorder (Schizophrenia, Schizoaffective, Bipolar) note: mood disorders included in search terms; but in results psychotic disorder only (unclear if excluded during screening or no search results)   - disorder onset/first treatment contact: not reported - caregiver type: family members and friends - recruitment: not reported - mean age (range): 15 to 65 - percentage women (range): 0% to 65% - interventions details:   - Intervention types by setting: - 1) programmes that offer education in hospital; - 2) programmes that involve carers in planning the patients discharge; - 3) programmes that involve carers in hospital care, discharge planning and also follow-up in the community   - Intervention type by intensity: - 1) purely psychoeducational programmes, which focused on providing psychoeducation to patients and their carers and prepare discharge planning (1 study) psychoeducation sessions as lecture and question-and-answer - 2) programmes that involved carers in planning the transition from the psychiatric inpatient treatment to community mental health services (4 studies) components: discharge planning meetings, information gathering and carer needs assessment, problem-solving, communication training, counselling referral services, stress management, home visits by advanced practice nurse (1 study), „collaborative roles between the patient and the family members for maintaining medication adherence" - 3) programmes that bridged into the aftercare involving carers in follow-up after the patient had been discharged from hospital (9 studies) components: involvement of carers in outpatient visits; behavioural family interventions, systemic therapy, social skills training; follow-up phone calls, follow-up home visits, Psychoeducation, information on community resources, problem-solving; family therapy/treatment, social skills training for patients |
|  | Quantitative results  No synopsis, reported separately for each study  Patient outcomes:   - not summarized; - assessed: re-admissions, relapse, treatment adherence, compliance, treatment satisfaction, medication side effects, functioning, social skills - “These studies found that the comprehensive programmes included had an effect in reducing relapse.”   caregiver outcomes (assessed in only few studies):   - Level of functioning, burden, health status, knowledge, availability of support, expressed emotion   attrition/adherence/drop-out/acceptability: not reported |
|  | Qualitative results  None. |
|  | Limitations  heterogeneity of the treatment models and of the outcomes assessed, different countries with different health care systems |
|  | Authors conclusion  „The most comprehensive interventions, i.e. those including psychoeducation, care planning and aftercare follow-up were better evaluated and showed a clearer benefit in improving long-term outcomes and, in particular, reduce re-hospitalization" |
| qualitative richness | None (review contains no qualitative statements). |

###### Pharoah et al. 2010

| Pharoah et al. 2010 | |
| --- | --- |
| study | Pharoah F, Mari JJ, Rathbone J & Wong W. Family intervention for schizophrenia. Cochrane Database of Systematic Reviews 2010, Issue 12. Art. No.: CD000088. DOI: 10.1002/14651858.CD000088.pub3. |
| design | systematic review of RCTs/quasi-RCTs, meta-analyses  authors background: Cochrane Schizophrenia Group  investigators participative? no |
| objective | To estimate the effects of family psychosocial interventions in community settings for people with schizophrenia or schizophrenia-like conditions compared with standard care |
| search period | 200809 |
| participants | psychiatric disorder: schizophrenia  caregiver type: family |
| interventions | community-orientated family-based psychosocial intervention |
| controls | Standard care |
| outcomes/ purpose | patient outcomes: Suicide and all causes of mortality, Service utilisation, Hospital admission, Clinical global response, Relapse  family outcomes: burden, coping, understanding, expressed emotion, quality of life/satisfaction with care for either recipients or carers |
| Body of Evidence | 53 RCTs  publication years: 1978 to 2007  countries: Australia (2), Canada (1), Europe (12), China (28), USA (10)  quality rating of the included studies: "poor" |
| AMSTAR2 rating | all critical items met |
| results | Descriptive results   - scientists background: not reported - participants details   - disorder type: schizophrenia   - disorder onset/first treatment contact   - caregiver type: not specified   - recruitment: not reported   - mean age (range): not sumerized   - percentage women (range): not sumerized - interventions details   - duration and frequency: 6 weeks to 3 years   - setting: presence of patient n=13; groups or relatives n=8   - delivery format: not sumerized (heterogen)   - type: educational component (all); partly 24h-support, motivational interviewing, CBT, relaxation training, role-play   - personnel: not reported   - follow-up (range): not sumerized |
|  | Quantitative results  patient outcomes:   - relapse (n = 2981, 32 RCTs, RR 0.55 CI 0.5 to 0.6, NNT 7 CI 6 to 8), - hospital admission (n = 481, 8 RCTs, RR 0.78 CI 0.6 to 1.0, NNT 8 CI 6 to 13) - compliance with medication (n = 695, 10 RCTs, RR 0.60 CI 0.5 to 0.7, NNT 6 CI 5 to 9) - … general social impairment and the levels of expressed emotion within the family - Suicides: neither prevention nor promotion   caregiver outcomes   - narrative reporting separately for each study because of heterogenous endpoint measures   used tools:   - Coping with Life-events& Difficulties Interview COPI - Family Support Service Index - FSSI - Family Assessment Device FAD - Family Burden Interview Schedule FBIS - Camberwell Family Interview – CFI - Clinical Interview Schedule Revised - CIS-R - Experience of Care giving Inventory – ECI - Family Questionnaire – FQ - Verona Service Satisfaction Scale – VSSS - Ways of Coping – WOC - Self Evaluation and Social Support Schedule – SESS - Adaptability, Partnership, Growth, Affection, and Resolve - APGAR   attrition/adherence/drop-out/acceptability:   - tendency of individuals/families to leave care (n = 733, 10 RCTs, RR 0.74 CI 0.5 to 1.0) |
|  | Qualitative results  none |
|  | Limitations  poor methodological quality, high PICO heterogenity |
|  | Authors conclusion  „people with schizophrenia and their families should be willing to spend a significant amount of time in contact with services." |
| Qualitative richness | None (review contains no qualitative statements) |

###### Piat et al. 2015

| Piat et al. 2015 | |
| --- | --- |
| study | Piat, M., Pearson, A., Sabetti, J., Steiger, H., Israel, M. & Lal, S. (2015). International training programs on eating disorders for professionals, caregivers, and the general public: A scoping review. Journal Of Eating Disorders, 3(1). https://doi.org/10.1186/s40337-015-0066-y |
| design | scoping review, all study designs, narrative synthesis  authors background: Mental Health  investigators participative? no |
| objective | To identify and synthesize published training programs on eating disorders (ED) (anorexia nervosa or bulimia nervosa) for professionals, natural supporters of people with ED, or the public |
| search period | Not reported (probably 2014) |
| participants | psychiatric disorder: eating disorders (note: not adults only)  caregiver type: family members or other caregivers of people with ED (note: not on interventions for caregivers only but also for professionals and "the public") |
| interventions | - training programs - exclusion: ED intervention studies |
| controls | Not restricted |
| outcomes/ purpose | patient outcomes: not predefined  caregiver outcomes: not predefined |
| body of evidence | 25 articles (20 evaluation studies on 14 ED training programs; of this 10 for "natural supporters"; of this 5 on adults patients); 3 RCTs  publication years: 1998 to 2014  countries: Canada (3), UK (5), USA (7), Australia (3), Norway (2)  quality rating of the included studies: not assessed (not required for scoping reviews) |
| AMSTAR2 rating | - 1/3 critical items not met - No protocol, no list of excluded studies - AMSTAR2 applicable to scoping reviews only to a limited extent |
| results | **Descriptive results** (for caregiver interventions only)   - scientists background: not reported - participants details   - disorder type: n=17 eating disorders; at risk for ED n=3   - disorder onset/first treatment contact: not reported   - caregiver type: "natural supporters (parents or other family members, carers, friends)"   - recruitment: not reported   - mean age (range): not reported; 8 studies on children/adolescents, 14 studies all ages   - percentage women (range): not reported - interventions details: not summarized; extracted from table   - duration and frequency: not reported   - setting: not reported   - delivery format: not always reported, incl. Web-based, mutual support   - components: theory, instruction, knowledge; skills coaching (based on behavioral therapy); narrative family therapy and psycho-educational approaches, emotion-focused therapy, mindfulness strategies, dialectical behavior therapy   - personnel: not reported   - follow-up (range): not reported - program titles (programs for caregivers of adults with ED only):   - no name   - Maudsley eating disorder collaborative care workshops   - Collaborative care skills training workshops (Maudsley)   - Overcoming Anorexia Online (OAO)   - The parent partner program™ |
|  | Quantitative results  No effect sizes reported  Patient outcomes: not reported  caregiver outcomes   - improved knowledge and related skills - reduced distress and burden - better coping and communication - improved family functioning   attrition/adherence/drop-out/acceptability: not reported |
|  | Qualitative results  key themes   - affirmation as good parents, skills acquired in training transferable to other areas of parenting - need for connectedness and support, particularly among parent trainees, - being able to break isolation and “externalize the illness” - need for ongoing exchange with others - extended support through “alumni groups” or “buddy“ systems |
|  | Limitations  heterogeneity of studies, divergent objectives of the training programs, wide array of methodologies; no quality rating of included studies, no effect sizes reported, no patient outcomes |
|  | Authors conclusion  „Results suggest that the supportive, face-to-face element of training for families and natural supporters, both between trainers and trainees and among trainees themselves, was highly beneficial. This implies that ED training using passive learning approaches may be less effective for families, for whom the lived experience of training was an added value." |
| qualitative richness | Moderate (review contains explicit qualitative statements, even if this was not the primary focus of the study); see chapter results/discussion) |

###### Rane et al. 2017

| Rane et al. 2017 | |
| --- | --- |
| study | Rane, A., Church, S., Bhatia, U., Orford, J., Velleman, R. & Nadkarni, A. (2017). Psychosocial interventions for addiction-affected families in Low and Middle Income Countries: A systematic review. Addictive Behaviors, 74, 1–8. https://doi.org/10.1016/j.addbeh.2017.05.015 |
| design | systematic review, all study designs, narrative synthesis  authors background: Psychiatry and Human Behaviour, Psychology, Addictions Research Group  investigators participative? no |
| objective | to: 1. Assess the size and scope of available research literature on psychosocial interventions to directly help AFMs in LMIC, 2. Describe these psychosocial interventions, and 3. Identify the research evidence for their effectiveness, acceptability, and feasibility |
| search period | 2003-2014 |
| participants | psychiatric disorder: substance use disorders  caregiver type: affected family members (AFM): immediate family, other relatives and friends |
| interventions | psychosocial interventions directed towards people affected by addiction |
| controls | Not predefined |
| outcomes/ Purpose | patient outcomes: Not predefined  caregiver outcomes: Not predefined |
| body of evidence | 4 studies (1 cluster-RCT), n= 137  publication years: 2003 to 2014  countries: Mexico (2), Vietnam, Malaysia  quality rating of the included studies: not assessed |
| AMSTAR2 rating | - 3/5 critical items not met - Not list of excluded studies, study quality not assessed, risk of bias not mentioned in discussion |
| results | Descriptive results   - scientists background: not reported - participants details   - disorder type: substance use disorder   - disorder onset/first treatment contact: not reported   - caregiver type: parents, siblings, spouses   - recruitment: not reported   - mean age (range): not reported   - percentage women (range): „predominantly female participants" - interventions details   - duration and frequency: 4-12 months; weekly or monthly sessions   - setting: not reported   - delivery format: groups (3), individual (1)   - components: family psycho-educational (FPE), Rational Emotive Behaviour Therapy (REBT), 5-Step Method   - components: psycho-education; support, coping skills, behaviour change; correct cognitive bias and defective information, emotional regulation strategies (deep diaphragmatic breathing, progressive muscle relaxation, modelling, role play; exploring experiences, information giving, exploring support available, referring to specialised sources of help   - personnel: counsellors, volunteers, former drug patients; health educators or local health workers; trained therapists   - follow-up (range): not reported for each study (probably no follow-up after end of intervention) |
|  | Quantitative results  Sometimes it is not clear, whether outcomes were assessed for patients or caregivers (or both).  Reported for each study separately, "a qualitative synthesis of the available findings suggests that there was lowering of psychological and physical distress, along with a better understanding of the patient’s addictive behaviour and better coping; with associated improvements in self-esteem and assertive behavior“  Patient outcomes:   - Symptom change (ZungSelf Rating Scale, Symptom Rating Test) - psychological symptoms (eg depression) - drug use behaviour (Addiction Severity Index)   caregiver outcomes   - coping behaviour (Brief COPE Scale, Coping Questionnaire) - assertiveness (Assertion Inventory) - self-esteem (Self-esteem Inventory)   attrition/adherence/drop-out/acceptability: not reported; 2 studies assessed acceptability (satisfaction) |
|  | Qualitative results  None. |
|  | Limitations  evidence base extremely sparse, studies either exploratory or pilot trials with small sample sizes, predominantly female participants; measurement of varied components |
|  | Authors conclusion  "The limited evidence does suggest positive benefits to AFMs. The scope of research needs to be extended to other addictions, and family members other than spouse and female relatives." |
| qualitative richness | None (review contains no qualitative statements) |

###### Reupert et al. 2013

| Reupert et al. 2013 | |
| --- | --- |
| study | Reupert, A. E., Cuff, R., Drost, L., Foster, K., Van Doesum, K. T. M. & Van Santvoort, F. (2012). Intervention programs for children whose parents have a mental illness: a review. MJA Open, 1(Suppl 1), 18–22. https://doi.org/10.5694/mjao11.11145 |
| design | systematic review, all study designs, narrative synthesis  authors background: Faculty of Education, Mental Healthy, Nursing Schoo  investigators participative? no |
| objective | To identify and describe intervention programs to improve outcomes for children whose parents have a mental illness |
| search period | 201106 |
| participants | psychiatric disorder: parental mental illness (excluding parental substance abuse)  caregiver type: children |
| interventions | programs specifically targeting children whose parent/s have a mental illness |
| controls | Not restricted |
| outcomes/ purpose | Program description, target group, evidence base |
| body of evidence | not summarized (n=27 incl. Grey literature?)  quality rating of the included studies: not assessed  countries: not reported |
| AMSTAR2 rating | - 2/3 critical items not met; AMSTAR2 limited applicable - No protocol, no list of excluded studies |
| results | Descriptive results   - scientists background: not reported - participants details: not reported - interventions details: not reported - clustering of programs: - Family-intervention programs for children whose parents have a mental illness (n=7) 6/7 on depression/bipolar and/or anxiety (USA 5, Germany 1 <https://www.effekt-training.de/effekt-e> , Finland 1) Age of children: range 4-7 to 15-16 Weekly or monthly session, some directed to parents, some to the children and some to the whole family; group meetings (other families) Outcomes: family dysfunction, children’s support networks and competencies, emotional disruption, hyperactivity, anxiety, depression, internalising symptoms, stress, prosocial behavior, emotional symptoms  Components   - psychoeducation, problem-solving skills, communication skills, coping, CB-sessions   - care-coordination model for individual families - Peer-support programs for children whose parents have a mental illness (n=12) disorder type not reported (5 Australia, 3 USA, 1 UK, 1 Germany <https://www.beratungsstelle-suedviertel.de/gruppenangebote/auryn-gruppe/> , 1 Canada, 1 Netherlands) school holiday programs, after-school programs, or camps  age 7– 18 years Weekly or monthly session, mostly in groups, partly separate parents sessions By peer leader or/and health professional Outcomes: self-esteem, coping skills, connections within family, depression, emotional symptoms, confidence, knowledge, mental health literacy, awareness, prosocial behavior, life satisfaction, problem-solving, stigma, substance use, emotional and behavioural problems, negative cognitions, social support, competence, parent–child interaction, life skills Components   - Psychoeducation, coping and interpersonal skills; social support   - role plays, games, homework assignments   - leisure activities, artwork, games, singing, interactive and relaxation exercises   - newsletters, email contact, drop-in group - Online interventions (n=2) monitored chat groups, group meeting, - Bibliotherapy (n=2) |
|  | Quantitative results  Narratively reported for each program separately, no synopsis |
|  | Qualitative results  None. |
|  | Limitations  Poor reporting, no synopsis on quantitative outcomes |
|  | Authors conclusion  „The core component across programs is the provision of psychosocial education to children about mental illness. More rigorous research is required to establish the conditions through which children’s outcomes are enhanced." |
| qualitative richness | Low (review contains only little qualitative content, see chapter results) |

###### Rodolico et al. 2022

| Rodolico et al. 2022 | |
| --- | --- |
| study | Rodolico, A., Bighelli, I., Avanzato, C., Concerto, C., Cutrufelli, P., Mineo, L., Schneider-Thoma, J., Siafis, S., Signorelli, M. S., Wu, H., Wang, D., Furukawa, T. A., Pitschel-Walz, G., Aguglia, E. & Leucht, S. (2022). Family interventions for relapse prevention in schizophrenia: a systematic review and network meta-analysis. The Lancet Psychiatry, 9(3), 211–221. https://doi.org/10.1016/s2215-0366(21)00437-5 |
| design | systematic review of RCT with meta analyses and network meta analysis  authors background: psychiatry and psychotherapy  investigators participative? no |
| objective | compare the efficacy, acceptability, and tolerability of family interventions for relapse prevention in schizophrenia |
| search period | 20210715 |
| participants | psychiatric disorder: schizophrenia spectrum disorders (at least 80%)  caregiver type: not predefined  inclusion criteria: at least 80% of patients have schizophrenia spectrum disorder, relapse rate reported, only if family intervention included  exclusion criteria: acutely ill patients, concurrent medical or psychiatric disorder, prodromal or "at risk of psychosis" |
| interventions | Family intervention |
| controls | Treatment as usual, brief family interventions |
| outcomes/ purpose | patient outcomes: relapse at 12 months overall, positive, negative, and depressive symptoms of schizophrenia, quality of life, adherence, overall functioning, expressed emotion, discontinuations due to inefficacy  caregiver outcomes: family burden, expressed emotion |
| body of evidence | 90 studies (RCTs), n= 10.340  sample size: mean 116 (range 19–1268)  publication years: 1981 to 2019  countries: China (30), USA (16), UK (12), Italy (6), Germany (5), Spain (4), Denmark (2), India (2), Japan (2), Turkey (2), Australia (2), Norway (1), Jordan (1), The Netherlands (1), Hong Kong (1), Indonesia (1), Poland (1), Colombia (1), Thailand (1), Malaysia  quality rating of the included studies: 49 moderate risk of bias, 33 high risk, 0 low risk |
| AMSTAR2 rating | all critical items met |
| results | Descriptive results   - scientists background: not reported participants details   - disorder type: schizophrenia   - mean duration of illness: 6,6y   - caregiver type: not described in detail   - recruitment: not reported   - mean age (range): 31,3 years [range 14-65]   - percentage women: 38,4% (range not reported) - interventions details: clustering of family intervention models: - Systemic-oriented family intervention (whole family is target); 6 RCTs, median 8 sessions - Psychoeducational approaches to family **with** the patient   - - 2 Family psychoeducation (patient+relatives simultaneously, only information, not behavioural); 15 RCTs, median 12 sessions     - 3 Family psychoeducation + family behavioural or skills training (broad); 14 RCTs, median 17 sessions     - 4 Family psychoeducation + family behavioural or skills training (crisis-oriented); 3 RCTs, median 6 sessions     - 5 Family psychoeducation + family behavioural or skills training with focus on family emotional climate; 19 RCTs, median 29 sessions     - 6 Family psychoeducation + family behavioural or skills training and mutual skills training; 8 RCTs, median 72 sessions - Psychoeducational interventions to the family **without** the patient   - - 7 Relatives' psychoeducation; 12 RCTs, median 18 sessions     - 8 Relatives' psychoeducation + patient psychoeducation; 8 RCTs, median 14 sessions - Integrated interventions (**separate** for patients and the whole family)   - - 9 Family psychoeducation + patient behavioural skills training; 15 RCTs, median 35 sessions     - 10 Community-based care interventions (mostly assertive; family intervention part of larger psychosocial intervention); 6 RCTs, median 143 sessions - Control conditions   - - brief family or relatives' psychoeduction; 6 RCTs, median 2 sessions     - treatment as usual; 80 RCTs |
|  | Quantitative results  12 months relapse rate (NMA with 82 studies, 91 pairwise comparisons): (for other time points see figure 3)   - Family psychoeducation 9,7%; OR 0,18 (0,12–0,27); 9 comparisons - Systemic family-oriented intervention 16,3%; OR 0,33 (0,17–0,66); 4 comp. - Family psychoeducation (all forms): 17,1-22,4%; OR 0,35 (0,23–0,53) - Relatives psychoeducation (all forms): 21,8%-23,2%; OR about 0,5 - Community-based care intervention: 27%; OR 0,63 (0,42–0,94); 6 comp. - Brief family psychoeducation: 32,8%; OR 0,83 (0,42–1,64); 2 comp. - Treatment as usual: 37% (OR 1 = reference)   caregiver outcomes: “data on … expressed emotion, and the family burden were too scarce to be informative”  attrition/adherence/drop-out/acceptability: not reported: “most … not differ or were lower than for treatment as usual” |
|  | Qualitative results  None. |
|  | Limitations  Partly indirect evidence, partly low statistical power; many studies with high bias risk; wide range of relapse definitions; variability in the level of detail in the intervention descriptions in the study manuscripts; exclusion of many studies that focused on caregiver outcomes (but not reported relapse rate) |
|  | Authors conclusion  "Almost all family intervention models were efficacious in preventing relapse in schizophrenia. Family psychoeducation alone, without behavioural or skills training, was superior to the more complex models. The simplest form of family psychoeduation was ranked among the most efficacious interventions" |
| qualitative richness | None (review contains no qualitative statements) |

###### Rushton et al. 2023

| Rushton et al. 2023 | |
| --- | --- |
| study | Rushton, C., Kelly, P.J., Raftery, D., Beck, A. & Larance, B. (2023). The effectiveness of psychosocial interventions for family members impacted by another’s substance use: A systematic review and meta-analysis. Drug and Alcohol Review, 42, pp. 960-977. |
| design | Systematic review. RCT, non-RCT, pre-post studies, observational analytic designs  Authors background: Psychology  investigators participative? No. |
| objective | To examine the effectiveness of psychosocial interventions for improving the wellbeing of family members impacted by another’s substance use. |
| search period | Not reported (before June 2022). |
| participants | psychiatric disorder: substance use disorder (SUD); adults only  caregiver type: concerned significant others, caregivers, partners, adult children and friends.  exclusion criteria: Interventions for family bereaved by substance use or treatment aimed primarily at patient |
| interventions | Psychosocial interventions directed at family members (group or individual). |
| controls | Passive and active controls |
| outcomes/ purpose | patient outcomes: none  caregiver outcomes: psychological functioning, quality of life, physical health and substance use, and general functioning |
| body of evidence | 19 studies: 11 RCTs, 3 non-RCTs with control group, 5 quasi-experimental pre-post design study (10 meta-analysis); N = 2679  publication years: 2011 - 2020  countries: USA, Germany, Spain, Canada, Iran, Sweden, Australia, Vietnam, India, UK  quality rating of the included studies: either Cochrane Risk of Bias Tool (for RCTs) (8 some concerns, 2 high). ROBINS-I (non-randomised control trials) (2 moderate, 7 serious). |
| AMSTAR2 rating | - 2/7 critical items not met - No list of excluded studies, Publication bias not assessed |
| results | Descriptive results   - participants details   - disorder type: SUD   - disorder onset/first treatment contact: not reported   - caregiver type: concerned significant others, caregivers, partners, adult children and friends   - recruitment: not reported   - mean age (range): 31,1 to 55,0   - percentage women (range): 40% to 100% - interventions details   - duration and frequency: two weekend workshops (27h), 4-12 sessions á 30-120 min.   - setting: not reported   - delivery format: group, individual, multifamily; internet based, face-to-face   - types: CRAFT, support/mutual-support programs   - components: group support, coping skills, psychoeducation, delivering information, communication skills, social support), group cognitive-behavioural therapy, positive psychology and emotion regulation training   - personnel: clinicians, social workers, counsellors, self-paced   - follow-up (range): 24 weeks to 12 months |
|  | Quantitative results  Patient outcomes:   - not assessed   Caregiver outcomes:   - individually administered interventions - depression: SMD 0,50, (95% CI 0.21 to 0.79); 3 RCTs - distress (SMD 0.28, 95% CI 0.03, 0.54); 3 RCTs - family functioning (d = 0.51, 95% CI 0.28, 0.73) 2 pre-post studies - coping (d = 0.43, 95% CI 0.24, 0.61) 2 pre-post studies - group interventions - depression (d = 0.50, 95% CI 0.17, 0.82); 3 non-RCTs + 2 pre-post designs - distress (d = 0.44, 95% CI 0.13, 0.75); 3 non-RCTs + 2 pre-post designs - coping (d = 0.81, 95% CI 0.29, 1.33); 3 non-RCTs + 2 pre-post designs   attrition/adherence/drop-out/acceptability: not reported |
|  | Limitations  All studies rated as having either some concerns or high/serious risk of bias, insufficient description of methodology, small sample sizes, high drop-outs and limited long-term follow-ups. |
|  | Authors conclusion  “The expansion of services and interventions for families impacted by another’s SUD is a priority that is frequently overlooked in mainstream alcohol and other drug service provision. This review provides a comprehensive overview and synthesis of contemporary evaluations that may help to inform future service provision. Findings demonstrate favourable outcomes for families regardless of whether interventions are delivered to individuals or groups, particularly with respect to reducing families’ depression and distress. Although caution is warranted, these findings are promising given the considerable burden experienced by families and the historical omission of families within the evaluation literature.” |
| qualitative richness | None (review contains no qualitative statements). |

###### Sin et al. 2017

| Sin et al. 2017 | |
| --- | --- |
| study | Sin, J., Gillard, S., Spain, D., Cornelius, V., Chen, T. & Henderson, C. (2017). Effectiveness of psychoeducational interventions for family carers of people with psychosis: A systematic review and meta-analysis. Clinical Psychology Review, 56, 13–24. https://doi.org/10.1016/j.cpr.2017.05.002 |
| design | systematic review of RCTs with meta-analyses  authors background: Population Health/Health Service/Public Health Research; Psychiatry, Clinical Sciences  investigators participative? no |
| objective | 1. to assess the effectiveness of psychoeducation on family carers' wellbeing, health morbidities, and caregiving-related outcomes; 2. to identify intervention-factors (such as intervention duration, contact time, and different modes of delivery), which may moderate intervention effectiveness. |
| search period | 20160531 |
| participants | psychiatric disorder: psychosis „however defined and treated in any setting"  caregiver type: parents, siblings, spouses, close friends |
| interventions | - psychoeducational interventions professionally-led (although those which involved co-facilitation from a family carer or other lay-person were not excluded) - interventions with or without the patients - exclusion: Pure bibliotherapy, and treatment programmes that solely relied on educational materials (such as booklets or non-interactive websites), but which comprised no actual interaction; interventions <4 weeks |
| controls | inactive (waitlist, TAU …) or active (others than psychoeducation: cognitive behavioural therapy, counselling, or family intervention |
| outcomes/ purpose | patient outcomes: not assessed  caregiver outcomes: wellbeing, quality of life, global morbidities, burden, expressed emotion |
| body of evidence | 32 RCTs, n= 2858 carers, n=1305 patients (out of 15 RCTs that included patients too)  publication years: 1987 to 2016  countries: China (10), North America (4), Europe (4), UK (4) Middle East (6), South America (1), Australia (2), India (1)  quality rating of the included studies: mostly low or unclear, but no masking possible |
| AMSTAR2 rating | - 1/7 critical items not met - no list of excluded studies |
| results | Descriptive results   - scientists background: not reported - participants details   - disorder type: psychotic disorder, mostly schizophrenia   - disorder onset/first treatment contact   - caregiver type: mostly parents, especially mothers   - recruitment: 4 RCT while patients in hospital, all others while patients living in the community   - mean age (range): not reported   - percentage women (range): not reported in detail - interventions details   - duration and frequency: 4-52 weeks (mean 20 weeks); contact times 6 to 42 h (mean 17.4 h)   - setting: not reported in detail   - delivery format: mostly face-to-face; 3 individual, 19 groups, 4 combined both; partly incl. telephone and/or email-support , 1 web-based psychoeducation programme   - components: psychoeducation   - personnel: not reported in detail, "professionally-led"   - follow-up (range): 1 to 24 months |
|  | Quantitative results  patient outcomes: not assessed  caregiver outcomes:   - carers' wellbeing (SMD 0.103, 95% CI −0.186 to 0.392, I2=0%), 2 RCTs, n=184 carers - carers' stress (SMD −0.169, 95% CI −0.410 to 0.072, I2= 0%), 4 RCTs, n = 266 carers - carers' global morbidities (SMD −0.230, 95% CI −0.386 to −0.075, I2= 25.6%), 7 RCTs, n = 656 - depression (SMD −0.70, 95% CI −0.97 to −0.44, I2= 97.2%), 2 RCTs, n =245 - perceived burden (SMD −0.434, 95% CI −0.567 to −0.31, I2=0%), 10 RCTs, n = 878 - perceived burden 6 month post-intervention(SMD −1.628, 95% CI−2.307 to−0.948, I2= 94.5%), 10 RCTs, n = 821 - negative caregiving experiences (SMD −0.210, 95% CI −0.396 to −0.025, I2=0%), 5 RCTs, n =446 - expressed emotion (SMD −0.161, 95% CI −0.367 to −0.045, I2=0%), 5 RCTs, n= 337   attrition/adherence/drop-out/acceptability: not reported  Meta-regression:   - contact time, intervention duration, mode of delivery no moderator of effects |
|  | Qualitative results  None. |
|  | Limitations   - Carers' outcomes were measured in a variety of ways using different scales and follow up data beyond the end of the intervention were sparse - Follow up data beyond the post-intervention period were sparse - high risk of inadequate power |
|  | Authors conclusion  "how these carers' outcomes correlate to patients' outcomes like decreased relapse and better compliance, and to family-wide outcomes like family relationship and communication, and vice versa, needs to be better explored." |
| qualitative richness | Low (review contains only little qualitative content, see chapter results [esp. meta-regression], discussion, Limitations) |

###### Spain et al. 2017

| Spain et al. 2017 | |
| --- | --- |
| study | Spain, D., Sin, J., Paliokosta, E., Furuta, M., Prunty, J. E., Chalder, T., Murphy, D. G. & Happé, F. G. (2017). Family therapy for autism spectrum disorders. Cochrane Library, 2017(5). https://doi.org/10.1002/14651858.cd011894.pub2 |
| design | systematic review of RCTs  authors background: Cochrane; Psychiatry, Psychology & Neuroscience; Child and Adolescent Mental Health Service and Social Communication Assessment Service; Human Health Sciences; Translational Neurodevelopment  investigators participative? no |
| objective | To evaluate the clinical effectiveness and acceptability of family therapy as a treatment to enhance communication or coping for individuals with ASD and their family members. If possible, we will also seek to establish the economic costs associated with family therapy for this clinical population |
| search period | 20170116 |
| participants | psychiatric disorder: autism spectrum disorders (all ages)  caregiver type: family (parents, grandparents, siblings, children, or spouses), either biologically related to the individual with ASD, or related through marriage or cohabitation; non-professional carers (e.g. individuals providing foster or respite care) and significant others such as friends. |
| interventions | - family therapy interventions delivered by at least one suitably qualified clinician, derived from systemic theories systemic therapy; structural family therapy; strategic family therapy; Milan approaches; solution-focused therapy; narrative therapy; and behavioural family therapy face-to-face or web-based - exclusion: pure bibliotherapy, psychoeducation or parent training techniques |
| controls | No treatment, usual care, waitlist, active comparator (alternative psychological intervention) |
| outcomes/ purpose | Patient/caregiver outcomes: Quality or quantity of social interaction and communication; Mental health morbidity, including stress, anxiety or depression; Quality of life; adverse events; Confidence in, or attributions about, coping; satisfaction with the treatment, dropout, economic outcomes |
| body of evidence | 0 studies |
| AMSTAR2 rating | all critical items met |
| results | Descriptive results  None. |
|  | Quantitative results  None. |
|  | Qualitative results  None. |
|  | Limitations  None. |
|  | Authors conclusion  "Few studies have examined the effectiveness of family therapy for ASD, and none of these are RCTs. Further research studies employing methodologically robust trial designs are needed to establish whether family therapy interventions are clinically beneficial for enhancing communication, strengthening relationships, augmenting coping and reducing mental health morbidity for individuals with ASD and family members" |
| qualitative richness | None (review contains no qualitative statements) |

###### Stahl et al. 2016

| Stahl et al. 2016 | |
| --- | --- |
| study | Stahl, S. T., Rodakowski, J., Saghafi, E. M., Park, M., Reynolds, C. F. & Dew, M. A. (2016). Systematic review of dyadic and family‐oriented interventions for late‐life depression. International Journal Of Geriatric Psychiatry, 31(9), 963–973. https://doi.org/10.1002/gps.4434 |
| design | systematic review of RCTs, narrative synthesis  authors background: Psychiatry, Occupational Therapy, Nursing  investigators participative? no |
| objective | to review the characteristics and findings of dyadic and family-oriented interventions for late-life mood disorders to determine if they are effective and beneficial |
| search period | 201410 |
| participants | psychiatric disorder: late-life depression (≥60 y), dysthymic disorder, bipolar disorder  caregiver type: dyads, family |
| interventions | dyadic or family-oriented psychological, psychosocial, or behavioral interventions |
| controls | not restricted |
| outcomes/ purpose | patient outcomes: not predefined  caregiver outcomes: not predefined |
| body of evidence | 13 articles (10 RCTs)  publication years: 1997 to 2012  countries: USA (n= 8), Germany, Japan, Netherlands  quality rating of the included studies: unclear |
| AMSTAR2 rating | - 3/5 critical items not met - No protocol, no list of excluded studies, quality assessment not reported |
| results | Descriptive results   - scientists background: - participants details   - disorder type: depression/depressive symptoms   - disorder onset/first treatment contact   - caregiver type: close family members (siblings, spouses)   - recruitment: general medical practices, psychiatry departments, rehabilitation centers, memory clinics   - mean age (range): patients 70 years on average; caregivers age reported in 5 studies (mean 69 years)   - percentage women (range): "more patients were women"; no information on caregivers - interventions details   - duration and frequency: 2-12 months   - setting: not reported   - delivery format: family meetings, dyadic psychotherapy, individual counseling/psychotherapy to the patient and support person separately   - type: combination of psychoeducation and some form of psychotherapy for both the patient and support person   - components: problem solving therapy, behavioral activation therapy, ecosystem focused therapy (EFT, a specialized psychotherapy that helps patients learn problem-solving skills and make adjustments in their environment), counseling; dyadic (family) psychotherapy   - personnel: not reported   - follow-up (range): not reported |
|  | Quantitative results  Patient outcomes   - Not summarized, reported for each study separately - studies that aimed to reduce depressive symptom burden 🡪 small effect sizes (r= 0.06–0.15) studies of MDD 🡪 medium effects (r= 0.39–0.41)   caregiver outcomes   - only reported in 1/13 articles (significant effect on support person's depression)   attrition/adherence/drop-out/acceptability: not reported |
|  | Qualitative results  None. |
|  | Limitations  Inclusion not consistently via depression as main diagnosis, but also e.g. via dementia etc.; no comparison of dyadic with individual therapy |
|  | Authors conclusion  „Remarkably few dyadic intervention studies have been attempted to treat depression in older adults. This review showed that dyadic interventions are feasible and that including support persons significantly decreased patients’ depressive symptomatology." |
| qualitative richness | None (review contains no qualitative statements) |

###### Stiawa et al. 2014

| Stiawa et al. 2014 | |
| --- | --- |
| study | Stiawa M, Kilian R & Becker T. Psychosoziale Interventionen bei psychischen Problemen in Familien mit niedrigem sozialem Status--Konzepte und Ergebnisse [Psychosocial interventions for high-risk family members with mental health problems--concepts and results]. Psychiatr Prax. 2014 Sep;41(6):297-304. German. doi: 10.1055/s-0034-1370070. Epub 2014 Sep 2. PMID: 25180590. |
| design | systematic review, all study designs, narrative synthesis  authors background: psychiatry, psychtherapy  investigators participative? no |
| objective | to provide a systematic overview of the effectiveness of psychosocial interventions for mental health problems in families with low socioeconomic status |
| search period | 20121207 |
| participants | psychiatric disorder: mental health problems  caregiver type: children in families with low socioeconomic status (low professional status and/or a low income level and/or a low level of education) in highly developed industrialized countries |
| interventions | complex intervention that consists of more than one component and is aimed at families rather than individuals |
| controls | Not restricted |
| outcomes/ purpose | Not predefined |
| body of evidence | 8 studies (7 RCTs)  publication years: 2004 to 2012  countries: USA (5), UK (1), Canada (1), Australia (1)  quality rating of the included studies: not assessed |
| AMSTAR2 rating | - 4/5 critical items not met - No protocol, no list of excluded studies, no quality assessment, no mentioning of bias risk in discussion - The review does not meet the methodological requirements for systematic reviews. Even if the synthesis is primarily a description of the interventions, at least narrative statements on effectiveness are made, so that an examination of the risk of bias would have been relevant. |
| results | Descriptive results   - scientists background: not reported - participants details: not reported   - disorder type: mostly depression; partly anxiety, addiction   - disorder onset/first treatment contact: not reported   - caregiver type: families   - recruitment: over definition as "low oeconomic status“(i.e. US health insurance for the needy and at-risk groups, governmental financial support, state educational assistance and others)   - mean age (range): not reported   - percentage women (range): in 5/8 studies 100% women/mothers - interventions details:   - duration and frequency: not summarized, very heterogen   - setting: not reported   - individual, group, by telefone, internet-based, home visits   - components: f2f or telefon support, educational (medical, social, psychoeducation), pharmacological therapy, psychotherapy, counselling   - personnel: spezialized nurse, others not reported   - follow-up (range): up to 24 months |
|  | Quantitative results  No synopsis "Interventions were effective in significantly reducing symptoms of depression and anxiety and strengthening social skills."  assessed patient outcomes: "interventions targeted improvement of psychological symptoms, the reduction of risk factors and the strengthening of protective factors against mental illness, as well as reducing access barriers to appropriate psychiatric care for the target group"   - social relationships outcomes   - Role function (Social Adjustment Scale)   - social competence (Short Form 36- Item Health Survey)   - self-confidence (Rosenberg Self-Esteem Scale)   - social support (Social Provisions Scale)   - problem-solving   - coping   - parenting (Parenting Stress IndexShort Form)   - Parent-child relationship - medical utilization and psychological symptoms   - utilization of treatment,   - knowledge on help services   - severity of depression or anxiety   - quality of life   Assessed children's outcomes   - Problem behavior and mental illness in early childhood (Demographics Questionnaire, CBCL, Assessment Protocol) - Symptoms of anxiety disorder (SCAS, CATS)   attrition/adherence/drop-out/acceptability: not reported |
|  | Qualitative results  Families with a low socio-economic status are less able to devote their own time and financial resources resources to make use of adequate psychiatric care, and that and that such care is often not sufficiently available for this group of people in particular  Barriers:   - individual problems (everyday worries, lack of time and financial resources, fear of stigmatization, lack of/cost of transport, lack of childcare, refusal of treatment for mental health problems/prejudices against psychiatrists and psychotherapists) - shortcomings at system level (organization of health services) |
|  | Limitations  Highly heterogeneous interventions and population; poor reporting/synopsis |
|  | Authors conclusion  „Factors such as a person's socio-economic status, e.g. their marital status and level of education, interact with mental illness in such a way that a complex and continuous approach, i.e. a combination of measures over a long period of time, is necessary to improve the situation of affected people in the long term. The intervention studies in this overview show initial hopeful, albeit heterogeneous, results. The complex, often multidisciplinary support services, frequently with a psychosocial focus, which were offered to the participants of the interventions and which mainly provided them with information and a sense of security, should be available to an appropriate extent for people with (a) psychological symptoms and (b) a low socio-economic status in order to improve the situation of those affected with sufficient support in the long term." |
| qualitative richness | Moderate (review contains explicit qualitative statements, even if this was not the primary focus of the study); see chapter results |

###### Sutherland et al. 2020

| Sutherland et al. 2020 | |
| --- | --- |
| study | Sutherland, R., Baker, J. & Prince, S. (2019). Support, interventions and outcomes for families/carers of people with borderline personality disorder: A systematic review. Personality And Mental Health, 14(2), 199–214. https://doi.org/10.1002/pmh.1473 |
| design | systematic review of quantitative studies, narrative synthesis  authors background: Personality Disorder Managed Clinical Network, Faculty of Medicine and Health  investigators participative? no |
| objective | to appraise and synthesize the existing research evidence for interventions for carers of people with bipolar disorder |
| search period | 2007-2019? (search date not reported)  The earliest publication date was set to 2007 to capture studies referenced within and published since the publication of the NICE guidelines in 2009 |
| participants | psychiatric disorder: bipolar disorder (adults or adolescents)  caregiver type: not specified |
| interventions | intervention or combination of interventions involving carers, including (but not restricted to) psychoeducation, peer support and skill-based interventions |
| controls | Not predefined |
| outcomes/ purpose | patient outcomes: none  caregiver outcomes: clinical outcomes for carers, including (but not restricted to) carer burden, carer well-being, mastery and depression |
| body of evidence | 11 studies (2 RCTs, 1 NRCS; 8 non controlled pre-post design), n=511 carers  publication years: 2005 to 2019  countries: 6 USA, 2 Australia, 1 Ireland, 2 UK  quality rating of the included studies: not reported |
| AMSTAR2 rating | - 3/5 critical items not met - No protocol, no quality assessment, no list of excluded studies |
| results | Descriptive results   - scientists background: not reported - participants details: "the gender, age and relationship to the person with BPD of participants were reported differently"   - disorder type: BPD   - disorder onset/first treatment contact: not reported   - caregiver type: "mostly parents", spouses/partners, siblings, (adult) children   - recruitment: not reported   - mean age of carers (range): 18 to 74 years   - percentage women (range): "mostly female", - interventions details   - duration and frequency: 3 to 15 weeks   - setting: not reported   - multi-family group sessions; no individual tailoring   - principles: DBT (3 interventions), cbt, transactional analysis, cognitive analytic therapie, mentalizationsbased therapy (MBT), relational models   - components: psychoeducation, skills development, peer support   - personnel: 4x peer-faciliated or co-faciliated (trained carers, former service patients)   - follow-up (range): 0 to 19 months - interventions names (n=7)   - Family Connections (4 studies)   - Multi-family CBT skills for adolescents (2 studies)   - Oxford Friends Family Empowerment Service (1 study)   - Family Skills (1 study)   - Making Sense of BPB (1 study)   - Staying Connected (1 study)   - MBT-Families and Carers Training and Support (1 study) |
|  | Quantitative results  Patient outcomes   - Impact on person with BPD (symptoms, selfharm, aggression, suicidal threats, fights and withdrawal (3 studies): Child Behaviour Checklist (CBCL), Youth Self-report (YSR), Reynolds Adolescent Depression Scale (RADS), Daily Diary of Critical Incidents 🡪not reliable, because of co-joint treatment of patients and reporting by carers   caregiver outcomes   - Carer burden (objective burden = practical effects of caring such as financial problems; subjective burden = emotional impacts such as guilt or embarrassment; 9 studies): Burden Assessment Scale (BAS); Perceived Burden Scale (PBS) 🡪 Mixed results (benefits or no sig. change) - Carer mental health/Well-being (typically depression; 11 studies): CES-D, BDI-II, SCL-90-R and others 🡪 Mixed results (benefits or no sig. change) - Carer grief (feelings of grief; 4 studies): Grief Assessment Scale (GAS) 🡪 significant decrease: in RCT sig. difference to control group - Carer mastery/empowerment (coping, sense of personal control, sense of family, community empowerment; 6 studies): Mastery Scale, Pearlin Mastery Scale (PMS), Family Empowerment Scale (FES) 🡪 significant increases, but in controlled trials (n=2) no difference to control group - Family Functioning (health of family relationships, distress/problems within family relationships and the level of expressed emotion as Emotional Over-involvement or Criticism; 3 studies): General Functioning subscale of the McMaster Family Assessment Device (FAD), Dyadic Adjustment Scale-4 (DAS-4), The Family Questionnaire (TFQ), Score-15 Index of Family Functioning and Change (SCORE-15) 🡪 Mixed results (benefits or no sig. change) - Knowledge of PD (3 studies): Family-to-Family Outcome Survey (FTF), Personality Disorder Knowledge, Attitudes and Skills Questionnaire (PDKASQ)4, Satisfaction Questionnaire  🡪 significant improvement   attrition/adherence/drop-out/acceptability: not reported   - 10/11 reported on dropout rates, range 1.5% to 57% |
|  | Qualitative results   - None. |
|  | Limitations  lack of control groups, limited follow-up, high dropout rates, incomplete data sets, studies highly heterogeneous, wide variety of outcome measures, poor reporting of effect sizes |
|  | Authors conclusion  „group interventions for carers may lead to some improvements in carer well-being and reductions in carer burden and grief" |
| qualitative richness | None (review contains no qualitative statements) |

###### Sutherland 2023

| Sutherland et al. 2023 | |
| --- | --- |
| study | Sutherland, D., Flynn, S., Kurzeja, O., Griffin, J., Hastings, R. (2023). Family‐systems interventions for families of people with an intellectual disability or who are autistic: a systematic review. Journal Of Intellectual Disability Research, 67(10), 1003–1028. https://doi.org/10.1111/jir.13068 |
| design | Systematic review, all study designs, narrative synthesis  Authors background: Developmental Psychiatry and Psychology  investigators participative? no |
| objective | to summarize what family-systems interventions have been studied with this population, what evidence there is for their effectiveness and families’ experiences of the interventions |
| search period | 202301 |
| participants | psychiatric disorder: intellectual disability (ID), autism or associated genetic syndrome.  caregiver type: Family members of a person with the named disorders. Included were biological, adoptive, foster or stepfamily members.  Note: 2 studies with children and young adolescents as patients |
| interventions | Family-system interventions exclusion: parenting or other interventions that did not target family relationships |
| controls | Not restricted |
| outcomes/ purpose | patient outcomes: any quantitative or qualitative data on experiences of family-systems interventions  caregiver outcomes: any quantitative or qualitative data on experiences of family-systems interventions; wellbeing of family members (life satisfaction, quality of life, mental ill-health or stress), family relationships (family functioning, closeness, quality, supportiveness of relationships associated with these) |
| body of evidence | 11 studies (in 13 reports: 2 RCT, 1 qualitative evaluation of one of the RCTs, 1 NRCT, 3 qualitative studies, 2 pre-post studies, 3 case studies)  n= 292 families (1 to 73)  publication years: 1987 to 2022  countries: UK (n = 4), Hong Kong (n = 4), the Netherlands (n = 2), the USA (n = 2), Spain (n = 1)  quality rating of the included studies: MMAT (1 0/5, 3 1/5, 3 2/5, 2 3/5, 1 4/5) |
| AMSTAR2 rating | - 4/5 critical items not met - No protocol, no list of excluded studies, no quality assessment, quality not mentioned in discussion |
| results | Descriptive results   - participants details   - disorder type: ( 195 ID, 50 autistic)   - disorder onset/first treatment contact: not reported   - caregiver type: Families   - Recruitment: not reported   - mean age (range): non reported; some studies on children/adolescents   - percentage women (range): not reported - interventions details   - duration and frequency: 1-17 months, 1.5h to 3h sessions, 1 to 12 sessions (poor reporting)   - setting: at home, at clinic, university campus, campsite, community setting, school   - delivery format: mostly caregiver(s) + patient in the session, partly only specified members of the family; face-face, online   - type: Family therapy (FT), multisystemic therapy (MST), structural family therapy (SFT), Multi-family group intervention, Multiple family therapy (MFT), Behavioral family therapy (BFT), Systemic Autism-related Family Enabling (SAFE), The Family Quality of Life Support Program, cognitive-behavioral therapy (CBT)   - components/techniques: psychoeducation, systemic and narrative therapy techniques, problem-solving, communication training, crisis prevention and maintenance plan, intra- and interfamilial activities   - manuals: multisystemic therapy – intellectual disability (MST-ID), 2 MST manual (Henggeler et al. 2009), 1 Manual (Martínez et al. 2016), 1 SAFE manual, BFT manual (Falloon et al. 1993)   - personnel: clinical psychologists (n = 4), systemic therapists (n = 4), MST therapists (n = 2), non-systemic therapists (n = 2), unspecified therapists (n = 1), social workers, peers   - follow-up (range): not reported |
|  | Quantitative results  No synopsis, reported for each study seperately  „positive experiences, and important processes, such as changes in family interactions or changes in perceptions of family members with an ID or who are autistic … the effectiveness of family-systems interventions for these populations is unclear … Almost all studies identified some evidence of positive effects of the interventions on family relationships and/or wellbeing"  Patient outcomes:   - Wellbeing - rule breaking behaviour - living at home - symptom reduction (such as reduction in behaviour that challenges)   Caregiver/family outcomes:   - Wellbeing - family relationships - family adaptability - family functioning - caregiver strain - self-esteem   attrition/adherence/drop-out/acceptability: not reported |
|  | Qualitative results   - Themes: (1) sharing care responsibilities among family members, (2) using more open communication styles in the family, (3) mediating family problems, (4) actively involving adolescents with intellectual disabilities in family interactions and (5) guiding the future development of individuals with intellectual disabilities and their families. - improved family relationships, mutual helpful influences occurring among the families and a new understanding of the family members with intellectual disabilities. - Daughter described family therapy as helping her understand what had been going wrong in their family. Parents viewed their daughter more like an adult. - Therapy provided space to voice and resolve concerns. Understood themselves better. Related to extended family in more adaptive way. - therapist as helping refection, increased understanding, feeling closer, more confident to reflect and problem-solve, and improved communication. - programme design, professional practice, and impact and satisfaction with the programme. |
|  | Limitations  Few RCTs, small samples, lack of control groups and methodological weaknesses |
|  | Authors conclusion  “There is a need for higher-quality research to establish whether family-systems interventions are beneficial for families of people who have an ID or who are autistic.” |
| qualitative richness | moderate (review contains explicit qualitative statements, even if this was not the primary focus of the study; see Table 4, p. 1020) |

###### Thompson et al. 2017

| Thompson et al. 2017 | |
| --- | --- |
| study | Thompson, C., De La Cruz, L. F., Mataix-Cols, D. & Onwumere, J. (2017). A systematic review and quality assessment of psychological, pharmacological, and family-based interventions for hoarding disorder. Asian Journal Of Psychiatry, 27, 53–66. https://doi.org/10.1016/j.ajp.2017.02.020 |
| design | systematic review, all study designs, narrative synthesis  authors background: Psychiatry, Psychology and Neuroscience, Psychiatric Research and Education, Health Care Services  investigators participative? no |
| objective | to systematically review the treatments designed to improve HD symptoms and family impact |
| search period | 201601 |
| participants | psychiatric disorder: hoarding disorder  caregiver type: not specified |
| interventions | intervention for individuals or relatives of individuals with HD  note: not limited to interventions involving caregivers |
| controls | Not restricted |
| outcomes/ purpose | patient outcomes: hoarding symptoms or impact on life/psychological distress and others  caregiver outcomes: not predefined |
| body of evidence | (20 studies n= 492 patients + 21 relatives of patients)  2 studies with caregiver involvement, both uncontrolled, n=9, n=12  publication years: 2014, 2016  countries: not reported  quality rating of the included studies: low quality (score 20/100; 21/100) |
| AMSTAR2 rating | - 2/5 critical items not met - No protocol, no list of excluded studies |
| results | **Descriptive results** (of the 2 interventions with caregiver involvement only)   - scientists background: not - participants details:   - disorder type: hoarding disorder   - disorder onset/first treatment contact: not reported   - caregiver type: not reported   - recruitment: not reported   - mean age (range): 46.22 (13.10)/ 40.17 (SD=16. 13) (unclear if patient or caregiver)   - percentage women (range): 75%/78% (unclear if patient or caregiver) - interventions details   - duration and frequency: 14 sessions/7 sessions   - setting: not reported   - delivery format: group sessions,   - type: family-as-motivators’ training (1), family psychoeducation (1)   - components: psychoeducation, harm reduction techniques, training in motivational interviewing, training in reducing family accommodation, problem solving, tips for supporting the patient, promotion of carer wellbeing   - personnel: not reported   - follow-up (range): not reported |
|  | **Qualitative results** (of the 2 interventions with caregiver involvement only)  Patient outcomes   - Chasson et al. 2014: No change in family rated HRS scores - Thompson et al. 2016: none   Caregiver outcomes   - Chasson et al. 2014: improvement in Family Response to Hoarding Scale + Family Member Impact Scale, some positive changes in coping style and a measure of knowledge about HD; no change on the Quality of Life Scale - Thompson et al. 2016: no changes in carer anxiety, depression, burden associated with HD or coping style; improvement in well-being (Warwick-Edinburgh Mental Well-being Scale)   attrition/adherence/drop-out/acceptability:   - Chasson et al. 2014: 4/9 (44%) dropout before mid-treatment - Thompson et al. 2016: 1/12 (8%) post-treatment (further 3 lost at follow-up, 4/12 (29%) at treatment |
|  | Qualitative results  None. |
|  | Limitations  Few studies on caregivers, no comparative studies (pre-post only), high attrition |
|  | Authors conclusion  „This review also included two papers looking at interventions for relatives of people with HD. Both studies reviewed here had small sample sizes but showed promising results in reducing the impact on the family and improving understanding of HD" |
| qualitative richness | None (review contains no qualitative statements) |

###### Thompson-Hollands et al. 2014

| Thompson-Hollands et al. 2014 | |
| --- | --- |
| study | Thompson-Hollands, J., Edson, A., Tompson, M. C. & Comer, J. S. (2014). Family involvement in the psychological treatment of obsessive–compulsive disorder: A meta-analysis. Journal Of Family Psychology, 28(3), 287–298. https://doi.org/10.1037/a0036709 |
| design | systematic review, all study designs, narrative synthesis  authors background: Psychology  investigators participative? no |
| objective | To evaluate (a) the overall effect of the range of family inclusive treatment (FIT) on OCD, as well as (b) moderators of treatment outcome that can inform for whom and under what circumstances FITs may be most effective for patients with OCD |
| search period | 201205 |
| participants | psychiatric disorder: obsessive-compulsive disorder (OCD) [note: SR includes children/adolscents and adult samples]  caregiver type: family |
| interventions | some level of standardized family involvement |
| controls | Not restricted |
| outcomes/ purpose | patient outcomes: effects of moderators on OCD symptoms and functioning  caregiver outcomes: |
| body of evidence | 29 studies, n= 1366  7 studies on adult patients (mean age ≥ 18)  publication years: 1990 to 2013  countries: not reported  quality rating of the included studies: not assessed |
| AMSTAR2 rating | - 4/5 critical items not met - No protocol, no list of excluded studies, no quality assessment, risk of bias not mentioned in discussion |
| results | Descriptive results   - scientists background: not reported - participants details   - disorder type: obsessive compulsive disorder   - disorder onset/first treatment contact: not reported   - caregiver type: mostly parents; for adult studies not reported   - recruitment: not reported   - mean age (range): s 17.89 {SD = 9.72; range 7.1-37.0)   - percentage women (range): not reported - interventions details   - duration and frequency: 1-24 session (often less sessions for caregivers)   - setting: not reported   - delivery format: group, individual family   - components: psychoeducation and exposure-based treatment (100%); instructions to family members to reduce accommodation (83%); family training as a "coach“for exposures (45%)   - personnel: not reported   - follow-up (range): not reported - Level of family involvement: - 22 included families for at least a portion of all sessions with the patient; - 7 studies included families for the all sessions in full - 5 studies included families for only one session. - studies that listed a manual for the family intervention: 19 studies, majority used or adapted the treatment manual by March and Mulle (1998) |
|  | Quantitative results  Patient outcomes   - OCD symptoms (K = 28, pooled d = 1.68, SE = .14, 95% CI [1.41, 1.95], z = 12.23, p < .001) - between-groups effects (K = 12, pooled d = 1.45, SE = .29, 95% CI [.89, 2.01], z = 5.06, p < .001) - functioning (within-groups effects; K = 9, pooled d = .87, SE = .11, 95% CI [.65, 1.10], z = 7.63, p < .001; between-groups effects; K = 6, pooled d= 1.21, SE = .32, 95% CI [.58, 1.83], z = 3.77, p < .001) - moderators for OCD symptoms: number of sessions - no moderators: age group (<18/≥ 18), ethnicity, gender, delivery format (group/individual family), level of family involvement - moderators for functioning: gender (males greater improvements); delivery format (individual therapy with larger effects)   caregiver outcomes   - None.   attrition/adherence/drop-out/acceptability: not reported |
|  | Qualitative results  None. |
|  | Limitations  Most studies on non-adult patients; heterogeneity across FITs, as well as the small number of trials, no quality assessment |
|  | Authors conclusion  „Results indicate a robust overall response to FITs for OCD and clarify key moderators that inform optimal circumstances for effective treatment " |
| qualitative richness | None (review contains no qualitative statements) |

###### Van Es et al. 2023

| Van Es et al. 2023 | |
| --- | --- |
| study | Van Es, C. M., Khoury, B. E., Van Dis, E. A. M., Brake, H. T., Van Ee, E., Boelen, P. A. & Mooren, T. (2023). The effect of multiple family therapy on mental health problems and family functioning: A systematic review and meta‐analysis. Family Process, 62(2), 499–514. https://doi.org/10.1111/famp.12876 |
| design | systematic reviews of controlled trials, meta-analysis  authors background: Psychotrauma Centres, Centre of Expertise for the Impact of Disasters and Crises …  investigators participative? no |
| objective | To provide an overview of existing controlled trials focusing on the impact of multiple family therapy (MFT) on mental health problems and family functioning. |
| search period | 202106 |
| participants | psychiatric disorder: mental health problems  caregiver type: family |
| interventions | multiple family therapy (Laqueur 1964): two or more families, including the patient and at least one family member, together representing at least two generations, take part. (definition by O'Shea and Phelps (1985), to distinguish MFT from other therapeutic modalities and techniques, such as group therapy or single-family therapy) |
| controls | Not restricted |
| outcomes/ purpose | patient outcomes: mental health problems  caregiver outcomes: family functioning |
| body of evidence | 31 studies, n=4760 (16 in meta-analysis); 19 RCTs  countries: United States (52%), Western Europe (19%), China/Hong Kong (16%), Australia (6%)  quality rating of the included studies: 1 low, 11 moderate, 4 high risk of bias |
| AMSTAR2 rating | - 1/7 critical items not met - No list of excluded studies |
| results | Descriptive results   - scientists background: not reported - participants details   - disorder type: mostly schizophrenia/psychosis (k = 9; 29%), mood problems such as depressive disorders (k = 5; 16%), and conduct problems such as disruptive behavior (k = 5; 16%) and other (somatic problems incl.: cancer, diabetes, cognitive impairment, minorities)   - disorder onset/first treatment contact: not reported   - caregiver type: children (55%) + caregivers/siblings; 45% adults + care dyad and/or key relative (i.e., spouse, child, friend, and parent)   - recruitment: not reported   - mean age (range): not reported   - percentage women (range): not reported for all studies; in reported studies: 43% female - interventions details   - duration and frequency: ranging from 6 weekly sessions to biweekly meetings for 2 years   - type: multi-family therapy/Psychoeducation   - psychoeducational MFT (McFarlane et al., 1995, 2002): 5 studies   - Families and Schools Together (FAST, McDonald (2002): 5 studies   - 4Rs 2Ss“(rules, responsibility, relationships, respectful communication, stress, and social support) (McKay et al., 1999)   - components: adaptation to illness, coping skills, social support, illness management skills. Other techniques: behavioral parenting interventions, social skills training, cognitive-behavioral techniques   - use of a manual: k = 22; 71%   - MFT was offered in combination (pharmacological treatment, medical care, treatment-as-usual/routine outpatient care): . k = 19; 61% |
|  | Quantitative results  Patient outcomes:   - positive and/or negative psychotic symptoms: g = 0.96, 95% CI [−0.19, 2.11]; I^2^=97%; 6 studies - mood problems: g= 0.05, 95% CI [−0.34, 0.44]; I^2^=67%, 4 studies   family outcomes:   - family functioning: g= 0.26, 95% CI [0.05, 0.51]; I^2^=50%; 7 studies - used instruments for family functioning: CFI = Camberwell Family Interview, EEAC = Expressed Emotion Adjective Checklist, FACES = Family Adaptability and Cohesion Evaluation Scale, FAD = Family Assessment Device, FMSS = Five-Minute Speech Sample, GFFS = General Family Functioning Scale, LEE = Level of Expressed Emotion - "It remains unclear in what way MFT is different from individual and other forms of family therapy“(Gelin et al., 2016).   attrition/adherence/drop-out/acceptability: not reported |
|  | Qualitative results  None. |
|  | Limitations  Focus on MFT, resulting in an extremely heterogen population: not only mental disorders, 55% on children; all PICO categories very heterogen; for meta-analyses only few studies, high statistical heterogeneity. |
|  | Authors conclusion  „To conclude, more methodologically rigorous research is needed to further examine the potential benefits of MFT, as well as the working mechanisms and core components of MFT." |
| qualitative richness | None (review contains no qualitative statements) |

###### Wang et al. 2022

| Wang et al. 2022 | |
| --- | --- |
| study | Wang, Y., Chen, Y. & Deng, H. (2022). Effectiveness of Family- and Individual-Led Peer Support for People With Serious Mental Illness: A Meta-Analysis. Journal Of Psychosocial Nursing And Mental Health Services, 60(2), 20–26. https://doi.org/10.3928/02793695-20210818-01 |
| design | systematic review of RCTs, meta-analysis  authors background: Mental Health Center, Clinical Medical Research Center for Mental Disorders, Hope Recovery and Rehabilitation Center 🡪 Journal of Nursing  investigators participative? No |
| objective | assessing the value and role of peer support interventions to improve outcomes in people with SMI |
| search period | 2021902 |
| participants | psychiatric disorder: Serious Mental Illness (schizophrenia, schizoaffective disorder, psychotic disorder not otherwise specified, bipolar disorder, or major depressive disorder)  caregiver type: family members |
| interventions | Family- and Individual-Led Peer Support |
| controls | Not restricted |
| outcomes/ purpose | patient outcomes: not predefined  caregiver outcomes: not predefined |
| body of evidence | 28 articles (5 family-led peer support, 23 individual-led peer support)  countries 4x Hong Kong, 1x USA  quality rating of the included studies: low RoB |
| AMSTAR2 rating | - 4/7 critical items not met - No protocol, no list of excluded studies, risk of bias not mentioned in discussion, publication bias not assessed |
| results | **Descriptive results** (studies on family-led peer support only)   - scientists background: not reported - participants details: not summarized   - disorder type: 4x schizophrenia, 1x miscellaneous - interventions details: not summarized;   - peer support categories: self-help groups, internet support groups, services provided by peers, services run or operated by peers, peer partnerships, peer employees (Solomon, 2004) |
|  | **Quantitative results** (studies on family-led peer support only)  Patient outcomes   - level of psychosocial functioning (Specific Level of Functioning Scale (Schneider & Struening 1983) - SMD = –2.47, 95% CI [–2.95, –1.98]; I^2^=68%; 3 studies, n=407 - psychiatric symptoms: SMD = –1.45, 95% CI [–2.68, –0.22]; I^2^=98%; 5 studies, n=742 - rehospitalization: SMD = –1.34, 95% CI [–1.94, –0.75]; I^2^=87%; 4 studies, n=483 - duration - SMD = –1.48, 95% CI [–2.56, –0.41]; I2=96%; 4 studies, n=483   Family outcomes   - family function (multiple dimensions; Family Assessment Device (Miller et al., 1985) - SMD = 0.9, 95% CI [–0.50, 2.30; I^2^=98%; 4 studies, n=646 - family burden (Family Burden Interview Schedule (Pai & Kapur, 1982; Family Experiences Interview Schedule (U.S. Department of Health and Human Services, 1993) - SMD = –1.75 (95% CI –3.63, 0.12); I^2^=99%; 3 studies, n=540 - use of formal community support services (Family Support Service Index (Heller & Factor, 1991) - SMD = –1.38, 95% CI [–2.19, –0.56]; I^2^= 93%; 4 studies, 481   attrition/adherence/drop-out/acceptability: not reported |
|  | Qualitative results  None. |
|  | Limitations  components of peer support for interventions differ, scales used for the same aspects are inconsistent, high statistical heterogeneity, follow-up time was inconsistent, different intervention locations (in- and outpatient) |
|  | Authors conclusion  „These results suggest that peer support, as a potential resource, can be further developed for the rehabilitation of those with SMI, despite limited available evidence" |
| qualitative richness | None (review contains no qualitative statements) |

###### Zinser et al. 2022

| Zinser et al. 2022 | |
| --- | --- |
| study | Zinser, J., O’Donnell, N., Hale, L. & Jones, C. J. (2022). Multi‐family therapy for eating disorders across the lifespan: A systematic review and meta‐analysis. European Eating Disorders Review, 30(6), 723–745. https://doi.org/10.1002/erv.2919 |
| design | systematic review, all study designs, meta analysis  authors background: Psychology  investigators participative? no |
| objective | To determine the impact of MFT on patient's weight and ED symptomatology, as well as on patient and carer psychological well‐being, To determine the impact of MFT on family functioning and caregiver burden, To determine the acceptability of MFT as an intervention for EDs, and the acceptability of outcome measures utilised. |
| search period | 202104 |
| participants | psychiatric disorder: eating disorders  caregiver type: family member or person of support |
| interventions | Multi‐family therapy |
| controls | Not restricted |
| outcomes/ purpose | Not predefined |
| body of evidence | 15 studies (2 RCTs), n=850 (patients)  countries: 4xUK, 3xUSA, 2xCanada, 2xBelgium, 1xSweden, 1xDenmark, 1xCzech Republic, 1xNorway  quality rating of the included studies: 2 RCTs: strong (RoB: some concerns), 8 moderate, 5 weak; cave: barriers to blinding |
| AMSTAR2 rating | - 1/7 critical items not met - No list of excluded studies |
| results | Descriptive results   - scientists background: not reported - participants details   - disorder type: eating disorder   - disorder onset/first treatment contact: not reported   - caregiver type: family (not reported in detail)   - recruitment: not reported   - age: 5 studies adults, 8 adolescents, 1 adolescents+young adults; range 14 to 26   - **u**Gender reported in 14/15 studies; % female 90% to 100%   - Information on participating family members reported in 9 studies: mothers largest proportion (range 32% to 57%) - interventions details   - duration and frequency: 3-12 months; contact hours app. 12-147 h   - setting: 2x inpatient, 10 outpatient, 2 both   - components: "MFT typically involves three to eight families attending a 3‐ or 4‐day intensive programme followed by monthly sessions over 6–9 months. Families may have additional single‐family sessions in between these as needed. Sessions last for 1 day, including a family meal plus snacks, and are facilitated by two to four clinicians. However, variability does exist …“(pp. 724.725). |
|  | Quantitative results  Patient outcomes   - weight SMD = 0.12, (95% CI = −0.42, 0.67); 4 comparison studies, n=283 not sig. - weight SMD = 0.85, (95% CI = 1.25, 0.46); I2=80%; 8 pre-post-studies, n=630 - adults: SMD = 0.18, (95% CI = −0.09, 0.45), I2=0%; 3 pre-post-studies, n=215 not sig. - adolescents: SMD = 1.15, (95% CI = 0.94, 1.36), I2=0%; 5 pre-post-studies, n=415 - eating disorder symptoms (restraint, eating concern, shape concern, weight concern; EDE-Q):  MD −0.83, (95% CI = −1.23, −0.44), I2=20%; 6 studies, n=272 - patients' report of depression symptoms: SMD = −0.59, (95% CI = −0.88, −0.31), 4 studies, n=214 - patients' general psychological well‐being: SMD = 1.09, (95% CI = 0.81, 1.38), 3 studies, n=222   Family outcomes   - Family functioning: SMD −0.3, (95% CI = −0.78, 0.18), 2 studies, n=229 not sig. - negative caregiving appraisals: SMD = 0.06, (95% CI = −0.18, 0.30), 4 studies, n=274 not sig. - positive caregiving experiences: SMD = 0.05, (95% CI = −0.18, 0.29), 4 studies, n=257 not sig.   attrition/adherence/drop-out/acceptability:   - completion rates (range) 86%-100% (follow-up data: 31-81%) |
|  | Qualitative results  None. |
|  | Limitations  Wide‐ranging variation in the MFT model, the length and structure of interventions, follow‐up periods, and outcome measures, study quality weak to moderate, many studies without comparison group, no blinding, possible selection bias. |
|  | Authors conclusion  "significant improvements were only evident when comparisons were drawn before and after the intervention; these dissipated when MFT was compared to another intervention. There was no evidence MFT improves family functioning, positive aspects of caregiving, nor patient and parental anxiety". |
| qualitative richness | None (review contains no qualitative statements) |

# S 7 Matrix of included primary studies in evidence syntheses on schizophrenia

| **primary study** | **#** | **Rodolico 2022** | **Al-Sawafi 2020** | **Ashcroft 2018** | **Okpokoro 2014** | **Bademli 2011** | **Macleod 2010** | **Pharoah 2010** |
| --- | --- | --- | --- | --- | --- | --- | --- | --- |
| **comment** | included in x/7 SRs | focus on relapse data | only studies from the Arab world included | Caregiver-directed psycho-social inter-ventions | focus on brief interven-tions, focus on user outcomes | focus on peer-led interven-tions (family to family) | focus on nurse-led interven-tions | focus on user outcomes |
| Is the review containing a list of excluded studies (AMSTAR2 )? |  | y | n | n | y | n | n | y |
| Abramowitz 1989 | 1 |  |  |  |  |  | x |  |
| Addington 2005 | 1 |  |  |  |  |  | x |  |
| Aguglia 2007 | 1 | x |  |  |  |  |  |  |
| Ahmed 2018 | 1 |  | x |  |  |  |  |  |
| Al-Hadi 2017 | 1 |  | x |  |  |  |  |  |
| Asberg-Wisted 1995 | 1 |  |  |  |  |  | x |  |
| Barber 1988 | 1 |  |  |  | x |  |  |  |
| Barrowclough 1987 | 1 |  |  |  |  |  | x |  |
| Barrowclough 1999 | 2 | x |  |  |  |  | x |  |
| Barrowclough 2001 | 1 |  |  |  |  |  |  | x |
| Bentley 1990 | 1 |  |  |  |  |  | x |  |
| Berglund 2003 | 1 |  |  |  |  |  | x |  |
| Berkowitz 1990 | 1 |  |  |  |  |  | x |  |
| Bertrando 2006 | 1 | x |  |  |  |  |  |  |
| Bhugra 2011 | 1 | x |  |  |  |  |  |  |
| Bloch 1995 | 1 |  |  |  |  |  |  | x |
| Bradley 2006 | 3 |  |  | x |  |  | x | x |
| Bressi 2008 | 2 | x |  | x |  |  |  |  |
| Brooker 1992 | 1 |  |  |  |  |  | x |  |
| Brooker 1994 | 1 |  |  |  |  |  | x |  |
| Buchkremer 1995 | 2 | x |  |  |  |  |  | x |
| Caqueo-Urizar 2007 | 1 |  |  |  |  |  | x |  |
| Carpenter 1986 | 1 | x |  |  |  |  |  |  |
| Carpentier 1992 | 1 |  |  |  |  |  | x |  |
| Carra 2007 | **4** | x |  | x |  |  | x | x |
| Cassidy 2001 | 1 |  |  |  |  |  | x |  |
| Cazzullo 1989 | 1 |  |  |  |  |  | x |  |
| Cechnicki 2017 | 1 | x |  |  |  |  |  |  |
| Chatterjee 2014 | 2 | x |  | x |  |  |  |  |
| Chan 2008 | 1 |  |  |  |  |  | x |  |
| Chen 2000 | 1 | x |  |  |  |  |  |  |
| Chen 2005 | 1 |  |  |  |  |  |  | x |
| Cheng 2005 | 1 |  |  |  |  |  | x |  |
| Chien/Norman/Thompson 2004 | 3 |  |  |  |  | x | x | x |
| Chien/Chan 2004 | 1 |  |  |  |  | x |  |  |
| Chien/Chan/Morrissey 2005 | 2 |  |  |  |  | x | x |  |
| Chien/Chan/Thompson 2006 | 2 |  |  |  |  | x | x |  |
| Chien 2008 | 1 |  |  |  |  | x |  |  |
| Chien/Thompson/Norman 2008 | 1 |  |  |  |  | x |  |  |
| Chou 2002 | 1 |  |  |  |  |  | x |  |
| Cozolino 1988 | 1 |  |  |  |  |  | x |  |
| Craig 2004 | 1 | x |  |  |  |  |  |  |
| Creed 1997 | 1 |  |  |  |  |  | x |  |
| Dai 2007 | 2 |  |  | x |  |  |  | x |
| De Giacomo 1997 | 2 | x |  |  |  |  |  | x |
| Dixon 2001 | 1 |  |  |  |  | x |  |  |
| Dixon 2004 | 1 |  |  |  |  | x |  |  |
| Doane 1986 | 1 |  |  |  |  |  | x |  |
| Drury 1996 | 1 | x |  |  |  |  |  |  |
| Du 2005 | 1 |  |  |  |  |  |  | x |
| Dyck 2002 | 1 |  |  |  |  |  |  | x |
| El-Shafei 2008 | 2 | x | x |  |  |  |  |  |
| Falloon 1981, 82, 85, 87 … | 2 |  |  |  |  |  | x | x |
| Fernandez 1998 | 1 |  |  |  |  |  |  | x |
| Garety 2008 | 1 | x |  |  |  |  |  |  |
| Giron 2010 | 2 | x |  | x |  |  |  |  |
| Glynn 1992 | 2 | x |  |  |  |  |  | x |
| Goldstein 1981 | 2 | x |  |  |  |  |  | x |
| Gong 2007 | 1 |  |  |  |  |  |  | x |
| Grawe 2006 | 2 | x |  | x |  |  |  |  |
| Guo 2007 | 2 | x |  |  |  |  |  | x |
| Guo 2010 | 2 | x |  | x |  |  |  |  |
| Gutierrez-Maldonado 2007 | 1 |  |  |  |  |  | x |  |
| Haddock 2003 | 1 |  |  |  |  |  | x |  |
| Harvey 2002 | 1 |  |  |  |  |  | x |  |
| Hasan 2014 | 1 |  | x |  |  |  |  |  |
| Hasan 2015 | 2 | x |  | x |  |  |  |  |
| Hazel 2004 | 1 |  |  |  |  |  | x |  |
| Herz 2000 | 2 | x |  |  |  |  |  | x |
| Hogarty 1986 | 2 | x |  |  |  |  |  | x |
| Hogarty 1997 | 2 | x |  |  |  |  |  | x |
| Hornung 1996 | 1 | x |  |  |  |  |  |  |
| Hoult 1984 | 1 |  |  |  |  |  | x |  |
| Hu 1994 | 1 | x |  |  |  |  |  |  |
| Hugen 1993 | 1 |  |  |  |  |  | x |  |
| Jenner 2004 | 1 | x |  |  |  |  |  |  |
| Jeppesen 2005 | 1 |  |  |  |  |  | x |  |
| Jiang 2007 | 1 | x |  |  |  |  |  |  |
| Kane 1990 | 1 |  |  |  |  |  | x |  |
| Kane 2016 | 1 | x |  |  |  |  |  |  |
| Klingberg 2010 | 1 | x |  |  |  |  |  |  |
| Köttgen 1998 | 1 | x |  |  |  |  |  |  |
| Kopelowicz 2003 | 2 | x |  |  |  |  | x |  |
| Kopelowicz 2012 | 1 | x |  |  |  |  |  |  |
| Koukia 2005 | 1 |  |  |  |  |  | x |  |
| Kuipers 2004 | 1 | x |  |  |  |  |  |  |
| Kulhara 2009 | 2 | x |  | x |  |  |  |  |
| Leavey 2004 | 1 |  |  |  |  |  |  | x |
| Leff 1982 | 2 | x |  |  |  |  |  | x |
| Leff 1989 | 2 | x |  |  |  |  |  | x |
| Leff 2001 | 3 | x |  |  |  |  | x | x |
| Li 2004 | 2 | x |  |  |  |  |  | x |
| Li 2005a (n=101) | **3** | x |  |  |  |  | x | x |
| Li 2005b (n=80) | 1 | x |  |  |  |  |  |  |
| Li 2008 | 1 | x |  |  |  |  |  |  |
| Lintzen 1996 | 1 |  |  |  |  |  |  | x |
| Liu 2003 | 1 |  |  |  |  |  |  | x |
| Liu 2007 | 2 | x |  |  |  |  |  | x |
| Ling 1999 | 1 | x |  |  |  |  |  |  |
| Luo 2019 | 1 | x |  |  |  |  |  |  |
| Luping 2007 | 1 |  |  |  |  |  |  | x |
| Lv 2003 | 2 | x |  |  |  |  |  | x |
| Ma 2003 | 1 | x |  |  |  |  |  |  |
| Magliano 2005 | 1 |  |  |  |  |  | x |  |
| Magliano 2006 | 2 |  |  |  |  |  | x | x |
| Mak 1997 | 2 | x |  |  |  |  |  | x |
| Marchira 2017 | 1 | x |  |  |  |  |  |  |
| Mayoral 2015 | 1 | x |  |  |  |  |  |  |
| McDonell 2003 | 1 |  |  |  |  |  | x |  |
| McDonell 2006 | 1 |  |  | x |  |  |  |  |
| McDonell 2007 | 1 |  |  |  |  |  | x |  |
| McFarlane 1995a (n=34) | 1 | x |  |  |  |  |  |  |
| McFarlane 1995b (n=172) | 2 | x |  |  |  |  |  | x |
| McGill 1983 | 1 |  |  |  |  |  | x |  |
| Merinder 1999 | 3 | x |  |  |  |  | x | x |
| Montero 1999 | 1 | x |  |  |  |  |  |  |
| Mueser 2001 | 1 |  |  |  |  |  | x |  |
| Nasr 2009 | 1 |  |  | x |  |  |  |  |
| NCT02911519 | 1 | x |  |  |  |  |  |  |
| Orhagen 1992 | 1 |  |  |  |  |  | x |  |
| Pai 1982 | 1 |  |  |  |  |  | x |  |
| Palma 2019 | 1 | x |  |  |  |  |  |  |
| Palm-Sevillano 2011 | 1 |  |  | x |  |  |  |  |
| Petersen 2005 | 1 | x |  |  |  |  |  |  |
| Pickett-Schenk/Cook... 2006 | 1 |  |  |  |  | x |  |  |
| Pickett-Schenk/Benett … 2006 | 1 |  |  |  |  | x |  |  |
| Pickett-Schenk 2008 | 1 |  |  |  |  | x |  |  |
| Pitschel-Walz 2006 | 1 | x |  |  |  |  |  |  |
| Piyavhatkul 2017 | 1 | x |  |  |  |  |  |  |
| Pharoah 2010 | 1 |  |  | x |  |  |  |  |
| Posner 1992 | 3 | x |  |  |  |  | x | x |
| Qiu 2002 | 1 | x |  |  |  |  |  |  |
| Pu 2003 | 1 | x |  |  |  |  |  |  |
| Qiu 2005 | 1 |  |  |  |  |  |  | x |
| Rami 2018 | 1 |  | x |  |  |  |  |  |
| Ran 2003 | 3 | x |  |  |  |  | x | x |
| Randolph 1994 | 1 |  |  |  |  |  |  | x |
| Rao 1988 | 1 |  |  |  |  |  | x |  |
| Razali 2000 | 2 | x |  |  |  |  | x |  |
| Reynolds 1984 | 1 |  |  |  |  |  | x |  |
| Rotondi 2005 | 1 |  |  |  |  |  | x |  |
| Schooler 1997 | 2 | x |  |  |  |  |  | x |
| Secher 2015 | 1 |  |  | x |  |  |  |  |
| Sellwood 2001 | 1 |  |  |  |  |  | x |  |
| Sellwood 2007 | 1 |  |  | x |  |  |  |  |
| She 2017 | 1 | x |  |  |  |  |  |  |
| Shi 2000 | 1 |  |  |  |  |  |  | x |
| Shimodera 2000 | 1 | x |  |  |  |  |  |  |
| Shinde 2005 | 1 |  |  |  | x |  |  |  |
| Shiraishi 2019 | 1 | x |  |  |  |  |  |  |
| Shore 1998 | 1 |  |  |  |  |  | x |  |
| Sidley 1991 | 1 |  |  |  |  |  | x |  |
| Smith 1987 | 2 |  |  |  | x |  | x |  |
| So 2006 | 1 |  |  |  |  |  | x |  |
| Soliman 2018 | 1 |  | x |  |  |  |  |  |
| Solomon 1997 | 1 |  |  |  |  | x |  |  |
| Sota 2008 | 1 |  |  |  |  |  | x |  |
| Spiegel 1987 | 1 | x |  |  |  |  |  |  |
| Stengard 2003 | 1 |  |  |  |  |  | x |  |
| Sungur 2011 | 2 | x |  | x |  |  |  |  |
| Szmukler 1996 | 1 | x |  |  |  |  |  |  |
| Szmukler 1998 | 1 |  |  |  |  |  | x |  |
| Szmukler 2003 | 2 | x |  |  |  |  |  | x |
| Tan 2005 | 1 | x |  |  |  |  |  |  |
| Tan 2007 | 2 | x |  |  |  |  |  | x |
| Tarrier 1988a (n=64) | 2 | x |  |  |  |  |  | x |
| Tarrier 1988b (n=19) | 1 | x |  |  |  |  |  |  |
| Tee 2000 | 1 |  |  |  |  |  | x |  |
| Test 1980 | 1 |  |  |  |  |  | x |  |
| Turkington 2002 | 1 |  |  |  |  |  | x |  |
| Valencia 2007 | 1 | x |  |  |  |  |  |  |
| Valencia 2013 | 2 | x |  | x |  |  |  |  |
| Vaughan 1992 | 2 | x |  |  |  |  |  | x |
| Veltro 2006 | 2 | x |  |  |  |  | x |  |
| Vickar 2009 | 1 | x |  |  |  |  |  |  |
| Wang 1997 | 1 | x |  |  |  |  |  |  |
| Wang 2005 | 1 | x |  |  |  |  |  |  |
| Wang 2006 | 1 |  |  |  |  |  |  | x |
| Winefield 1995/1998 | 1 |  |  |  |  |  | x |  |
| Xiang 1994 | 1 |  |  |  |  |  |  | x |
| Xiang 2005 | 1 |  |  |  |  |  |  | x |
| Xiong 1994 | 3 | x |  |  |  |  | x | x |
| Yamaguchi 2006 | 1 |  |  |  |  |  | x |  |
| Yi 2006 | 1 | x |  |  |  |  |  |  |
| Youssef 1987 | 1 |  |  |  | x |  |  |  |
| Zastowny 1992 | 1 | x |  |  |  |  |  |  |
| Zhang 1994 | 2 | x |  |  |  |  |  | x |
| Zhang 1998 | 1 |  |  |  |  |  | x |  |
| Zhang 2003 | 1 | x |  |  |  |  |  |  |
| Zhang 2006a (n=150) | 2 | x |  |  |  |  |  | x |
| Zhang 2006b (n=60) | 2 | x |  |  |  |  |  | x |
| Zhao 2001 | 1 | x |  |  |  |  |  |  |
| Zhou 2007 | 1 |  |  |  |  |  |  | x |
| Zhu 1998 | 1 | x |  |  |  |  |  |  |
| **n of included studies (x/194)** |  | 94 | 6 | 18 | 4 | 12 | 67 | 53 |
| **included in 1/7 SRs** | 142 |  |  |  |  |  |  |  |
| **included in 2/7 SRs** | 43 |  |  |  |  |  |  |  |
| **included in 3/7 SRs** | 8 |  |  |  |  |  |  |  |
| **included in 4/7 SRs** | 1 |  |  |  |  |  |  |  |
| **included in 5/7 SRs** | 0 |  |  |  |  |  |  |  |
| **included in 6/7 SRs** | 0 |  |  |  |  |  |  |  |
| **included in 7/7 SRs** | 0 |  |  |  |  |  |  |  |

# S8 Description of effectiveness of a selection of next of kin interventions

Certainty of evidence was low to very low, due to heterogeneity (of participants, interventions, controls, outcome measures, and results), risk of bias, and imprecision (few studies and participants, wide confidence intervals) of the ES included. 16 of the included ES conducted a meta-analysis, with only 9 analysing the effects on NOK, with different quality, making any generalization difficult.

Five examples for ES with a good reporting quality are reported in the following, including a meta-analysis with a high certainty of evidence and examples that explain the effects, suggesting that interventions for the involvement of NOK may result in moderate to large effect sizes for improving family functioning (52, 55), the reduction of psychological distress (52, 60) and the burden of NOK (55), with similar effects for individual and group interventions. Two other studies describe the reduction in depressive symptoms of NOK (60, 61) and one meta-analysis (60) positive effects on expressed emotions, NOK well-being, their global morbidity and depressive symptoms too. The most certain effects are related to psychoeducation, but only because most other interventions have not been studied individually.

In relation to PIT outcomes, various types of involvement of NOK seemed to result in a reduced relapse rate (35), whereas potential mediators for these effects are supportive relationships between NOK and PIT (41) and educational components (35), other moderating effects having sparsely been investigated. These results are almost exclusively based on indirect comparisons and mediator analyses, making the certainty of evidence is very low. However, randomized double-blind studies for the direct comparison of interventions are unlikely to be feasible in this field.

- **Hansen 2020:** A meta-analysis of mental health interventions for *informal caregivers* of people with severe mental illness found moderate effect sizes for the reduction of psychological distress (SMD 0.58, 95% CI -1.19 to 0.16; I^2^=91; 15 RCTs, n=823). Selected were patients without restrictions in relation to the mental health problems. The analysis demonstrates evidence that either individual or group formats and manualized interventions ≥ 8 weeks are effective, while non-manualized interventions <8 weeks are not.
- **Rushton 2023**: A meta-analysis of psychosocial interventions for *family members* of people with substance use disorder found moderate effects on depression (SMD 0.50, 95% CI 0.21 to 0.79) and small effects on distress (SMD 0.28, 95% CI 0.03 to 0.54). However, this was based on 3 RCTs only. For all other outcomes no evidence was found.
- **Sin 2017**: A review of psychoeducational interventions for *family carers* of people with psychosis found small effects on carers wellbeing (SMD 0.103, 95% CI −0.186 to 0.392, I2=0%, 2 RCTs, n=184 carers), stress (SMD −0.169, 95% CI −0.410 to 0.072, I2= 0%, 4 RCTs, n = 266 carers), negative caregiving experiences (SMD −0.210, 95% CI −0.396 to −0.025, I2=0%, 5 RCTs, n =446), and expressed emotions (SMD −0.161, 95% CI −0.367 to −0.045, I2=0%, 5 RCTs, n= 3379) as well as small to moderate effects on perceived burden (SMD −0.434, 95% CI −0.567 to −0.31, I2=0%, 10 RCTs, n = 878). The effect on burden increased with longer follow-up (6 months SMD −1.628, 95% CI−2.307 to−0.948, I2= 94.5%, 10 RCTs, n = 821).
- **Ma 2017**: A review on *family intervention* *for caregivers* *of people with* for recent onset psychosis found significant effects on care burden to all timepoints with increasing effect sizes by time (4 RCTs). For family functioning, there were significant effects only at >6 month, and for formal support services utilization there were significant effect first at >12 month of intervention.
- **Rodolico 2022**: A large review on *family interventions* in schizophrenia focused on relapse rates as main outcome. At 12 months, 37% of patients treated as usual relapsed. Simple forms of family psychoeducation resulted in a relapse rate of 9,7% (OR 0,18; 95% CI 0,12 to 0,27; 9 comparisons). For systemic family-oriented interventions, the relapse rate was 16,3% (OR 0,33, 95% CI 0,17 to 0,66; 4 comp.). More complex forms of family psychoeducation (i.e. including skills training) achieved relapse rates between 17,1% and 22,4%; pure next of kin psychoeducation interventions resulted in relapse rates between 21,8% and 23,2%; while community-based care interventions (27%; OR 0,63) and brief family psychoeducation (32,8%; OR 0,83) were less effective. For family psychoeducation, they estimate the certainty of evidence as moderate, with downgrading due to risk of bias (no blinding of participants and personnel).

# S9 Effects on next of kin according to the ES with meta-analyses on next of kin outcomes

| **Reference** | **No of included studies in MA** | **Intervention** | **Study population: next of kin of people with…** | **Reported Effects on Outcomes** |
| --- | --- | --- | --- | --- |
| Baruch et al (2018) | 8 | “Psychosocial interventions”  single, family or group psychoeducation | Bipolar disorders | **Global burden**  at post treatment (6 studies, N=379)  **Knowledge**  at post-treatment (4 studies, N = 183)  at follow-up (3 studies, N = 127) |
| Hansen et al. (2022) | 31 in total  (15 for SMI) | “Mental health interventions on psychological distress”   - single & group Psychoeducation - Individual Therapy (CBT & other) - stress management - support groups - other psychosocial interventions - mindfullness interventions | Mental illnesses, mostly   - dementia/Alzheimer (k=17) - schizophrenia (k=9) | **Psychological distress**   - passive controls (31 RCTs, n=1899) - active controls (10 RCTs, n=484) - SMI (15 RCTs, n=823) - dementia/Alzheimer (17 RCTs; n=1049) - individual formats (12 RCTs, n=828) - group formats: (19 RCTs, n=944) - manualized interventions ≥ 8 weeks (20 RCTs, n=1049) |
| Ma et al. (2017) | 9 | “Family interventions targeting the needs of caregivers”   - mutual support groups - psychoeducation - adopted self-help - clinician-supported bibliotherapy | Recent-onset psychosis | **Burden**   - at 0-3m Follow-up (4 RCTs, n=312) - at 3-6m Follow-up (3 RCTs, n=256) - at >6-12 m Follow-up (3 RCTs, n=264); effects enhancing over time   **Family functioning**   - at 6-12m Follow up (3 RCTs, n=287)   **Formal support services utilization**   - at 12-24m Follow-up (3 RCTs, n=337)   Mutual support groups are more effective than psychoeducation |
| Rushton et al. (2023) | 10 | Psychological interventions   - cognitive-behavioural programs (group & family) - group support/psychoeducation programs - Mutual support programs - positive psychology and emotion regulation training - coping skills program | Substance use disorders | **Depression**   - individual Interventions (3 RCTs, N=237) - group Interventions (3 non-RCTs + 1 pre-post designs, N=42)   **Distress**   - individual interventions (3 RCTs, N=237 + 1 non-RCT, N=145) - Group Interventions (1 RCT, N=30 + 2 pre-post designs, N=188)     **Family functioning**   - individual interventions (2 pre-post studies, N=90) - group intervention (1 RCT, N=83)   **Coping**   - Iindividual Interventions (2 non-RCTs, N=90) - group interventions (1 RCT, N=83 + 2 non-RCTs, N=161/641 + 3 pre-post designs, N=302) |
| Sin et al. (2017) | 32 | Psychoeducation   - single, group, & web-based - no programs without actual interaction | Psychosis | **Global morbidities** (7 RCTs, n = 656)  **Depression** (2 RCTs, n =245)  **Perceived burden**   - at post-treatment (10 RCTs, n = 878) - at 6m Follow-up (10 RCTs, n = 821)   **Negative caregiving experiences** (5 RCTs, n =446)  **Expressed emotion** (5 RCTs, n= 337)  No moderation of effects by contact time, intervention duration and mode of delivery |
| Van Es et al. (2023) | 16 | Multi-family therapy   - multi-family therapy programs - psychoeducation MFT | Psychiatric Disorders, mostly   - schizophrenia/psychosis (k = 9; 29%) - mood problems (k = 5; 16%) - conduct problems (k = 5; 16%) | **Family functioning** (7 studies, N=621) |
| Wang et al. (2021) | 28 | Peer-Support   - self-help groups - internet support groups - individual peer support | SMI | **Community support service utilization** (4 pre-post studies, N=481 (families) |
| Zinser et al. (2022) | 15 | Multi-family therapy   - Inpatient & outpatient multi-family therapy programs | Eating Disorders | No significant effects on next of kin |
